# Non-Catalytic Inhibitors of the p38/MK2 Interface: Repurposing Approved Drugs to Target Neuroinflammation in Alzheimer’s Disease

**DOI:** 10.1021/acs.jmedchem.5c01425

**Published:** 2025-12-05

**Authors:** Maylynn Hu, Andrew Li, Payton Fleming, Julia Gralla, Kristos Negrón Terón, Ying Zhou, Eric J. Miller, Tyler S. Beyett, Zhexing Wen, Yuhong Du, Haian Fu, Andrey A. Ivanov

**Affiliations:** † Department of Pharmacology and Chemical Biology, 1371 Emory University School of Medicine, Emory University, Atlanta, Georgia 30322, United States; ‡ Department of Psychiatry & Behavioral Sciences Emory University School of Medicine, Atlanta, Georgia 30322, United States; § Winship Cancer Institute, Emory University, Atlanta, Georgia 30322, United States; ∥ Departments of Cell Biology, Neurology, and Human Genetics, 12239 Emory University School of Medicine, Atlanta, Georgia 30322, United States; ⊥ Emory Chemical Biology Discovery Center, Emory University School of Medicine, Emory University, Atlanta, Georgia 30322, United States; # Department of Hematology & Medical Oncology, Emory University, Atlanta, Georgia 30322, United States

## Abstract

Neuroinflammation is a key driver of Alzheimer’s disease and an emerging therapeutic target. The p38/MK2 pathway regulates microglial cytokine production, yet previous attempts have not yielded modulators with clinically suitable properties. Here, we apply an integrative structure-guided and screening strategy to identify small-molecule disruptors of the p38/MK2 protein–protein interaction (PPI). Virtual screening of FDA-approved drugs prioritized nilotinib, a BCR-ABL inhibitor, as a putative PPI disruptor. Biochemical and molecular dynamics analyses confirmed that nilotinib binds to p38, blocks MK2 association, and suppresses cytokine release in microglia. Guided by these findings, we developed a lysate-based TR-FRET ultrahigh-throughput assay that identified additional inhibitors, including α_1_-adrenergic antagonists doxazosin, terazosin, and alfuzosin. These compounds suppressed cytokine induction via docking groove blockade. Together, these results establish a non-ATP-competitive approach for selectively targeting the p38/MK2 complex and highlight the translational potential of drug repurposing to modulate neuroinflammation in Alzheimer’s disease.

## Graphical abstract

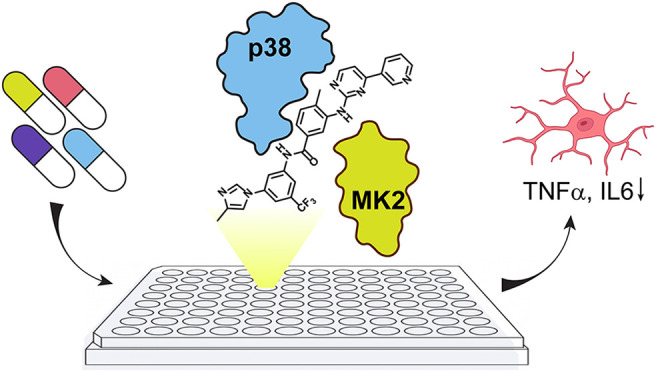

## Introduction

Alzheimer’s disease (AD) is a progressive neurodegenerative disorder and the leading cause of dementia, currently affecting more than six million individuals globally.^1^ Although characterized by the accumulation of β-amyloid (Aβ) plaques and hyperphosphorylated tau tangles, therapeutic strategies targeting Aβ clearance have yielded limited clinical benefit despite substantial plaque reduction.^2–4^ These findings underscore the urgent need to explore alternative, druggable mechanisms.

Genetic, transcriptomic, and histopathological evidence implicates chronic microglial activation in disease progression. In AD brains, microglia adopt a proinflammatory phenotype marked by sustained expression of cytokines such as tumor necrosis factor-α (TNF-α), interleukin-1β (IL-1β), and IL-6, which contribute to synaptic dysfunction, neuronal loss, and cognitive decline.^5–11^ IL-1β promotes synaptic damage through prostaglandin E_2_ and NMDA receptor signaling,^12^ while TNF-α induces apoptosis via TNF receptor 1.^13^ Persistent inflammation further exacerbates hallmark pathologies, including Aβ deposition and tau hyperphosphorylation, ultimately producing a systems-wide neurodegenerative cascade.^14^ Longitudinal studies suggest that inflammation precedes overt pathology, supporting its role as both a driver and a therapeutic entry point.^15^ Together, chronic inflammation is now recognized as a central driver of AD pathogenesis^16,17^ and other neurodegenerative disorders,^18,19^ and targeting neuroinflammation has emerged as a promising therapeutic strategy.^16,20–22^

The p38 mitogen-activated protein kinase (MAPK) pathway is central for microglia-mediated inflammation.^23–26^ Among the four p38 isoforms, p38α (MAPK14) is the most abundant and functionally relevant in the central nervous system (CNS).^27,28^ Activated by upstream kinases MKK3/6 in response to stress stimuli such as cytokines, lipopolysaccharide (LPS), and reactive oxygen species, p38α is elevated in AD brain tissue and correlates with Aβ plaques and neurofibrillary tangles.^29,30^ A major p38 downstream effector, MAPK-activated protein kinase 2 (MAPKAPK2 or MK2), amplifies inflammatory responses by stabilizing cytokine transcripts and modulating transcriptional programs.^23^ MK2 regulates proinflammatory cytokines, including TNF-α, IL-1β, and IL-6 ^31^ and plays a key role in chronic inflammation and AD pathology.^32,33^ MK2 expression and phosphorylation are markedly upregulated in neurodegenerative contexts and correlate with cytokine output, microglial activation, and Aβ burden.^32^ In contrast, MK2-deficient microglia and macrophages exhibit altered phenotypes and a reduction in proinflammatory cytokine and chemokine production.^32,34^ Thus, p38-mediated phosphorylation of MK2 is considered a critical driver of disease progression.^27,33,35,36^ Together, these data position both p38 and MK2 as promising therapeutic targets for intervention in AD.

Genetic or pharmacologic inhibition of p38 or MK2 suppresses proinflammatory cytokine expression and improves cognitive outcomes in preclinical models.^37–39^ However, clinical translation has been limited. First-generation ATP-competitive p38 inhibitors, such as VX-745 and MW01–2069A-SRM, showed promise in reducing MK2 phosphorylation and cytokine levels but failed in clinical trials due to poor tolerability and systemic toxicities,^38,40^ likely attributable to the inhibition of >100 p38 substrates.^41^ Targeting MK2 directly was proposed as a more selective strategy.^33,42^ Next-generation inhibitors such as CDD-450 (ATI-450), which bind in the ATP pocket of MK2 when in complex with p38, offered improved selectivity, but were terminated in trials for lack of efficacy.^43^ In contrast, MMI-0100 is a cell-permeable peptide derived from the MK2 nuclear export signal that functions as a substrate-competitive inhibitor, blocking MK2-mediated phosphorylation events without targeting the ATP site.^39,44^ While MMI-0100 has shown *in vivo* efficacy in models of AD and inflammation, peptide-based agents face challenges related to delivery and stability. Moreover, most small-molecule MK2 inhibitors continue to target the conserved ATP-binding cleft, which can complicate kinase selectivity and, for some chemotypes, is associated with limited solubility.^33,44^ These limitations underscore the need for alternative, noncatalytic strategies to modulate the p38/MK2 axis.

Targeting the protein–protein interaction (PPI) between p38 and MK2 offers an orthogonal approach that could retain therapeutic benefits while minimizing off-target effects. The p38/MK2 complex forms through a defined docking groove interaction distinct from the kinase active site, making it an attractive site for modulation.^45,46^ Recent advances in structure-based design and high-throughput PPI screening have fueled interest in targeting such interfaces for therapeutic intervention in human diseases.^47–49^

Here, we apply a structure-guided screening strategy to identify clinically actionable small molecules that disrupt the p38α/MK2 interaction and suppress proinflammatory signaling in microglia. We developed a physiologically relevant, lysate-based, time-resolved fluorescence resonance energy transfer (TR-FRET) assay to monitor native complex formation and screened a curated library of FDA-approved compounds. Computational modeling and biophysical validation identified nilotinib and several α_1_-adrenergic antagonists as direct PPI disruptors that bind to the p38 docking groove, inhibit MK2 recruitment, and suppress cytokine expression in microglial models.

These findings highlight the potential of noncatalytic, PPI-targeting approaches to modulate neuroinflammatory signaling in AD and underscore the translational value of drug repurposing combined with structure-guided discovery.

## Results

### Structure-Guided Feasibility of Disrupting the p38/MK2 Complex

In this study, we focused our analysis on the p38α isoform, encoded by the MAPK14 gene. For simplicity, we use the term “p38” throughout the text unless otherwise specified. Motivated by the limitations of existing clinical p38 or MK2 inhibitors, we evaluated the structural feasibility of disrupting the p38/MK2 interface with small molecules. Prior structural work suggested that the engagement of MK2 C-terminal residues Asp366-Ala390 by the p38 docking groove (DG), formed primarily by p38 residues 110–130 and 158–162, is critical for high-affinity p38/MK2 interaction.^45,46^ Accordingly, we focus our analysis on the p38 DG region.

PPI interfaces are often challenging to target. Therefore, we first evaluated the DG ligandability and then used short MK2-derived peptides as mechanistic probes to test the druggability of the complex prior to a small-molecule screening. To assess the contribution of the MK2 C-terminal residues, we conducted computational alanine scanning over the MK2 surface. Three regions, including MK2 72–79, 226–247, and 345–400, showed notable sensitivity to alanine substitutions with reduced computed binding affinity to p38 (Figure S1). These findings are consistent with previous biochemical studies and support the critical role of these interface regions in stabilizing the complex.^45,46^

Guided by the p38/MK2 cocrystal structure (Figure 1A), we next evaluated short MK2 C-terminal fragments for their capacity to engage the p38 DG. Using MM-GBSA binding free energy (Δ*G*_bind_) calculations, we determined that MK2 peptides 345–400, 370–393, and 369–382 can bind favorably to p38 with Δ*G*_bind_ of −176.6, −129.5, and −81.7 kcal/mol, respectively.

**Figure 1:**
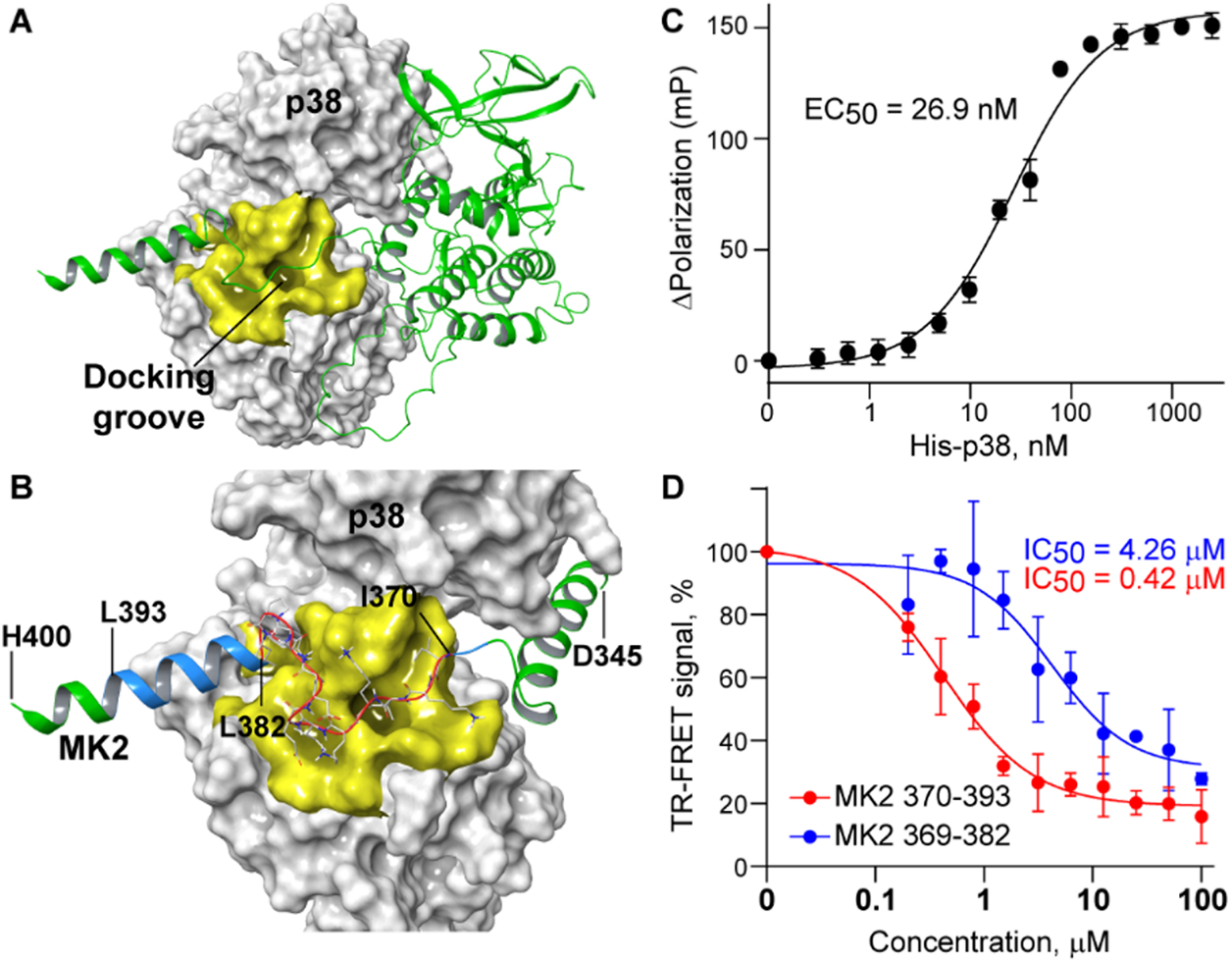
Structure-guided mapping and peptide validation of the p38/MK2 interaction interface. (A) The cocrystal structure of the p38/MK2 complex (PDB ID: 6TCA). The molecular surface of p38 is shown in gray. The p38 docking groove is highlighted in yellow. MK2 is shown as green ribbons. (B) The MK2 D345-H400 docking motif bound to the p38 docking groove is colored based on its fragments tested in this study: D345-H400 is colored in green, I370–L393 in blue, and I370-L382 in red. (C) The binding curve from a fluorescence polarization assay showing high-affinity binding of FITC-labeled MK2 370–393 peptide to His-tagged p38 (EC_50_ = 26.9 nM). (D) Dose–response curves from TR-FRET inhibition assays demonstrating that both MK2 370–393 and 369–382 peptides disrupt the p38/MK2 complex (IC_50_ = 0.42 μM and 4.26 μM, respectively).

To functionally validate the druggability of the p38/MK2 interface, we employed peptide-based inhibition using FITC-labeled MK2-derived fragments. The FITC-MK2 370–393 peptide (Figure 1B) bound His-tagged p38 in a fluorescence polarization (FP) assay with an EC_50_ of 26.9 nM (Figure 1C) and inhibited the interaction between purified recombinant His-p38 and GST-MK2 proteins in a TR-FRET assay with an IC_50_ of 0.42 μM (Figure 1D). Notably, a truncated 13-residue peptide (FITC-MK2 369–382, Figure 1B) retained significant inhibitory activity (IC_50_ = 4.26 μM; Figure 1D), confirming that relatively short peptides can engage the DG and disrupt the complex.

However, the MK2 docking peptides have limited cell-permeability, and plasmid-based peptide expression can perturb the stress-dependent inflammatory pathways. Therefore, we used them solely as mechanistic probes and focused subsequent studies on the discovery of cell-active small-molecule DG disruptors.

### Virtual Screening of Approved Drugs Identifies Candidate Inhibitors

With the p38 DG validated as ligandable and disruptable, we performed a DG-centered virtual screen of 1,040 structurally diverse FDA-approved drugs from the Selleck Chemicals library (Table 1S). The docking grid was centered on the well-characterized p38 docking groove, the most validated MK2-binding site. Docking was performed using Glide, followed by MM-GBSA binding free energy (Δ*G*_bind_) calculations to estimate ligand-protein binding strength.

The resulting Δ*G*_bind_ values ranged from −75.37 to 16.15 kcal/mol, with a mean of – 36.24 kcal/mol and a standard deviation (SD) of 12.33 kcal/mol (Figure 2A, Table S1). To prioritize high-confidence binders, we selected 28 compounds with Δ*G*_bind_ values at least two SDs below the mean (i.e., <−60.90 kcal/mol, Figure 2A).

**Figure 2:**
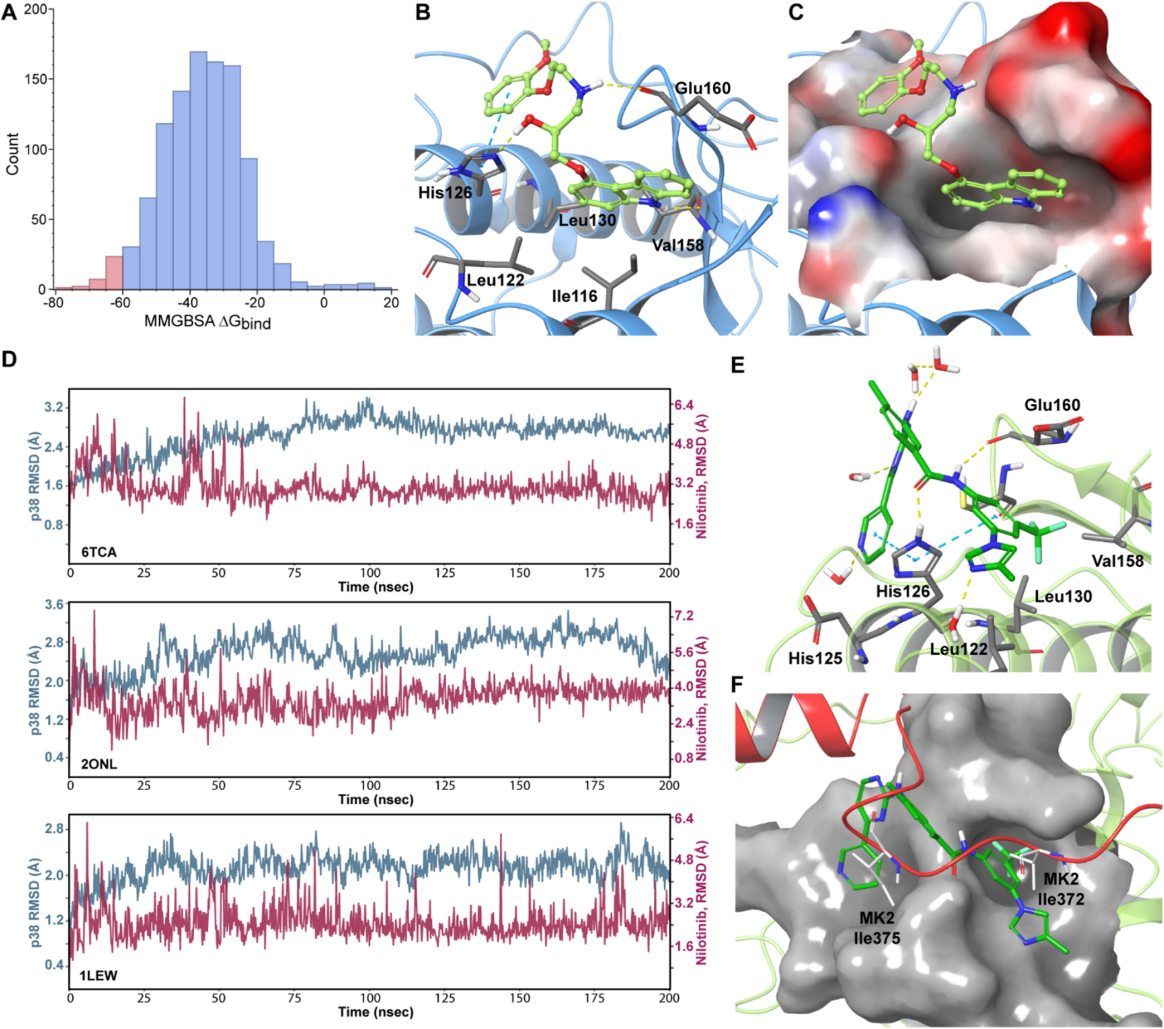
Virtual screening and molecular dynamics analysis identify nilotinib as a candidate p38/MK2 PPI inhibitor. (A) Distribution of MM-GBSA binding free energies (Δ*G*_bind_) from virtual screening of 1,040 FDA-approved drugs docked to the p38 docking groove. The p38 crystal structure (PDB ID: 6TCA) was used for the modeling studies. Compounds with Δ*G*_bind_ values more than two standard deviations below the mean (red bars, <−60.9 kcal/mol) were prioritized for further analysis. (B) Representative binding pose of carvedilol highlighting key interactions with the p38 docking groove, including hydrogen bonds with Val158, Glu160, and His126 (yellow lines), hydrophobic interactions with the nonpolar pocket defined by Ile116, Leu122, Leu130, and Val158, and a pi-pi stacking with His126 (cyan line). (C) Carvedilol’s carbazole moiety binds within the hydrophobic cleft of the p38 docking groove, which is shown as a molecular surface representation colored by electrostatic potential (red = negative, blue = positive). (D) Root-mean-square deviation (RMSD) plots from three 200 ns molecular dynamics simulations of the p38–nilotinib complex. The RMSD of protein backbone atoms is shown in aquamarine, and nilotinib in red. The PDB IDs of the p38 structures used for the modeling are indicated in the lower-left corners. (E) The representative binding pose of nilotinib obtained after 200 ns MD simulation (a final snapshot of one of the MDs), highlighting pi-pi and H-bond interactions with His126, the H-bonding with Glu160, and multiple water-bridged contacts that stabilize ligand orientation within the groove. (F) Structural overlay of the nilotinib–p38 complex with the p38/MK2 cocrystal structure, illustrating displacement of key MK2 anchoring residues Ile372 and Ile375 by nilotinib. P38 is shown as green ribbons, the p38 docking groove as gray molecular surface, and MK2 as red ribbons.

Since MM-GBSA energy alone may not accurately predict binding, we evaluated hydrogen bond formation to refine selection beyond free energy estimates. Hydrogen bonds are highly directional and distance-sensitive. Small networks of 2–3 complementary H-bonds can fix a single ligand orientation, whereas off-targets with mismatched polar atoms may incur desolvation penalties and show loss of binding. Thus, while hydrophobic contacts often drive affinity, H-bonding typically dictates specificity. Five of the 28 top-scoring compounds (astemizole, lumateperone, rilpivirine, squalane, and trazodone) lacked hydrogen bonds but were retained by hydrophobic and electrostatic interactions. Seven others (avapritinib, fedratinib, lumefantrine, pimozide, radotinib, tebipenem, and vorapaxar) formed only a single H-bond. Twelve compounds, including masitinib, ozanimod, afatinib, doxazosin, cabozantinib, domperidone, acalabrutinib, crizotinib, remogliflozin, selpercatinib, terfenadine, and tirbanibulin, formed two H-bonds. Five FDA-approved compounds (carvedilol, nebivolol, avanafil, barnidipine, and nilotinib) formed three or more hydrogen bonds within the p38 docking groove, suggesting enhanced anchoring and potential specificity. For example, carvedilol, a nonselective adrenergic receptor antagonist, engaged in hydrogen bonding with the backbone carbonyls of Val158 and Glu160, as well as the imidazole nitrogen of His126. The latter also formed a pi–pi stacking interaction with carvedilol’s methoxyphenyl ring (Figure 2B). In addition, the drug’s hydrophobic carbazole moiety occupied a deep nonpolar pocket defined by Ile116, Leu122, Leu130, and Val158 (Figure 2C).

### Molecular Dynamics Simulations Reveal Stable nilotinib Binding

Among the identified hits, nilotinib, a clinically approved BCR-ABL inhibitor, has the strongest preclinical evidence supporting its ability to modulate microglial activation and suppress proinflammatory cytokine production in models of neurodegenerative disease.^50,51^ Previous studies have suggested that nilotinib may act as a p38 kinase inhibitor.^52–54^ In contrast, our docking-based computational model suggested a previously unrecognized mechanism by which nilotinib may also bind directly to the p38 docking groove, disrupt MK2 recruitment, and thereby inhibit this critical proinflammatory signaling axis. To support this mechanism, we refined the p38-nilotinib model by an ensemble docking to three different p38 structures cocrystallized with MK2 (PDB ID: 6TCA,^55^ 2ONL^46^) and MEF2A (PDB ID: 1LEW^56^). We found that in each structure, nilotinib can adopt a similar binding mode coordinated by the H-bonds with His126 and Glu160 (Figure S2).

We further confirmed and refined the models by conducting 200 ns molecular dynamics (MD) simulations (Figure 2D). Interaction analysis over the whole MD trajectories across the three models revealed that, similar to carvedilol, nilotinib’s hydrophobic trifluoromethyl group occupied a nonpolar pocket defined by Leu116, Leu122, Leu130, and Val158 (Figure 2E). Notably, in the p38/MK2 cocrystal structure, this same pocket is occupied by Ile372 of MK2, one of the key anchoring residues that secures MK2 at the p38 docking groove^46^ (Figure 2F). The position of another critical MK2 residue, Ile375,^46^ was occupied by nilotinib’s pyridinyl–pyrimidine motif (Figure 2F), located between p38 His126 and Phe129. This orientation was further stabilized by a pi–pi stacking interaction with the imidazole side chain of His126, which also formed an H-bond with nilotinib’s amide oxygen atom. In addition, the side chain of Gln120 formed a hydrogen bond with the nilotinib amide NH group. (Figure 2E). These interactions were consistently observed in at least 30% of frames in each of the three models, underscoring their robustness.

### Cell-Based Validation of Nilotinib’s Mechanism of Action

To confirm direct binding to p38, we performed a thermal shift assay (TSA) using recombinant His-tagged p38. Nilotinib induced dose-dependent thermal stabilization (Δ*T*_max_ = 8.22 °C, Figure 3A), comparable to that observed with the type II p38 inhibitor SR318 (Δ*T*_max_ = 13.47 °C, Figure 3B), consistent with direct ligand binding. Then, using a TR-FRET competition assay, we confirmed that nilotinib dose-dependently inhibits the binding of His-tagged MK2 346–400 docking peptide to Venus-flag (VF)-tagged p38 in a cell lysate-based TR-FRET assay with the IC_50_ value of 4.0 μM (Figure 3C). These data indicate a displacement of the MK2 docking peptide, supporting the predicted binding mode of nilotinib at the p38 DG.

**Figure 3:**
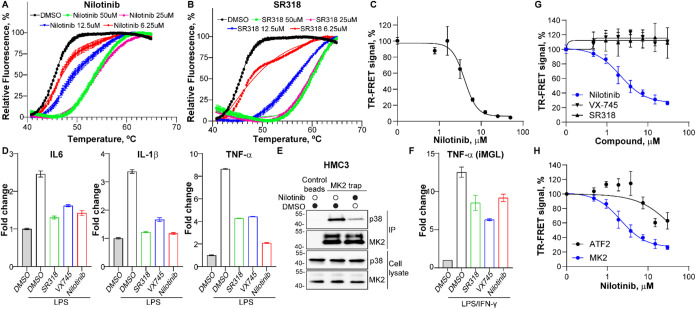
Validation of nilotinib as a p38/MK2 PPI Inhibitor. (A) Thermal shift assay (TSA) showing dose-dependent stabilization of recombinant His-tagged p38 by nilotinib (Δ*T*_max_ = 8.22 °C), consistent with direct binding. (B) TSA profile for SR318, a type II ATP-competitive p38 inhibitor, used as a positive control (Δ*T*_max_ = 13.47 °C). (C) Nilotinib competes with His-MK2 346–400 fragment for VF-p38 in a cell lysate-based TR-FRET assay. (D) Quantitative qRT-PCR analysis showing that nilotinib significantly (*p*-value <0.05) suppresses LPS-induced TNF-α, IL-6, and IL-1β expression in HMC3 microglial cells. P38 inhibitors SR318 and VX-745 were used as positive controls. (E) Nilotinib disrupts the endogenous p38/MK2 complex in HMC3 cells, as shown by coimmunoprecipitation, correlating with cytokine suppression. (F) qRT-PCR analysis showing that nilotinib suppresses LPS/IFNγ-induced TNF-α expression in the human iPSC-derived microglia (iMGL). (G) TR-FRET assay with recombinant p38 and MK2 proteins purified from *E. coli* demonstrated direct inhibition of the complex by nilotinib (IC_50_ = 2.2 μM). In contrast, ATP-site inhibitors VX-745 and SR318 failed to disrupt the interaction, supporting a non-ATP-competitive mechanism for nilotinib activity. (H) Nilotinib demonstrates a weak inhibition of p38/ATF2 PPI (IC_50_ > 30 μM, maximal inhibition ∼ 37%) in a TR-FRET assay with recombinant purified His-p38 and GST-ATF2. The inhibition of His-p38/GST-MK2 PPI by nilotinib was monitored in parallel.

Previous studies have shown that nilotinib suppresses LPS-induced production of TNF-α, IL-6, and other inflammatory cytokines in microglial cells.^50^ Consistent with these reports, we found that nilotinib significantly reduced LPS-stimulated expression of TNF-α, IL-6, and IL-1β in human HMC3 microglia (Figure 3D). This effect correlated with decreased p38/MK2 complex formation, as demonstrated by coimmunoprecipitation (Figure 3E). We further validated the anti-inflammatory activity of nilotinib by confirming suppression of LPS-induced TNF-α expression in human stem-cell-derived microglia (iMGL, Figure 3F).

To determine whether this effect was direct, we conducted a TR-FRET assay using recombinant p38 and MK2 proteins purified from *E. coli*. Nilotinib disrupted complex formation with an IC_50_ of 2.2 μM, indicating direct inhibition of the p38/MK2 interaction (Figure 3G). In contrast, ATP-competitive inhibitors such as VX-745 and SR318 failed to reduce the TR-FRET signal, suggesting that occupancy of the ATP-binding site is insufficient to disrupt the complex. These findings support a distinct, non-ATP-competitive mechanism for nilotinib, consistent with direct disruption of the p38/MK2 protein–protein interaction interface. We next compared nilotinib activity against p38 PPIs with MK2 and another well-known substrate, ATF2 (Figure 3H). In a side-by-side TR-FRET assay with purified recombinant His-p38, GTS-MK2, and GST-ATF2 (2h incubation) nilotinib demonstrated a significantly weaker inhibition of p38/ATF2 PPI (IC_50_ > 30 μM, maximal inhibition ∼ 37% at 30 μM), indicating ∼15-fold selectivity against MK2 (IC_50_ of 2.0 μM, maximal inhibition ∼ 75%). This data further supports the preferential direct disruption of the MK2 binding rather than a general perturbation of the p38 PPI through allosteric mechanisms.

### Structure–Activity Relationship of Nilotinib Analogs

To better understand the structural requirements for p38/MK2 PPI inhibition, we evaluated a panel of ten close analogs of nilotinib using the TR-FRET assay (Figure 4). Compounds 2–6 retained measurable activity, with IC_50_ values ranging from 3.0 to 21.3 μM, while compounds 7–10 exhibited less than 50% inhibition at 30 μM and were considered inactive. This sharp loss of activity in the latter group suggests specific structural dependencies critical for function. Each IC_50_ value was determined in triplicate and repeated in three independent experiments. The standard deviations were consistently within 10%, providing confidence that the observed differences in potency reflect true SAR trends rather than experimental noise.

**Figure 4:**
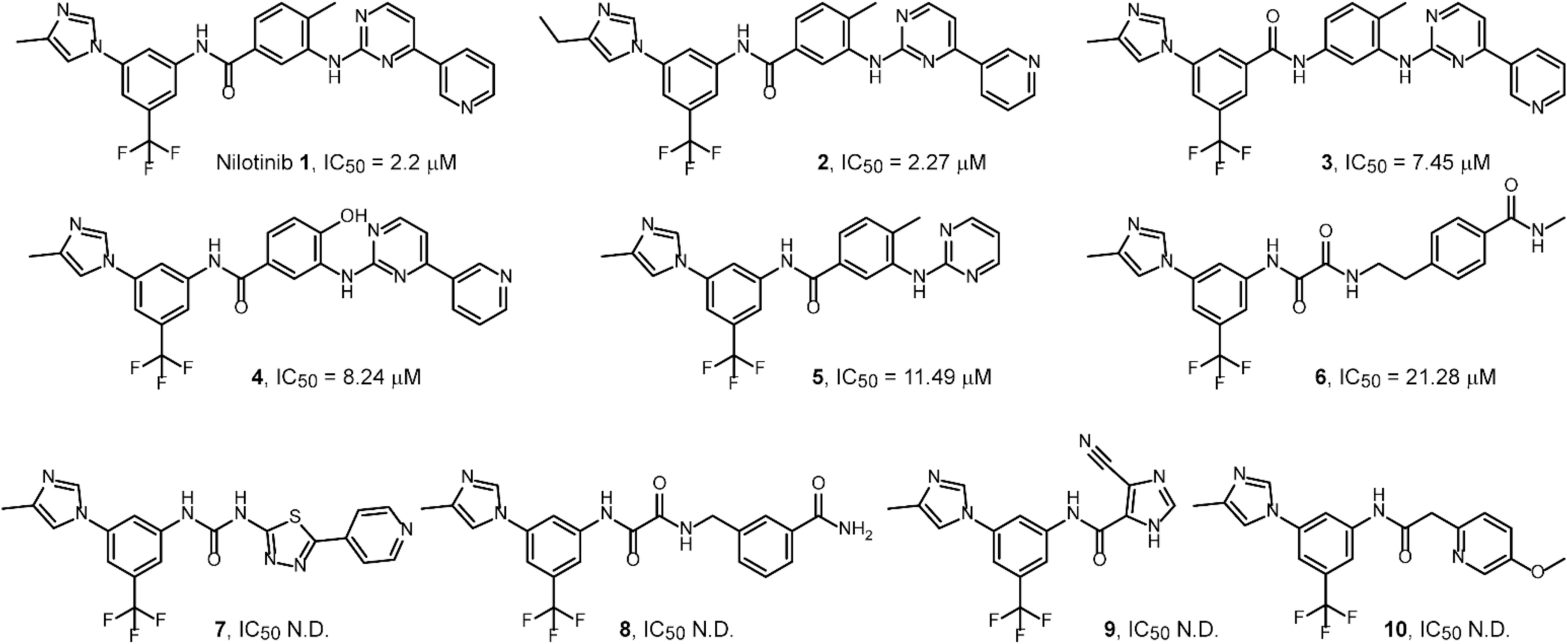
Chemical structures of nilotinib and ten analogs evaluated for p38/MK2 PPI inhibition using a TR-FRET assay with recombinant purified proteins. IC_50_ values are shown for compounds exhibiting measurable activity; compounds with less than 50% inhibition at 30 μM are indicated as not determined (N.D.).

To define the chemical features contributing to the potency of p38/MK2 PPI inhibitors, we docked the nilotinib analogs into the p38 docking groove (Figure 5A). Active compounds 1–6 adopted a similar orientation and exhibited better alignment within the p38 docking groove compared to inactive analogs 7–10. These structural differences were reflected by statistically significant variations in predicted binding energies (*p* = 0.01), with actives displaying a lower mean Δ*G*_bind_ (−54.2 ± 3.9 kcal/mol) than inactives (−45.9 ± 1.6 kcal/mol).

**Figure 5:**
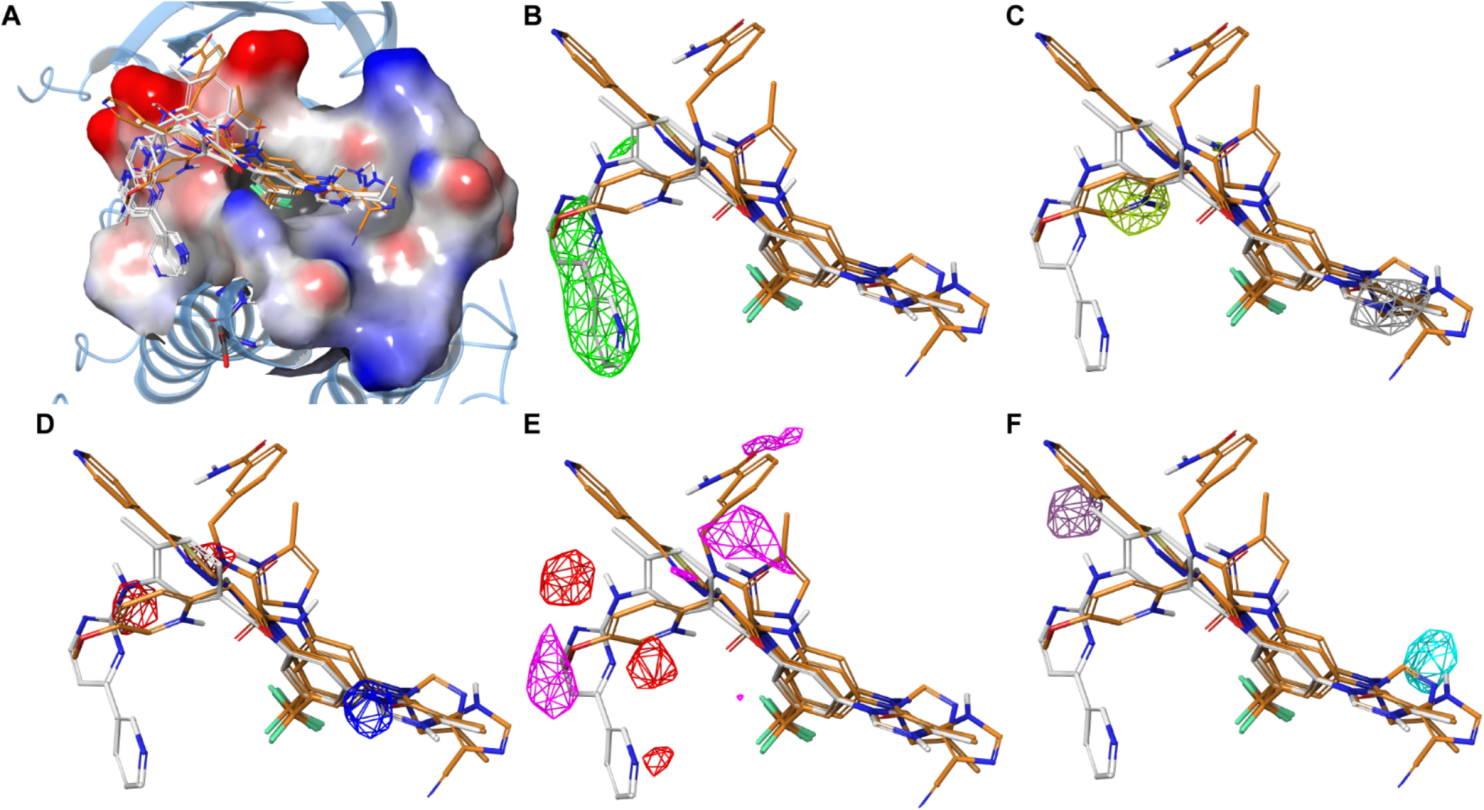
Field-based QSAR maps illustrating physicochemical features of nilotinib analogs associated with p38/MK2 PPI inhibition. (A) Compounds 1–6 (white) and 7–10 (orange) docked into the p38 binding groove. The molecular surface of the binding groove is colored based on the electrostatic potential, ranging from the most positive (blue) to the most negative (red) charge. (B) Steric field map showing regions where steric bulk is favorable (green). The pyridine–pyrimidine system is positioned within favorable steric zones, supporting its critical role in activity. (C) Hydrophobic field map with yellow-green and gray surfaces representing positive and negative hydrophobic contributions, respectively. (D) Electrostatic field map colored by potential (red - negative, blue - positive). (E) Hydrogen bond acceptor field map. Red contours indicate favorable contributions of H-bond acceptors, while the magenta contour indicates unfavorable contributions of H-bond acceptors. (F) Hydrogen bond donor field map. The blue-violet contour indicates the region favorable for H-bond donors. The cyan field map indicates the area unfavorable for the H-bond donors.

Averaged IC_50_ values from three independent experiments (Figure 4) were used to construct 3D QSAR models. Despite the limited data set size, the models captured key activity-associated features. The planar pyridine and pyrimidine moieties of nilotinib proved essential, as their complete removal in compounds 9 and 10 abolished activity, consistent with favorable steric field contributions around these fragments (Figure 5B). Replacement of the phenyl ring in the methylphenyl fragment with aliphatic chains or hydrophilic amino/amido groups reduced (6) or eliminated activity (Figure 5C). Conversely, increased hydrophobicity near the imidazole nitrogen was detrimental to potency (Figure 5C).

The model also captured electrostatic properties of the compounds, indicating that a more negative potential around the nitrogen of the nilotinib anilino-2-pyrimidine fragment and near the amino substituents of compounds 7–9 favors activity. Interestingly, docking of compound 9 revealed an inverted orientation relative to the larger analogs: its methyl-imidazole ring aligned toward nilotinib’s amido group, while the imidazole-carbonitrile moiety occupied the position of nilotinib’s methyl-imidazole. In this pose, the oxygen atom of 9 replaced the carbon atom of the imidazole in nilotinib, resulting in a more favorable positive electrostatic field at that position (Figure 5D).

Analysis of hydrogen-bond acceptor (Figure 5E) and donor (Figure 5F) fields further highlighted structural determinants of activity. H-bond acceptors near the amido oxygen of 8 or the methoxy group of 10 corresponded to unfavorable fields (Figure 5E), whereas the pyrimidine nitrogens in 1–5 were associated with enhanced potency. The amino group of the nilotinib anilino-2-pyrimidine fragment emerged as a key H-bond donor feature, while additional donors near the imidazole ring were detrimental (Figure 5F).

Together, these findings highlight the essential role of the conjugated pyridine–pyrimidine ring system and central amide linker in inhibiting the p38/MK2 interaction while showing that peripheral modifications at the terminal aryl and heterocyclic groups are tolerated. These SAR insights provide a foundation for the future rational design of p38/MK2 PPI inhibitors with improved potency.

### High-Throughput Screening Identifies Additional Inhibitors

Although nilotinib validated the feasibility of disrupting the p38/MK2 PPI, its limited CNS exposure and BBB permeability may constrain its translational potential for neurological disorders. We therefore sought alternative chemical scaffolds with potentially improved brain-associated pharmacology. To this end, we conducted a high-throughput screening (HTS) campaign to identify additional small molecules capable of disrupting the p38α/MK2 interaction. With this aim, we developed a cell lysate-based TR-FRET HTS platform. By preserving native folding and post-translational modifications, the lysate-based assay allows for the detection of physiologically relevant protein–protein interactions that may not be fully recapitulated using recombinant proteins. This design improves biological relevance and increases the likelihood of identifying functionally active inhibitors.

Previous studies suggested that p38α and p38β are the principal binding partners of MK2, whereas p38γ and p38δ interact only weakly or not at all.^45,46^ To assess whether our TR-FRET assay could resolve these isoform-specific differences, we systematically compared MK2 interactions across the four p38 isoforms. Using conventional Flag-immunoprecipitation (Flag-IP) in HEK293T cells, we first confirmed selective binding of GST-MK2 to Flag-tagged p38α and p38β, but not p38γ or p38δ (Figure 6A). We then validated these findings with the TR-FRET assay, which similarly showed preferential binding of MK2 to p38α and p38β, with negligible interaction with p38γ or p38δ (Figure 6B).

**Figure 6:**
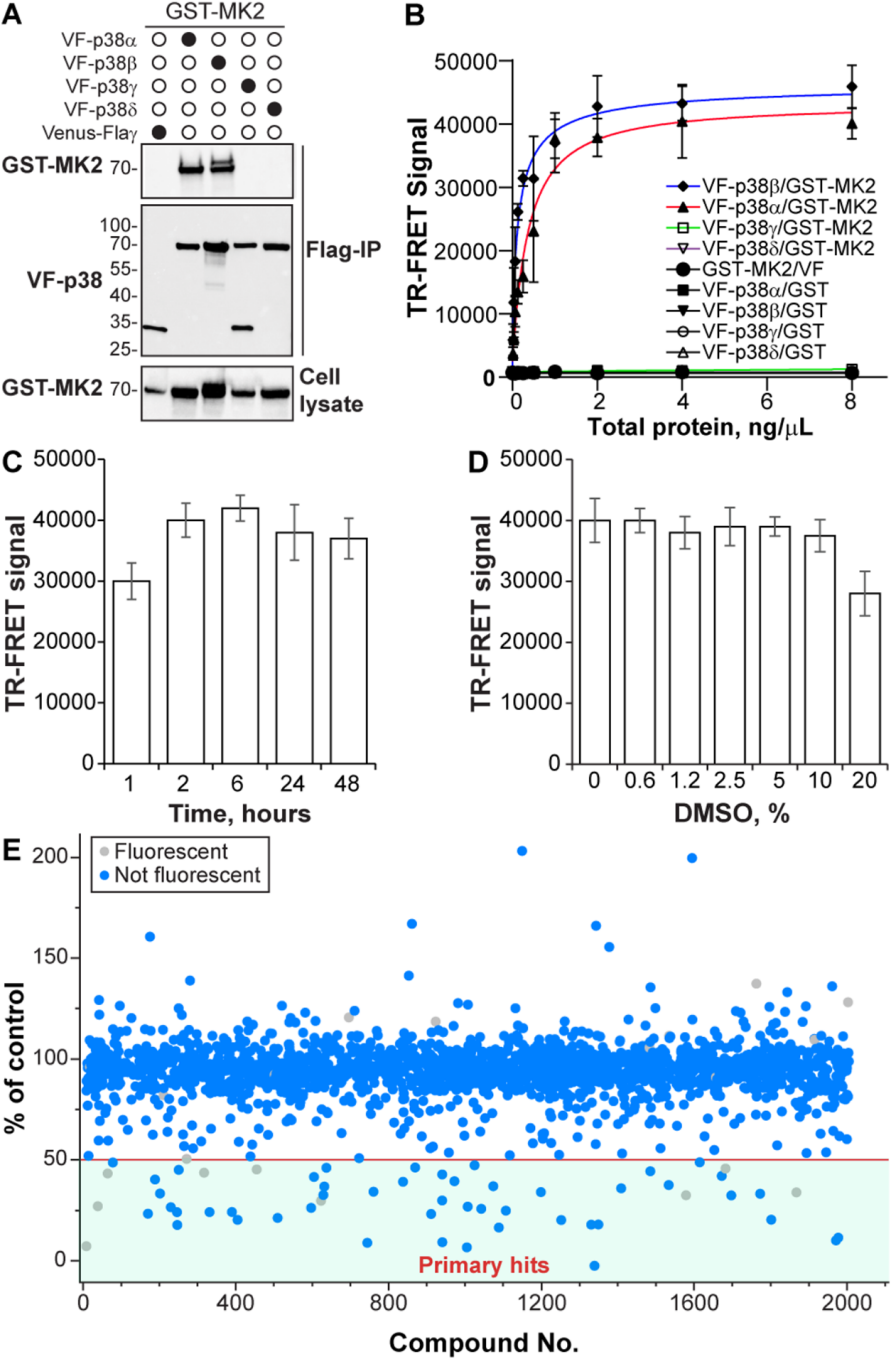
Development of a lysate-based TR-FRET platform for high-throughput screening of p38/MK2 PPI inhibitors. (A) The preferential binding of MK2 to p38α and p38β isoforms was determined by Flag-immunoprecipitation in HEK293T cells. (B) Isoform selectivity of MK2 binding was validated by TR-FRET using lysates coexpressing GST-tagged MK2 and Venus-Flag (VF)-tagged p38 isoforms. Robust signal was observed for p38α and p38β, with negligible interaction detected for p38γ and p38δ. (C) TR-FRET assay shows stable signal over 48 h postantibody addition, indicating excellent temporal stability. (D) The platform tolerates up to 10% DMSO without signal degradation, supporting its suitability for screening applications. (E) Pilot screen of 2036 compounds from the Emory Enriched Library (EEL) in 1536-well format identified 48 compounds that inhibited the p38/MK2 interaction by ≥ 50% relative to vehicle control. Gray dots indicate fluorescence assay-interfering compounds.

Together, these results confirm the expected biological selectivity of MK2 binding and establish the robustness of the TR-FRET assay.

The established TR-FRET assay demonstrated excellent temporal stability, with signal retention for up to 48 h postantibody addition (Figure 6C), and was highly tolerant to solvent conditions, maintaining integrity in the presence of up to 10% DMSO (Figure 6D). These characteristics made the platform suitable for screening in 384-well format, which we further miniaturized to the 1,536-well ultra-HTS (uHTS) format. Performance comparisons confirmed assay equivalency between the two formats, with S/B > 20 in both configurations (Figure S3).

Using the optimized 1,536-well platform, we conducted a pilot screen of 2,036 compounds from the Emory Enriched Library (EEL), which includes pharmacologically annotated molecules and diverse chemotypes with CNS-relevant properties (Table S2). Primary screening identified 48 compounds, including nilotinib, that inhibited the p38/MK2 interaction by ≥ 50% relative to the DMSO control (Figure 6E), without the significance of fluorescence interference (0.5 < 620 nm FOC < 1.5). Among these, 11 FDA-approved drugs demonstrated potent activity. These included pranlukast (93% inhibition), alfuzosin (82%), terazosin (77%), bexarotene (74%), rofecoxib (73%), candesartan cilexetil (68%), doxazosin (58%), apomorphine (56%), closantel (53%), amphotericin B (51%), and oxytetracycline (51%).

We noted that three of the top hits, alfuzosin, terazosin, and doxazosin, are α_1_-adrenergic receptor antagonists with closely related chemical scaffolds, raising the possibility of a shared mechanism of action (Figure 7A). Identifying structurally related actives in phenotypic or PPI-based screens often reflects true biological target engagement, reinforcing the credibility of the screening results. Moreover, doxazosin had been prioritized independently through our computational pipeline, providing orthogonal support for its relevance as a p38/MK2 PPI inhibitor. These three compounds were selected for secondary validation in dose–response and mechanistic assays.

**Figure 7:**
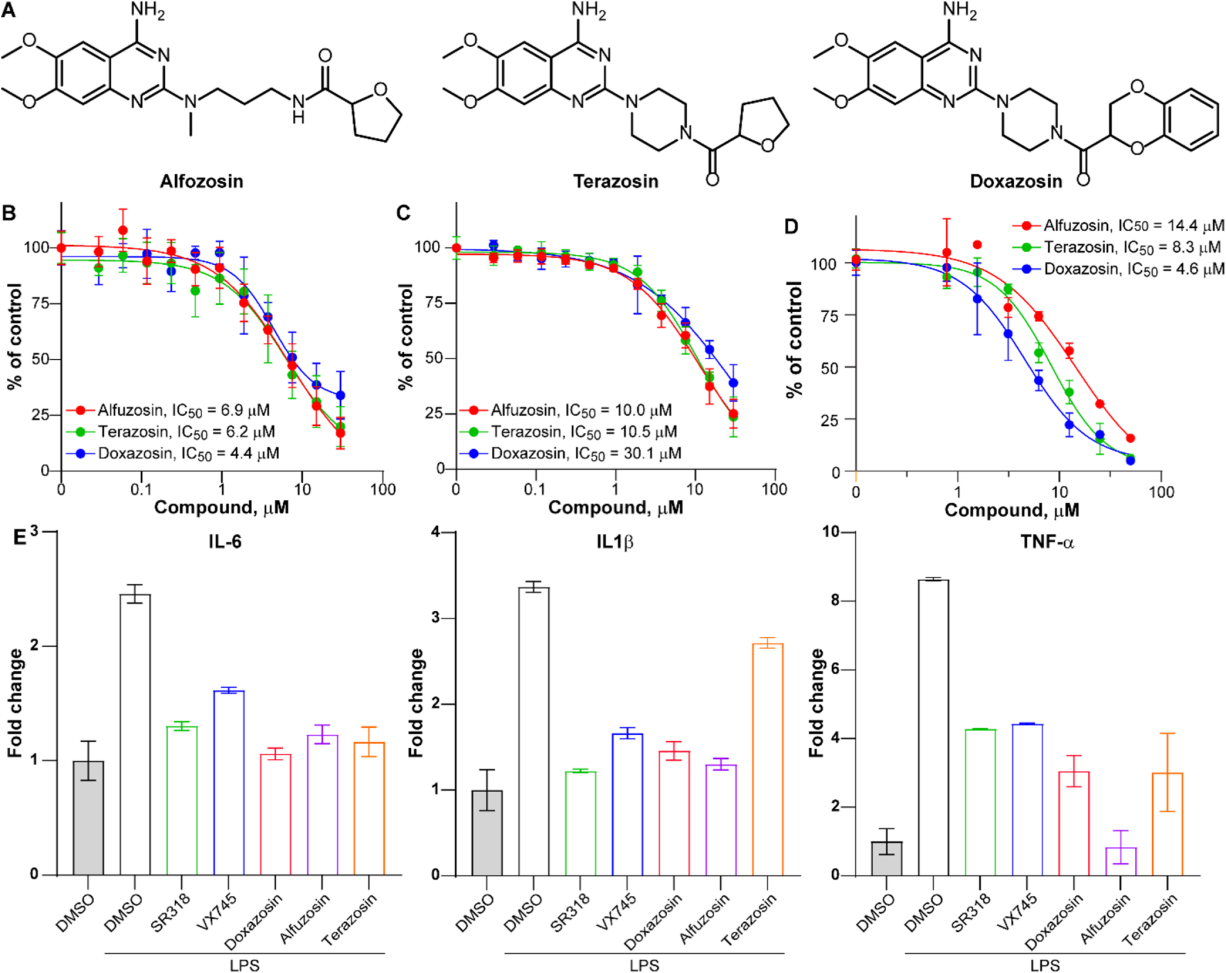
**α**_**1**_-Adrenergic antagonists disrupt the p38/MK2 interface and suppress cytokine production in microglial cells. (A) Chemical structures of doxazosin, terazosin, and alfuzosin, three α_1_-adrenergic receptor antagonists identified from the high-throughput screen. (B) Dose–response TR-FRET assays using recombinant purified p38 and MK2 proteins demonstrate that all three compounds inhibit the p38/MK2 protein–protein interaction, with IC_50_ values of 4.4 μM (doxazosin), 6.2 μM (terazosin), and 6.9 μM (alfuzosin). (C) The compound activity was confirmed in a cell lysate-based TR-FRET format, showing a moderate reduction in potency relative to the recombinant protein assay. (D) In a complementary TR-FRET assay using HEK293T lysates coexpressing VF-tagged p8 and a His-tagged MK2 346–400 docking peptide, all three α_1_-antagonists and nilotinib dose-dependently disrupted peptide binding to p38, consistent with direct competition at the docking interface. (E) qRT-PCR analysis in HMC3 microglial cells shows that all three compounds significantly (*p*-values <0.05) suppressed LPS-induced expression of TNF-α, IL-6, and IL-1β, similarly to known p38 inhibitors SR318 and VX745, demonstrating effective functional inhibition of p38/MK2 signaling in a disease-relevant context.

### α_1_-Adrenergic Antagonists Disrupt the p38/MK2 Interface and Suppress Microglial Cytokine Production

To validate the activity of top candidates identified through high-throughput screening, we performed dose–response TR-FRET assays using freshly repurchased compounds. All three α_1_-adrenergic antagonists demonstrated p38/MK2 PPI inhibitory activity in the recombinant protein assay, with IC_50_ values of 4.4 μM for doxazosin, 6.2 μM for terazosin, and 6.9 μM for alfuzosin (Figure 7B). As expected, potency was moderately reduced in the cell lysate-based TR-FRET format, with IC_50_ values of 10.5 μM for terazosin, 10.0 μM for alfuzosin, and 30.1 μM for doxazosin (Figure 7C). These findings are consistent with the primary HTS results, which showed comparatively weaker potency for doxazosin than the other two compounds.

To determine whether these compounds directly target the canonical p38 docking groove, rather than exerting indirect or nonspecific effects, we employed a complementary TR-FRET assay using HEK293T lysates coexpressing VF-tagged p38 and a His-tagged MK2 346–400 docking peptide.

This system was specifically designed to monitor interactions mediated by the p38 docking groove and MK2 346–400 docking motif, independently of kinase activity or ATP binding. In this format, doxazosin, terazosin, alfuzosin, and nilotinib each significantly reduced peptide binding to p38 in a dose-dependent manner (Figure 7D). These results support a mechanism in which the compounds competitively interfere with the docking interface, thereby preventing MK2 recruitment and providing a non-ATP-competitive strategy to selectively modulate the p38/MK2 axis. Computational docking further supported these findings by demonstrating that doxazosin, terazosin, and alfuzosin can favorably bind to the p38 docking groove with the MM-GBSA Δ*G*_bind_ values of −53.14, −55.05, and −57.22 kcal/mol, respectively (Figure S4). All three ligands adopted highly similar orientations, with conserved hydrogen bonding to Gln120 and pi–pi stacking with His126, as was observed for nilotinib.

To assess the functional consequences of PPI disruption, we next examined the impact of these compounds on proinflammatory cytokine production in human microglial HMC3 cells. Cells were stimulated with LPS to induce innate immune activation, and expression levels of TNF-α, IL-6, and IL-1β were measured by quantitative RT-PCR. All three α_1_-blockers significantly suppressed cytokine induction (Figure 7E), with reductions comparable to those observed for nilotinib-treated cells (Figure 3C). These findings indicate that pharmacologic disruption of the p38/MK2 interaction is sufficient to attenuate proinflammatory signaling in a disease-relevant cellular context.

Together, these results provide compelling mechanistic evidence that nilotinib, doxazosin, terazosin, and alfuzosin directly bind the p38α docking groove, disrupt MK2 recruitment, and block downstream cytokine expression. Identifying structurally related, approved α_1_-adrenergic antagonists that inhibit p38α/MK2 complex formation and downstream inflammatory output underscores the translational potential of repurposing these drugs as modulators of neuroinflammation. These findings further validate the utility of our integrative screening platform in identifying functionally relevant PPI inhibitors with therapeutic promise in neurodegenerative disease.

## Discussion

The p38/MK2 signaling axis regulates proinflammatory cytokine production and RNA-binding protein activity in both immune cells and neurons. In microglia, this pathway drives the expression of TNF-α, IL-6, and other inflammatory mediators that contribute to Alzheimer’s disease-associated neuroinflammation, synaptic dysfunction, and dendritic spine loss, ultimately impairing cognitive performance and plasticity.^37^ Inhibition of this axis reduces neuroinflammatory signaling and improves memory in multiple AD models, highlighting its therapeutic relevance.^57^

Despite this strong biological rationale, clinical development of ATP-competitive inhibitors targeting p38 or MK2 has been hampered by pharmacological limitations. Early p38 inhibitors, such as SB203580 and its derivatives (e.g., SB-242235, PH-797804), showed efficacy in preclinical models but lacked kinase selectivity due to engagement of the highly conserved ATP-binding cleft, leading to off-target inhibition and dose-limiting toxicities.^58^ Second-generation compounds like CBS-3595, a dual PDE4/p38α inhibitor, offered modest improvements but still suffered from hERG inhibition and sex-specific pharmacokinetics that restricted further advancement.^58^

Complex-selective inhibitors were designed to address these issues. CDD-450 targets the composite ATP site at the p38/MK2 interface, preventing MK2 phosphorylation while sparing other p38 substrates such as ATF2 and PRAK.^43^ Its SAR-guided optimization yielded Compound 36, with improved potency and stability.^59^ However, because these inhibitors still occupy the ATP-binding cleft, they may affect other p38 functions critical for neuronal homeostasis.^37^ Clinically, the benefit of p38 inhibition in AD remains uncertain. For example, neflamapimod (VX-745), a brain-penetrating p38 inhibitor, failed to improve cognition in mild AD patients despite reducing CSF tau and p-tau181 levels in a Phase II trial.^60^

To address these limitations, our study targets the p38/MK2 protein–protein interaction (PPI) interface as a mechanistically distinct strategy that avoids catalytic site engagement. Structure-guided modeling identified the p38α docking groove as a tractable interface, which we validated through alanine scanning, competitive peptide inhibition, and lysate-based TR-FRET assays. Although the docking peptides have limited cell-permeability and limited potential for functional cell-based studies, they served as valuable mechanistic probes to validate DG disruptability. These peptides also provide starting points for structural optimization toward cell-active mimetics, which we plan to pursue in future work.

Virtual screening of FDA-approved drugs identified nilotinib, a BCR-ABL inhibitor, as a novel disruptor of this interface. Previous work showed that nilotinib suppresses p38 phosphorylation in muscle stem cells, impairing myogenic differentiation and altering ERK1/2 and AKT signaling.^61^ More recently, it was demonstrated that nilotinib mitigates LPS-induced neuroinflammation and memory impairment in wild-type mice by inhibiting p38 and STAT3 phosphorylation in microglia and astrocytes, resulting in decreased expression of IL-1β, IL-6, and C7OX-2.^54^ These findings implicate p38 as a biologically relevant target of nilotinib in both peripheral and CNS tissues.

Nilotinib was designed as an ATP-competitive type-II kinase inhibitor.^62^ Consistent with this, X-ray structures captured nilotinib in the ATP sites of p38α (PDB ID: 3GP0, unpublished), and p38β (PDB ID: 9CJ1 ^63^). In systematic kinase profiling, nilotinib bound p38α with a *K*_d_ of 100 nM yet inhibited its catalysis only weakly (IC_50_ ≈ 2.2 μM). This *K*_d_/IC_50_ divergence is consistent with indirect effects on substrate phosphorylation (e.g., interference with docking interactions), although those assays do not localize the binding site. Our results provide new insight by showing that nilotinib can also engage the p38 docking groove, interfering with the p38 and MK2 interaction. Such an alternative occupation of distinct binding sites by the same ligand was reported previously for different protein–ligand pairs. For instance, a CK2 inhibitor 5,6-dichloro-1-β-d-ribofuranosylbenzimidazole (DRB) can simultaneously bind to the CK2 ATP-binding site and a distinct allosteric site at the interface of the CK2α/CK2β interaction (PDB ID: 3BW5).^64^ The crystallographic studies also showed the dual binding of 4-[3-(4-fluorophenyl)-1H-pyrazol-4-yl]­pyridine (4-FPP) to the p38 ATP binding site and its distinct C-terminal allosteric site (PDB ID: 3HVC),^65^ implicated in the recognition of FXF docking.^66^

The non-ATP-competitive mechanism of p38/MK2 PPI regulation can enable the inhibition of neuroinflammatory signaling while preserving p38’s catalytic function and alternative interactions critical for neuronal viability. At the same time, nilotinib exhibits limited CNS exposure/BBB permeability, which constrains its translational potential for neuroinflammatory indications. These considerations motivated us to discover new classes of p38/MK2 PPI inhibitors with improved brain-associated pharmacology.

To assess the p38/MK2 PPI druggability in a cellular context, we implemented a physiologically relevant TR-FRET platform using native lysates to maintain endogenous protein conformation and post-translational modifications. This approach identified a distinct set of repurposable drugs, including doxazosin, terazosin, and alfuzosin, that share a common α_1_-adrenergic scaffold and inhibit the interaction between p38 and a docking groove–targeting MK2 peptide. We also evaluated their potential for brain permeability and CNS targeting by calculating the CNS multiparameter optimization (MPO) score^67^ and the Balanced Permeability Index (BPI),^68^ which has been shown to outperform the CNS MPO score in predicting permeability and efflux properties (Table S2). Based on BPI < 1 values for nilotinib, doxazosin, terazosin, and alfuzosin, these compounds are expected to have limited brain permeability. However, the MPO scores for alfuzosin (1.77), terazosin (2.14), and doxazosin (1.27) are notably higher than that of nilotinib (0.33), indicating improved CNS-relevant properties. This is consistent with their reported blood–brain barrier penetration and CNS activity in preclinical models.^69,70^ Together with their established human safety profiles, these drugs represent attractive repurposing candidates for Alzheimer’s disease and provide a promising scaffold for further design and optimization of p38/MK2 PPI inhibitors.

In contrast to ATP-competitive inhibitors, our study identifies clinically approved compounds that inhibit the p38/MK2 interface through a noncatalytic mechanism without engaging the ATP-binding cleft. This strategy may minimize off-target effects associated with broader kinase inhibition and supports the development of selective, brain-penetrant anti-inflammatory agents. Collectively, these findings establish a distinct approach for modulating neuroinflammation in AD and provide a platform for therapeutic discovery that circumvents key limitations of conventional kinase inhibitors.

## Conclusions

In summary, our study demonstrates that the p38/MK2 protein–protein interaction interface is a chemically tractable target for therapeutic intervention in neuroinflammatory diseases such as Alzheimer’s disease. Through virtual screening of FDA-approved compounds and the development of a lysate-based TR-FRET assay adapted for ultrahigh-throughput screening, we identified several clinically used agents, including nilotinib and a series of α_1_-adrenergic antagonists, that inhibit the p38/MK2 complex. These compounds interfere with MK2 binding at the p38 docking groove, supporting a non-ATP-competitive mechanism of action. Functional studies in human microglial cells showed that these agents reduce p38/MK2 complex formation and suppress proinflammatory cytokine expression. Structure–activity relationship analysis of nilotinib analogs revealed key pharmacophores required for activity and provided preliminary direction for scaffold optimization. Although additional studies are needed to confirm the compound binding mode, selectivity, and efficacy in vivo, the integration of structure-guided modeling, TR-FRET-based screening, and cellular validation offers a robust framework for discovering PPI-directed therapeutics. Together, this work advances a distinct strategy for repurposing and designing small-molecule inhibitors that modulate inflammatory signaling through selective engagement of the p38/MK2 interface.

## Experimental section

### Molecular Modeling and Docking

All computational studies were conducted using Schrödinger software (release 2025-2). The following p38 crystal structures were used for the docking studies: PDB ID: 6TCA, 2ONL, and 1LEW. The virtual screening was done using the 6TCA structure. While the 1LEW structure represents the mouse p38, the human and mouse p38α isoforms are nearly identical, with the differences in two residues (Leu48His, Thr263Ala) outside the docking groove. The protein structures were prepared using the Protein Preparation Workflow and the OPLS4 force field. The standard precision (SP) mode of the Schrödinger Glide software was used to perform docking-based virtual screening. Compounds were prepared using the Schrödinger LigPrep program. The binding free energy (Δ*G*_bind_) values were calculated using the MM-GBSA method. Molecular dynamics (MD) simulations were performed using the Desmond package. The p38-nilotinib complex was embedded in an orthorhombic box with a 10 Å buffer of TIP3P water molecules, and counterions were added to neutralize the net charge. Default Desmond relaxation protocols were applied, including restrained minimization and short NVT/NPT equilibration. Production simulations were run for 200 ns under the NPT ensemble at 300 K and 1.01325 bar using the Nose–Hoover thermostat and Martyna–Tobias–Klein barostat. A time step of 1.0 fs was used, and coordinates were recorded every 200 ps. Root-mean-square deviation (RMSD) and interaction analyses were performed using the Simulation Interaction Diagram tool in Maestro. The docking of nilotinib analogs was conducted using the 2ONL-based model of the p38/nilotinib complex obtained after 200 ns MD simulation. The 3D QSAR analysis was performed using the Schrödinger Field-Based QSAR tool. The physicochemical and pharmacological properties of compounds (Table S2) were calculated with Schrödinger QikProp software and ADMETlab 3.0.^71^

### Cell Cultures

All cell lines were obtained from the American Type Culture Collection (ATCC, Rockville, MD, USA). HEK293T (ATCC CRL-3216) and HMC3 (ATCC CRL-3304) were cultured in Dulbecco’s modified Eagle’s medium with 4.5 g/L glucose, l-glutamine, and sodium pyruvate (Corning, catalog. no. 10–013-CV) supplemented with 10% fetal bovine serum and 1% penicillin/streptomycin (CellGro, catalog. no. 30–002-CI). Cells were incubated at 37 °C in humidified conditions with 5% CO2.

### iMGL Differentiation

The human induced pluripotent stem cell (hiPSC) line KOLF2.1J was utilized in this study. hiPSCs were maintained on Matrigel-coated plates in mTeSR medium (STEMCELL Technologies, #85850). For human stem-cell-differentiated microglia (iMGL), hiPSCs were initially directed toward a hematopoietic progenitor cell (HPC) lineage using the STEMdiff Hematopoietic Kit (STEMCELL Technologies, # 05310), following the manufacturer’s protocol. Then, HPCs were subsequently differentiated into microglia using microglia medium supplemented with interleukin-34 (IL-34), transforming growth factor-β (TGF-β), and macrophage colony-stimulating factor (M-CSF), following the protocol described by a previous publication.^72^ Final maturation into iMGLs was induced through the addition of CD200 and CX3CL1. The iMGLs expressed the microglia markers, including IBA1 and TMEM119.

### Transfection and Lysis

Cells were transfected using X-tremeGENE HP (SigmaAldrich, catalog. No. 6366546001) in a ratio of 3 μL to 1 μg DNA following the manufacturer’s instructions. Cells were collected after expression for 48 h. Lysis was accomplished with a 1% NP-40 solution (150 mM NaCl, 10 mM HEPES pH 7.5, 1% Nonident P-40 (Sigma-Aldrich, IGEPAL catalog no. CA-630), 5 mM sodium pyrophosphate, 5 mM NaF, 2 mM sodium orthovanadate, 10 mg/L aprotinin, 10 mg/L leupeptin and 1 mM phenylmethylsulfonyl fluoride) mixed with protease (Sigma-Aldrich, catalog no. 539131) and phosphatase inhibitors (Sigma-Aldrich, catalog no. P5726 and no. P0044) at 4 °C for 30 min. Clear lysate was obtained after a 10 min centrifugation at 14,000 rpm, 4 °C.

### Fluorescence Polarization

16 mg/mL His-p38α was obtained from our research collaborators in HEPES buffer (20 mM HEPES, 200 mM NaCl, 5% Glycerol, 1 mM TLEP, pH 8) and diluted to a 5 μM working solution in reaction buffer (20 mM Tris-HCl pH 7.0, 50 mM NaCl, 0.01% NP-40). The working solution was then serially diluted down 2-fold until 205 nM and added to a round-bottom 384-well plate (Corning, catalog no. 4511) at 10 μL per well. FITC conjugated MK2 370–390 peptide was purchased from GenScript and diluted to 10 nM in reaction buffer before being combined with the His-p38α solutions. The fluorescence polarization signal was measured on a BMG Labtech PHERAstar FSX reader.

### Time-Resolved Fluorescence Resonance Energy Transfer (TR-FRET)

In cell lysate-based assays, VF-p38 and GST-MK2 were cotransfected in HEK293T cells and lysed as described above. TR-FRET was conducted in solid bottom 384-well plates (Corning, catalog no. 3571) accommodating 30 μL reaction mixtures per well. Anti-GST-d2 and antiflag-Terbium­(Tb) conjugated antibodies at 1:500 and 1:1000 dilutions, respectively, were used as a FRET donor and acceptor. The optimal concentrations of cell lysate to generate a high signal/background ratio were independently determined through a titration step in each experiment and diluted using reaction buffer (20 mM Tris-HCl pH 7.0, 50 mM NaCl, 0.01% NP-40). For purified recombinant protein experiments, His-p38α (either in-house preparation or Abcam, catalog no. AB271606) and GST-MK2 (Sino Biological, catalog no. M40–14G-100) were added at a final concentration of 10 nM and 1 μM, respectively, also in the reaction buffer. Anti-His-d2 and anti-GST-Tb conjugated antibodies at the same concentration as in lysate-based assays as protein-bound FRET molecules. Twenty-five μL of cell lysate or purified protein was combined with 2 μL mix of antibodies and 3 μL DMSO or compound. Plates were centrifuged at 1000 rpm for 2 min before room temperature incubation for 2 h. The TR-FRET signal was measured on a BMG Labtech PHERAstar FSX reader. The instrument was set to read at Ex 337 nm, Em1:615 nm, Em2:665 nm; time delay: 50 μs to obtain a FRET signal expressed as a 615 nm/665 nm × 104 ratio.

### Flag-Immunoprecipitation

HEK293T cells were cotransfected with GST-MK2 and VF-p38 isoforms or Venus-Flag as negative control and processed to cell lysate according to the standard transfection and lysis protocol. Lysates were incubated with either compounds or DMSO for 30 min before a 75 min incubation with Anti-Flag M2Magnetic beads (Sigma-Aldrich, catalog no. M8823) at 4 °C for 3 h. Beads were washed three times with 1% NP-40 lysis buffer before elution via boiling in 1X sodium dodecyl sulfate-polyacrylamide gel electrophoresis (SDS-PAGE) sample buffer (BioRad, catalog no. 1610747) for 5 min. Samples were analyzed by Western blotting.

### Co-Immunoprecipitation (Co-IP)

HMC3 cells were lysed with 1% NP-40 lysis buffer, as described above. Clear cell lysate was incubated with equilibrated MK2-Trap Agarose beads (ChromoTek, catalog no. mta-20) or Binding Control Agarose Beads (Chromotek, catalog no. bab-20) and compounds or DMSO at 4 °C for 2 h. Immunoprecipitates were washed three times with 1% NP-40 lysis buffer. Samples were boiled in 1X SDS-PAGE sample buffer (BioRad, catalog no. 1610747) for 5 min before analysis by Western blotting.

### Western Blot

Proteins in each sample were separated by SDS-PAGE at 200 V for 40 min using 12.5% acrylamide gels. Transfer to nitrocellulose membranes was completed using the Trans-Blot Turbo Transfer System (BioRad) in TBT buffer (BioRad, catalog no. 10026938). Thirty min incubation in 5% nonfat dry milk (BioRad, catalog no. 170–6404) in TBST buffer (20 mM Tris-base, 150 mM NaCl, and 0.05% Tween 20) at room temperature was used to block membranes. p38α MAPK mouse mAb (Cell Signaling Technology, catalog no. 9217) was used to blot the membrane at 4 °C overnight. Membranes were washed three times with TBST buffer before incubation with Peroxidase Conjugated Goat Anti-Mouse IgG (Jackson Immunoresearch, catalog no. 115–035–003) at room temperature for 1 h. The wash step was repeated, and the blot was imaged with a ChemiDoc imaging system (BioRad). The SuperSignal West Pico PLUS Chemiluminescent Substrate (Thermo Scientific, catalog no. 34580) was used for developing membranes. Blotting of MK2 was then started with the removal of p38α antibodies using a striping buffer (Thermal Scientific, catalog no. 46430). Antibody incubation and imaging were then repeated with MAPKAPK-2 Rabbit Ab (Cell signaling Technology, catalog no. 3042S) and Peroxidase Conjugated Goat Anti-Rabbit IgG (Jackson Immunoresearch, catalog no. 111–035–003).

### Reverse-Transcription Quantitative Polymerase Chain Reaction (RT-qPCR)

HMC3 cells were treated with 100 ng/mL *E. coli* LPS (Sigma-Aldrich, catalog no. L4005) for 4 h in DMEM media. For compound testing, cells were additionally treated with 5 μM compound in 0.1% DMSO for 1 h prior to LPS stimulation. For iMGL-based experiments, iMGLs were pretreated with 5 μM compound in 0.1% DMSO for 1 h prior to inflammatory stimulation. Following pretreatment, iMGLs were challenged with 100 ng/mL LPS and 20 ng/mL interferon-γ (IFN-γ) for 24 h to induce an inflammatory response. At the end of the stimulation, cell lysates were collected for qPCR analysis of TNF-α receptor gene expression. Total RNA isolation from cell samples was conducted using the RNeasy Plus kit (Qiagen, catalog no. 74134) according to manufacturer instructions and treated with DNase I (Invitrogen, catalog no. 18068–015). Extracted RNA was quantified using the Nanodrop One Spectrophotometer (Thermo Scientific) and then reverse-transcribed into cDNA in equal quantity (<1000 ng/reaction) with the SuperScript III System (Invitrogen, catalog no. 18080–051) following manufacturer directions. cDNA sample was treated with RNase H (Invitrogen, catalog no. 18021014) and diluted to 4 ng/μL equivalent of the starting total RNA.

qPCR was performed in 96-well PCR plates (BioRad, catalog no. HSP9601) using the Realplex4Mastercycler (Eppendorf). Each reaction well combines 5 μL cDNA sample, 10 μL 2X SYBR Green Supermix (BioRad, catalog no. 1725271), 1 μL 5 μM primer mix for each target gene, and nuclease-free water up to 20 μL. All target genes, TNFα, IL-6, and IL-1β, were optimized for specificity at >10,000 signal/background ratio and 2 ± 0.05 amplification efficiency. The primer sequences used were: TNFα forward CTCTTCTGCCTGCTGCACTTTG, reverse ATGGGCTACAGGCTTGTCACTC; IL-6 forward AGACAGCCACTCACCTCTTCAG, reverse TTCTGCCAGTGCCTCTTTGCTG; IL-1β forward CCACAGACCTTCCAGGAGAATG, reverse GTGCAGTTCAGTGATCGTACAGG. Each gene was expression-normalized against the housekeeping gene GAPDH with the primer sequences: forward TGCACCACCAACTGCTTAGC, reverse GGCATGGACTGTGGTCATGAG. Primers were obtained from Integrated DNA Technologies in lyophilized form. The cycler program was set to denaturation at 95 °C for 15 s, followed by annealing and extension at 60 °C for 60 s, repeated for 40 PCR cycles. Melting curve analyses were performed for each sample to ensure primer specificity. The experiment was performed in biological quadruplicates and technical quadruplicates. Ct data was analyzed using the 2^–ΔΔCT^ method and expressed in relative mRNA fold over DMSO-treated control.

### TR-FRET Ultra-High-Throughput Screening (uHTS)

The TR-FRET assay was conducted in a 1536-well plate (Corning, catalog no. 3724) with HEK293T cell lysate transfected with VF-p38α and GST-MK2. Cell-lysate concentration was optimized with a titration experiment. Anti-Flag-Tb and anti-GST-d2 antibodies (Revvity, catalog no. 61FGBTLB) were used to couple p38α and MK2, respectively. A multiple-drop Combi dispenser (Thermo Scientific) combined reaction mixtures in quintuplet replicates. The TR-FRET signals were measured using a BMG Labtech PHERAstar FSX plate reader and normalized to the percent of DMSO control.

### Protein Expression and Purification

The gene for the human full-length p38 (MAPK14, Ensembl ID: ENSG00000112062, UniProt ID: Q16539–1) was cloned into a pMCSG7 vector and transformed into *Escherichia coli* strain BL21­(DE3). The *E. coli* BL21­(DE3) cells were grown in Luria Broth (LB) medium at 37 °C to an optical density at 600 nm (OD600) of 0.6–0.8, cooled to 18 °C, and induced overnight with the addition of 0.2 mM isopropyl β-d-1-thiogalactopyranoside (IPTG). Cells from a 3 L culture were resuspended in a buffer comprised of 50 mM Tris pH 8.0, 200 mM NaCl, 1 mM TCEP, and 5% glycerol (equilibration buffer). The resuspended cells were lysed through sonication. The lysate was centrifuged for 20 min at 70,000*g* at 4 °C and the clarified supernatant was passed through a gravity column containing 2 mL Ni–NTA resin pre-equilibrated with equilibration buffer. The resin was then washed with 25 column volumes of equilibration buffer supplemented with 40 mM imidazole, and the protein was eluted with equilibration buffer supplemented with 200 mM imidazole. The eluted protein was further purified by S200 size-exclusion chromatography in equilibration buffer. The purity of the enzyme was verified by sodium dodecyl sulfate-polyacrylamide gel electrophoresis (SDS–PAGE) with Coomassie staining, aliquots flash frozen in liquid nitrogen, and stored at −80 °C.

### Chemical Compounds

All compounds used in this study were purchased from commercial vendors. According to the suppliers, each compound was ≥95% pure, as determined by LC-MS, NMR, or HPLC analysis. Nilotinib was purchased from CombiBlocks (catalog no. QN-4879) and Cayman (catalog no. 10010422). Doxazosin, terazosin, and alfuzosin were purchased from Cayman (catalog no: 18633, 20216, and 13648, respectively). Nilotinib analogs **2–10** were purchased from Enamine (catalog no: **2** - Z3953032891, **3** - Z5228135652, **4** - Z9365590490, **5** - Z9365590489, **6** - Z5302412263, **7** - Z4190288012, **8** - Z5266186646, **9** - Z7579981775, **10** - Z7722392055).

## Supplementary Material













## Notes

Supplementary Figures S1–S4 (PDF)
Supplementary Table S1 with the FDA-approved compounds used in virtual screening, including molecular formula strings and calculated MM-GBSA Δ*G*_bind_ values (CSV)
Table S2 with the compounds from the Emory Enriched Library (EEL) used in the high-throughput screening along with their molecular formula strings determined percent of control values and calculated pharmacological and physicochemical properties (CSV)
PDB file with a model of carvedilol docked to p38, corresponding to Figure 2B,C (PDB)
PDB file with a model of the p38/nilotinib complex obtained after 200 ns MD simulation, corresponding to Figure 2E,F, and Figure 5 (PDB)
Analytical spectra and quality control data demonstrating the identity and purity of nilotinib, nilotinib analogs 2–10, doxazosin, terazosin, and alfuzosin (PDF)

## Abbreviations Used

AD: Alzheimer’s disease
ARE: AU-rich element
Aβ: β-amyloid
BBB: blood–brain barrier
CNS: central nervous system
CSF: cerebrospinal fluid
DMSO: dimethyl sulfoxide
DScore: druggability score
EC_50_: half-maximal effective concentration
EEL: Emory Enriched Library
FITC: fluorescein isothiocyanate
FOC: fold-overcontrol
FP: fluorescence polarization
FDA: U.S. Food and Drug Administration
GST: glutathione S-transferase
HTS: high-throughput screening
IC_50_: half-maximal inhibitory concentration
IL-1β: interleukin-1 β
IL-6: interleukin-6
IP: immunoprecipitation
LPS: lipopolysaccharide
MAPK: mitogen-activated protein kinase
MD: molecular dynamics
MM-GBSA: molecular mechanics with generalized Born and surface area solvation
MK2: MAPK-activated protein kinase 2, MAPKAPK2
NF-κB: nuclear factor kappa B
NPT: constant number of particles, pressure, and temperature ensemble (isothermal–isobaric ensemble)
PPI: protein–protein interaction
qRT-PCR: quantitative reverse transcription polymerase chain reaction
QSAR: quantitative structure–activity relationship
RMSD: root-mean-square deviation
S/B: signal-to-background ratio
SAR: structure–activity relationship
SD: standard deviation
TSA: thermal shift assay
TR-FRET: time-resolved fluorescence resonance energy transfer
TTP: tristetraprolin
uHTS: ultrahigh-throughput screening
VF: Venus–Flag tag
Δ*G*_bind_: binding free energy
Δ*T*_max_: change in melting temperature
