# Supplementary material for: Non-Catalytic Inhibitors of the p38/MK2 Interface: Repurposing Approved Drugs to Target Neuroinflammation in Alzheimer’s Disease
Source: J Med Chem. 2025 Dec 5;68(24):25866–80. doi: 10.1021/acs.jmedchem.5c01425 (PMC12751014; doi:10.1021/acs.jmedchem.5c01425)
Supplement: Supplementary file 1 [file jm5c01425_si_001.pdf]

Supporting Information

Non-Catalytic Inhibitors of the p38/MK2 Interface: Repurposing Approved Drugs to Target Neuroinflammation in Alzheimer's Disease

Maylynn Hu[1†], Andrew Li[1†], Payton Fleming[1†], Julia Gralla[1], Kristos Negrón Terón[1], Ying Zhou[2], Eric J. Miller[1,3], Tyler S. Beyett[1,3], Zhexing Wen[2,4], Yuhong Du[1,3,5], Haian Fu[1,3,5,6], Andrey A. Ivanov[1,3,5,*]

[1]Department of Pharmacology and Chemical Biology, Emory University School of Medicine, Emory University, Atlanta, GA 30322, USA. [2]Department of Psychiatry & Behavioral Sciences Emory University School of Medicine, Atlanta, GA 30322, USA. [3]Winship Cancer Institute, Emory University, Atlanta, GA 30322, USA. [4]Departments of Cell Biology, Neurology, and Human Genetics, Emory University School of Medicine, Atlanta, GA 30322, USA. [5]Emory Chemical Biology Discovery Center, Emory University School of Medicine, Emory University, Atlanta, GA 30322, USA. [6]Department of Hematology & Medical Oncology, Emory University, Atlanta, GA 30322, USA.

*Corresponding author e-mail: andrey.ivanov@emory.edu

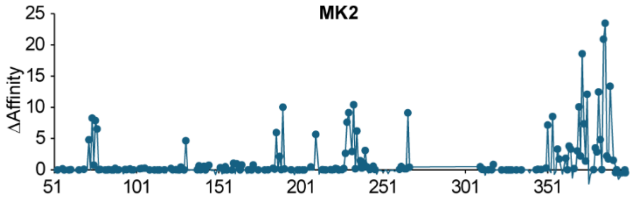

Figure S1. Computational alanine scanning of the p38α–MK2 interface identifies key residues that contribute to complex stability. Systematic alanine substitution across the MK2 surface reveals three discrete regions (residues 72–79, 226–247, and 345–400) where mutations markedly reduce predicted binding affinity for p38α, suggesting these segments form critical interaction hotspots.

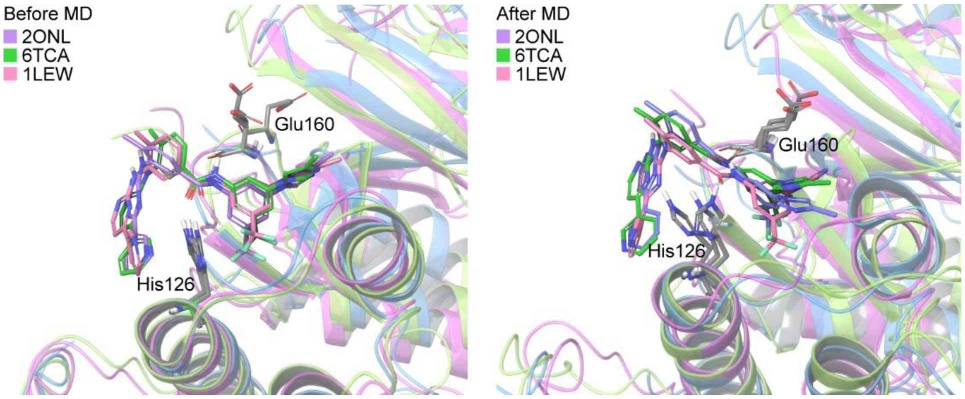

Figure S2. The superimposition of nilotinib binding poses at the p38 docking groove of three different p38 crystal structures (PDB ID: 2ONL, 6TCA, and 1LEW) obtained with molecular docking (left) and after 200 ns MD simulations (right).

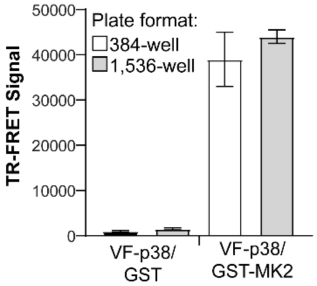

Figure S3. TR-FRET assay performance in high-throughput 384-well and ultra-high-throughput 1,536-well screening formats. The p38/MK2 PPI TR-FRET assay was successfully miniaturized from a 384-well to 1,536-well screening format without loss of signal quality. Assay performance remained robust across both configurations, with signal-to-background (S/B) ratios exceeding 20, supporting its suitability for large-scale compound screening.

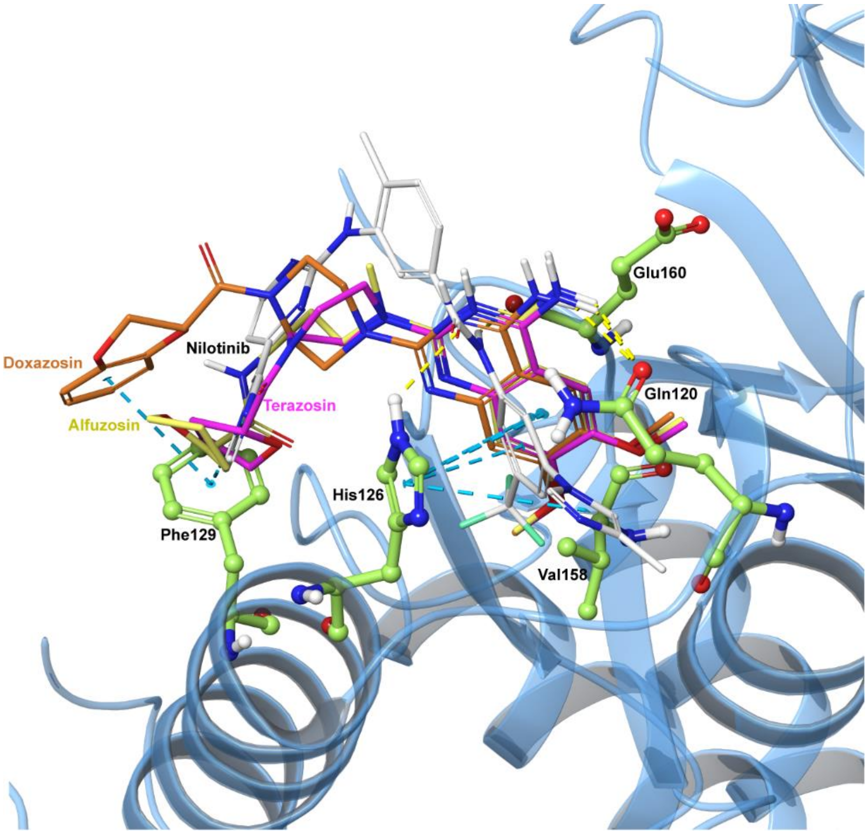

Figure S4. Superimposed docking poses of Nilotinib (white), Alfuzosin (yellow), Doxazosin (orange), and Terazosin (purple) within the p38 docking groove. All compounds adopted a similar binding orientation. Alfuzosin, doxazosin, and terazosin were consistently engaged in pi-pi stacking with His126 and formed a hydrogen bond with Gln120. Their methoxy group was bound to the p38 hydrophobic pocket, similarly to the nilotinib's trifluoromethyl group.
