# Supplementary material for: Non-Catalytic Inhibitors of the p38/MK2 Interface: Repurposing Approved Drugs to Target Neuroinflammation in Alzheimer’s Disease
Source: J Med Chem. 2025 Dec 5;68(24):25866–80. doi: 10.1021/acs.jmedchem.5c01425 (PMC12751014; doi:10.1021/acs.jmedchem.5c01425)
Supplement: Supplementary file 6 [file jm5c01425_si_006.pdf]

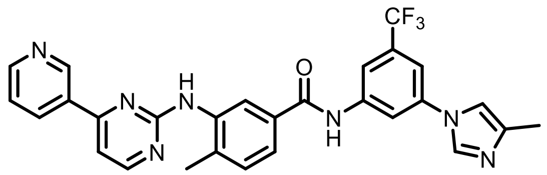

4-Methyl-*N*-(3-(4-methyl-1*H*-imidazol-1-yl)-5-(trifluoromethyl)phenyl)-3-((4-(pyridin-3-yl)pyrimidin-2-yl)amino)benzamide hydrochloride hydrate (nilotinib hydrochloride monohydrate) was purchased from CombiBlocks (Cat. # QN-4879).

Compound purity was determined to be ≥95% based on ^{1}H NMR, which was acquired using a Varian Inova 400 MHz NMR spectrometer in d_6-DMSO (residual solvent chemical shift set to 2.50 ppm). ^{1}H NMR (400 MHz, d_6-DMSO) δ 10.97 (s, 1H), 9.59 (d, *J* = 4.0 Hz, 1H), 9.29 (d, *J* = 4.0 Hz, 1H), 9.21 (s, 1H), 8.70 (dd, *J* = 4.0 Hz, *J* = 1.6 Hz, 1H), 8.64-8.65 (m, 1H), 8.55 (d, *J* = 4.0 Hz, 1H), 8.50 (dt, *J* = 8.0 Hz, *J* = 4.0 Hz, 1H), 8.33-8.35 (m, 2H), 8.00 (m, 1H), 7.91-7.92 (m, 1H), 7.87 (dd, *J* = 8.0 Hz, *J* = 1.6 Hz, 1H), 7.56 (dd, *J* = 8.0 Hz, *J* = 4.0 Hz, 1H), 7.50 (d, *J* = 4.0 Hz, 1H), 7.45 (d, *J* = 8.0 Hz, 1H), 2.35 (br s, 6H).

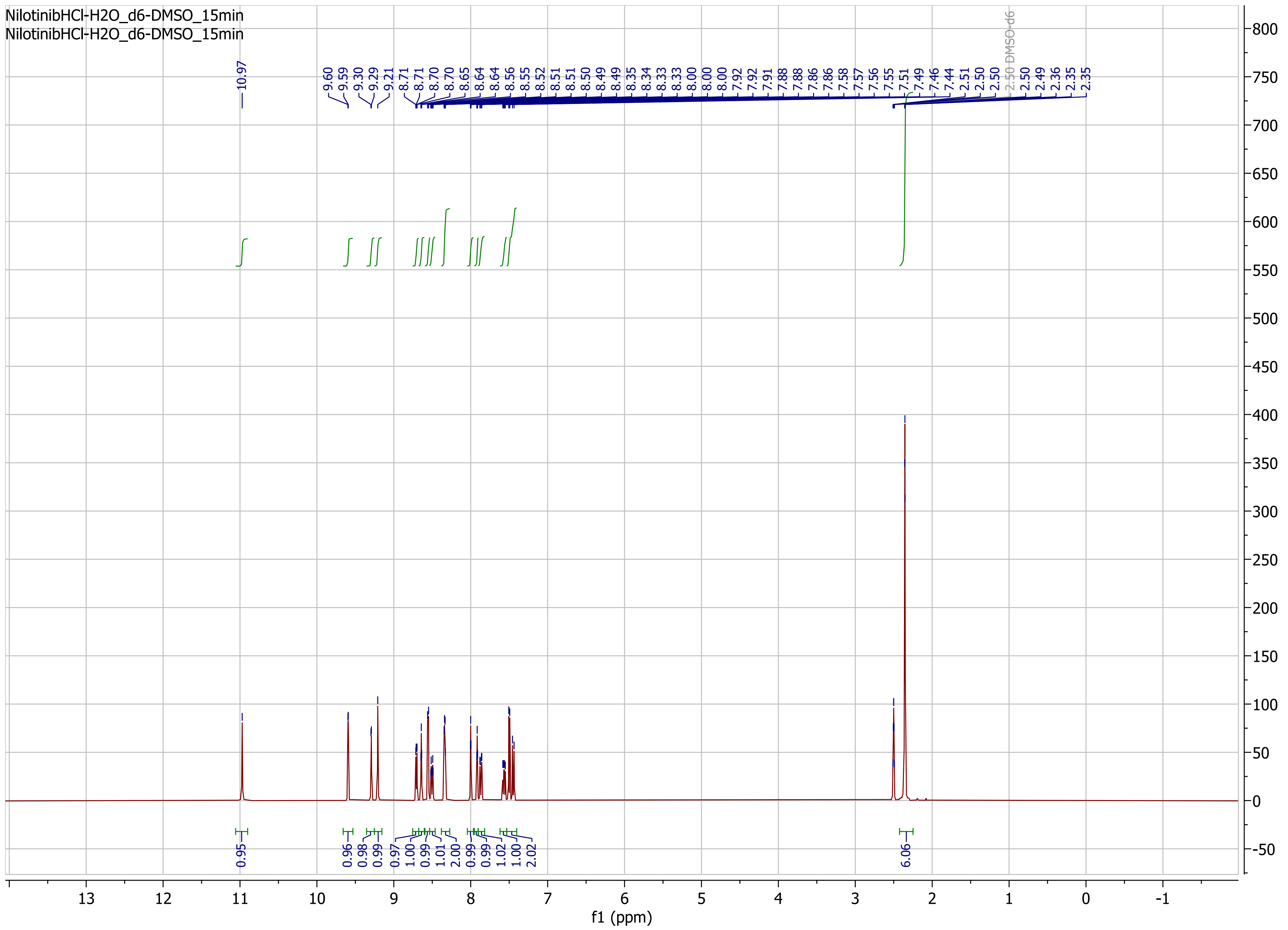
NilotinibHCl-H2O_d6-DMSO_15min
NilotinibHCl-H2O_d6-DMSO_15min
10.97
9.60
9.59
9.30
9.29
9.21
8.71
8.71
8.70
8.70
8.65
8.64
8.64
8.56
8.55
8.52
8.51
8.51
8.50
8.49
8.49
8.35
8.34
8.33
8.33
8.00
8.00
8.00
7.92
7.92
7.91
7.88
7.88
7.86
7.86
7.58
7.57
7.56
7.55
7.51
7.49
7.46
7.44
2.51
2.50
2.50
2.50 DMSO-d6
2.50
2.49
2.36
2.35
2.35
0.95
0.96
0.98
0.99
0.97
1.00
0.99
1.01
2.00
0.99
0.99
1.02
1.00
2.02
6.06
f1 (ppm)

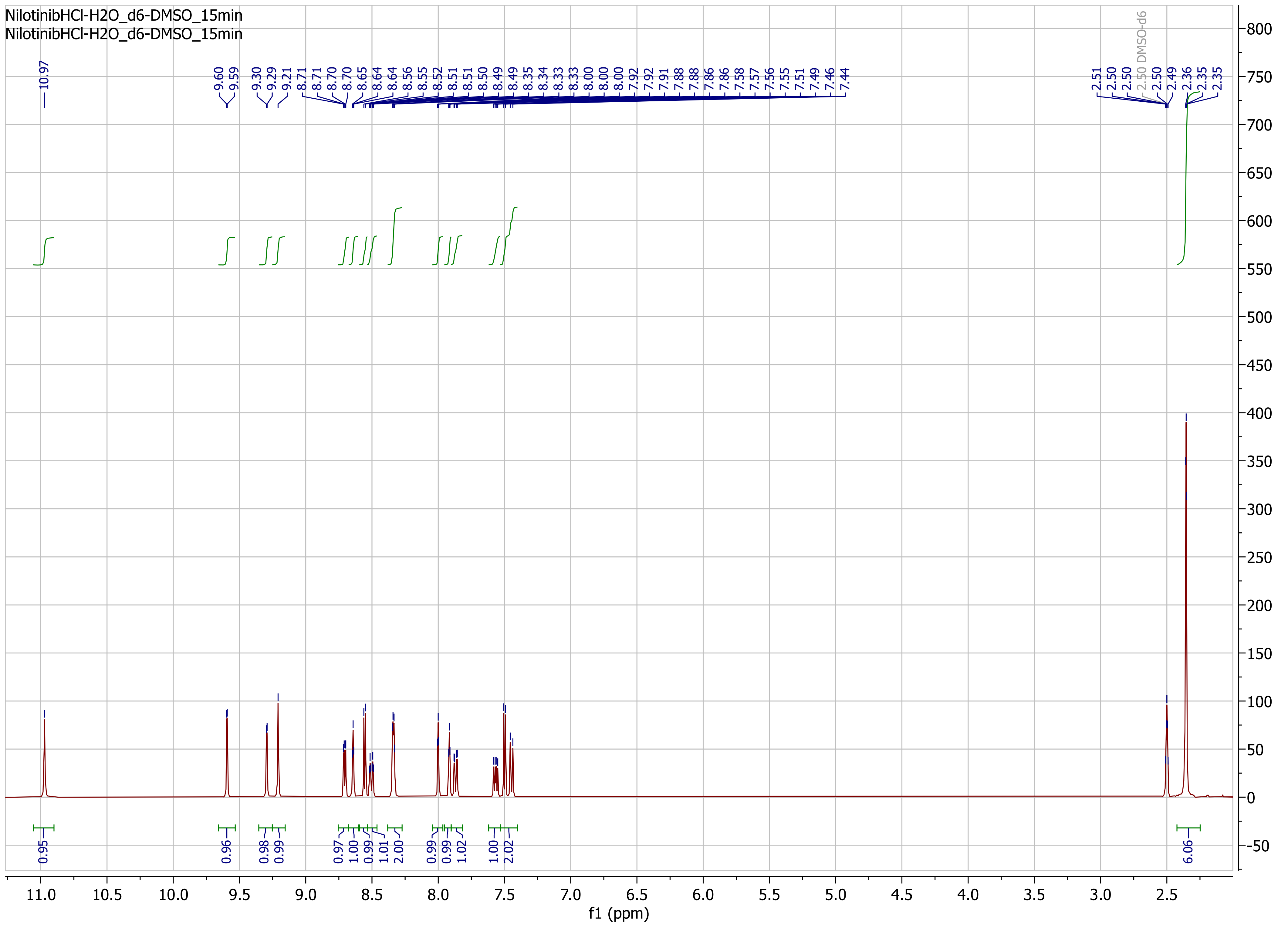
NilotinibHCl-H2O_d6-DMSO_15min
NilotinibHCl-H2O_d6-DMSO_15min
10.97
9.60 9.59 9.30 9.29 9.21 8.71 8.71 8.70 8.70 8.65 8.64 8.64 8.56 8.55 8.52 8.51 8.51 8.50 8.49 8.49 8.35 8.34 8.33 8.33 8.00 8.00 8.00 7.92 7.92 7.91 7.88 7.88 7.86 7.86 7.58 7.57 7.56 7.55 7.51 7.49 7.46 7.44
2.51 2.50 2.50 2.50 DMSO-d6 2.50 2.49 2.36 2.35 2.35
0.95 0.96 0.98 0.99 0.97 1.00 0.99 1.01 2.00 0.99 0.99 1.02 1.00 2.02 6.06
f1 (ppm)

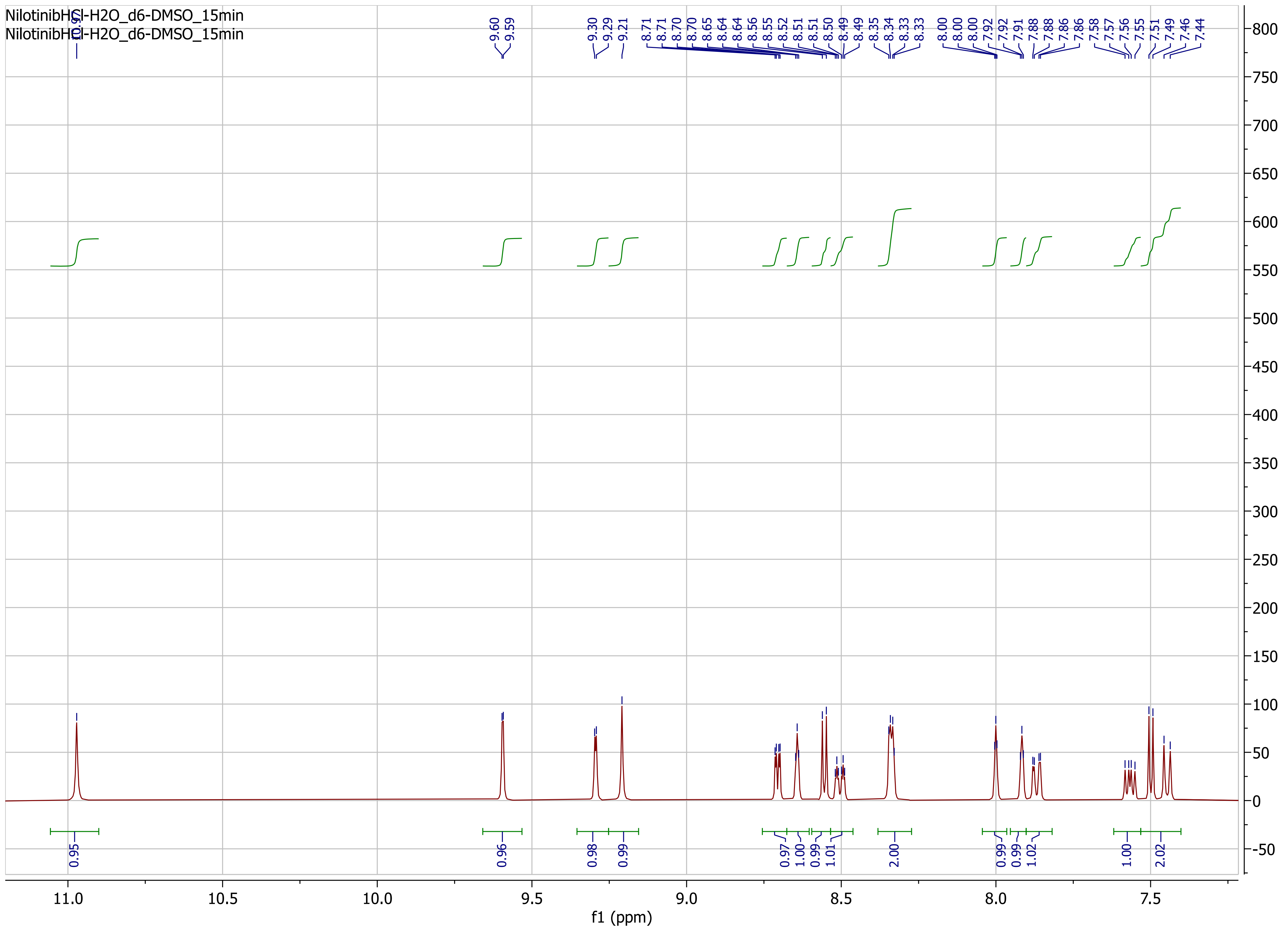

NilotinibHCl-H2O_d6-DMSO_15min
NilotinibHCl-H2O_d6-DMSO_15min
9.60
9.59
9.30
9.29
9.21
8.71
8.71
8.70
8.70
8.65
8.64
8.64
8.56
8.55
8.52
8.51
8.51
8.50
8.49
8.49
8.35
8.34
8.33
8.33
8.00
8.00
8.00
7.92
7.92
7.91
7.88
7.88
7.86
7.86
7.58
7.57
7.56
7.55
7.51
7.49
7.46
7.44
0.95
0.96
0.98
0.99
0.97
1.00
0.99
1.01
2.00
0.99
0.99
1.02
1.00
2.02
f1 (ppm)

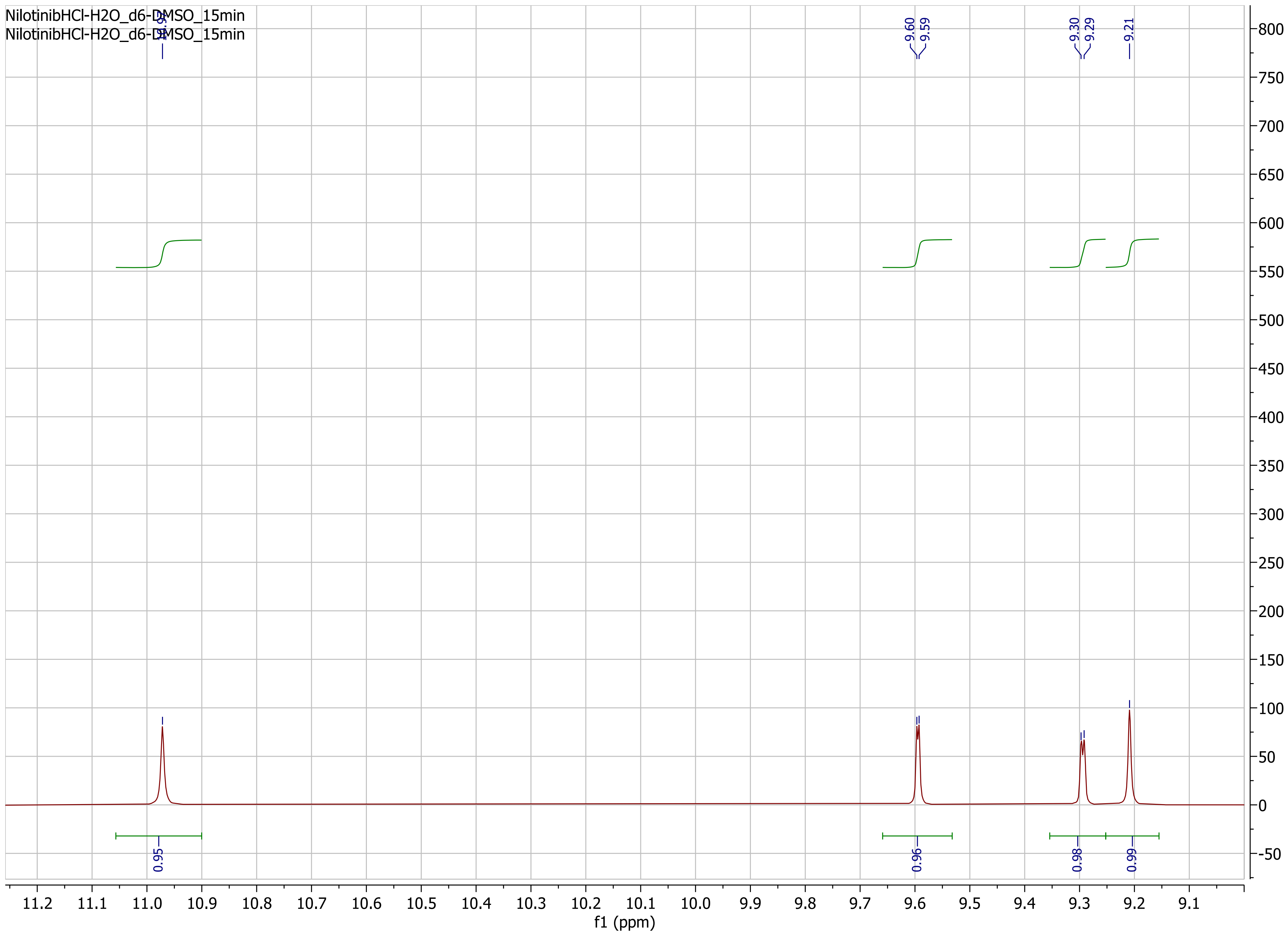

NilotinibHCl-H2O_d6-DMSO_15min
NilotinibHCl-H2O_d6-DMSO_15min
9.60
9.59
9.30
9.29
9.21
0.95
0.96
0.98
0.99
f1 (ppm)

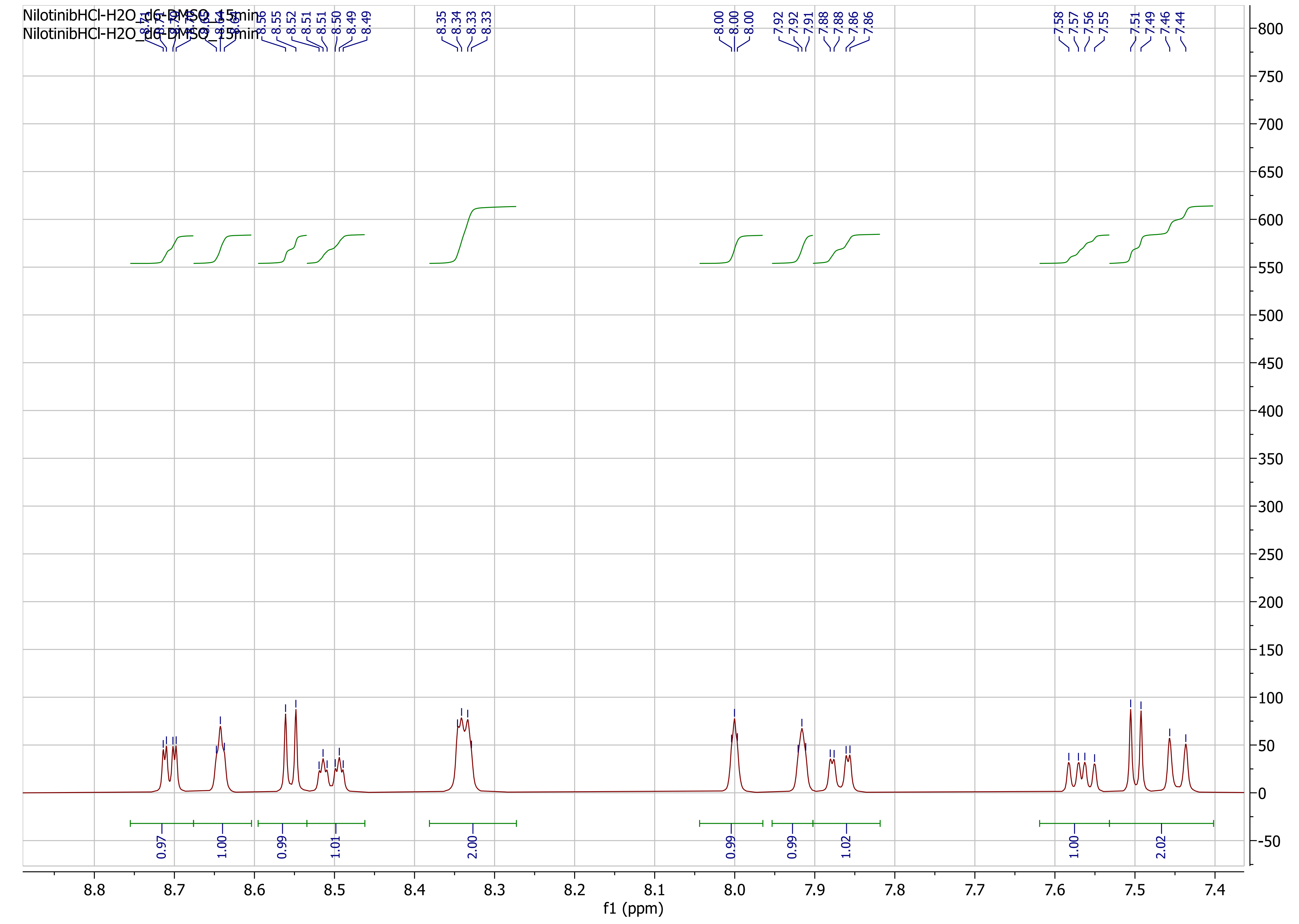

NilotinibHCl-H2O_d6-DMSO_15min
NilotinibHCl-H2O_d6-DMSO_15min
8.56
8.55
8.52
8.51
8.51
8.50
8.49
8.49
8.35
8.34
8.33
8.33
8.00
8.00
8.00
7.92
7.92
7.91
7.88
7.88
7.86
7.86
7.58
7.57
7.56
7.55
7.51
7.49
7.46
7.44
0.97
1.00
0.99
1.01
2.00
0.99
0.99
1.02
1.00
2.02
f1 (ppm)

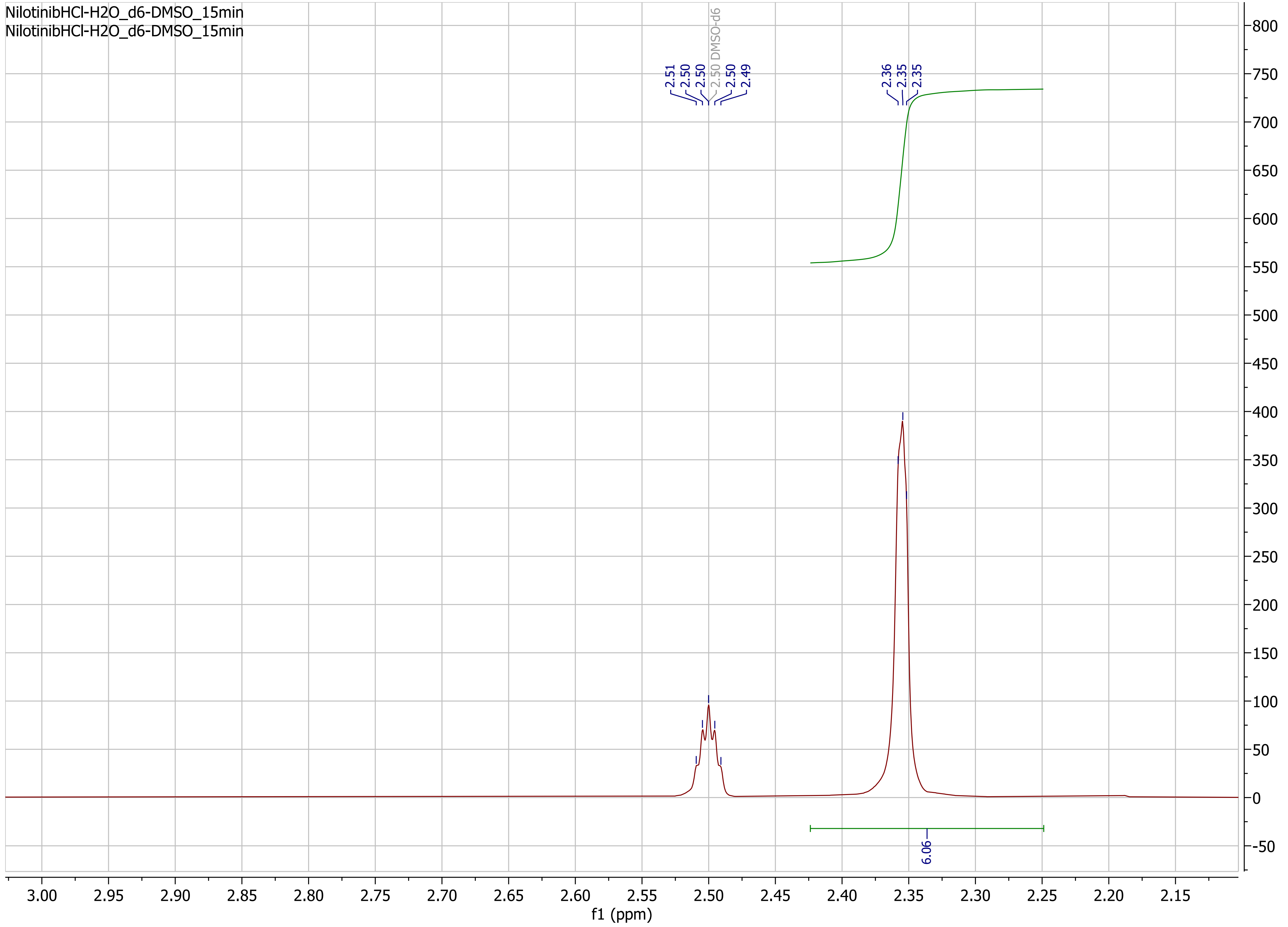

NilotinibHCl-H2O_d6-DMSO_15min
NilotinibHCl-H2O_d6-DMSO_15min
2.51
2.50
2.50
2.50 DMSO-d6
2.50
2.49
2.36
2.35
2.35
6.06
f1 (ppm)

CERTIFICATE of ANALYSIS

Cayman CHEMICAL

Nilotinib

4-methyl-N-[3-(4-methyl-1H-imidazol-1-yl)-5-(trifluoromethyl)phenyl]-3-[[4-(3-pyridinyl)-2-pyrimidinyl]amino]-benzamide

Item No. 10010422 • Batch No. 0553019

Purity Specification: ≥95%
Molecular Formula.: $C_{28}H_{22}F_3N_7O$
CAS Number: 641571-10-0 Formula Weight: 529.5 Expiry date: 06JUN2027

Overview

Tests	Results
HPLC	Purity: 98.2 %
IR	Conforms
Mass spec	MH+: 530.2
Melting Point	230 - 232 ºC
TLC	Purity: 100 %
UV	λ max: 263 nm
NMR	Conforms

Reviewed and approved by: Jennifer LaBrecque

Jen LaBrecque

WARNING
THIS PRODUCT IS FOR RESEARCH USE - NOT FOR HUMAN OR VETERINARY DIAGNOSTIC OR THERAPEUTIC USE. IT IS THE RESPONSIBILITY OF THE PURCHASER TO DETERMINE SUITABILITY FOR OTHER APPLICATIONS.

SAFETY DATA
This material should be considered hazardous until further information becomes available. Do not ingest, inhale, get in eyes, on skin, or on clothing. Wash thoroughly after handling. Before use, the user must review the complete Safety Data Sheet, which has been sent via email to your institution.

WARRANTY AND LIMITATION OF REMEDY
Buyer agrees to purchase the material subject to Cayman's Terms and Conditions. Complete Terms and Conditions including Warranty and Limitation of Liability information can be found on our website.

Copyright Cayman Chemical Company, 10/12/2018

CAYMAN CHEMICAL
1180 EAST ELLSWORTH RD
ANN ARBOR, MI 48108 · USA
PHONE: [800] 364-9897
[734] 971-3335
FAX: [734] 971-3640
CUSTSERV@CAYMANCHEM.COM
WWW.CAYMANCHEM.COM

===

Acq. Operator : SYSTEM
Sample Operator : SYSTEM
Acq. Instrument : HPLC3 Location : Vial 1
Injection Date : 12/15/2020 1:40:41 PM
Inj Volume : Manually
Acq. Method : C:\CHEM32\1\METHODS\GENERIC.M
Last changed : 12/15/2020 1:50:49 PM by SYSTEM
(modified after loading)
Analysis Method : C:\CHEM32\1\METHODS\GENERIC.M
Last changed : 12/14/2020 2:43:02 PM by SYSTEM
Sample Info : Nilotinib; Cat #10010422; LT0553019; Re QC
Gemini; C18; 5µ 4.6x250mm
210 nm; p=143 bar; 1mL/min
75:25 MeOH:NH4OAc (pH 7.0, 10mM)

Additional Info : Peak(s) manually integrated

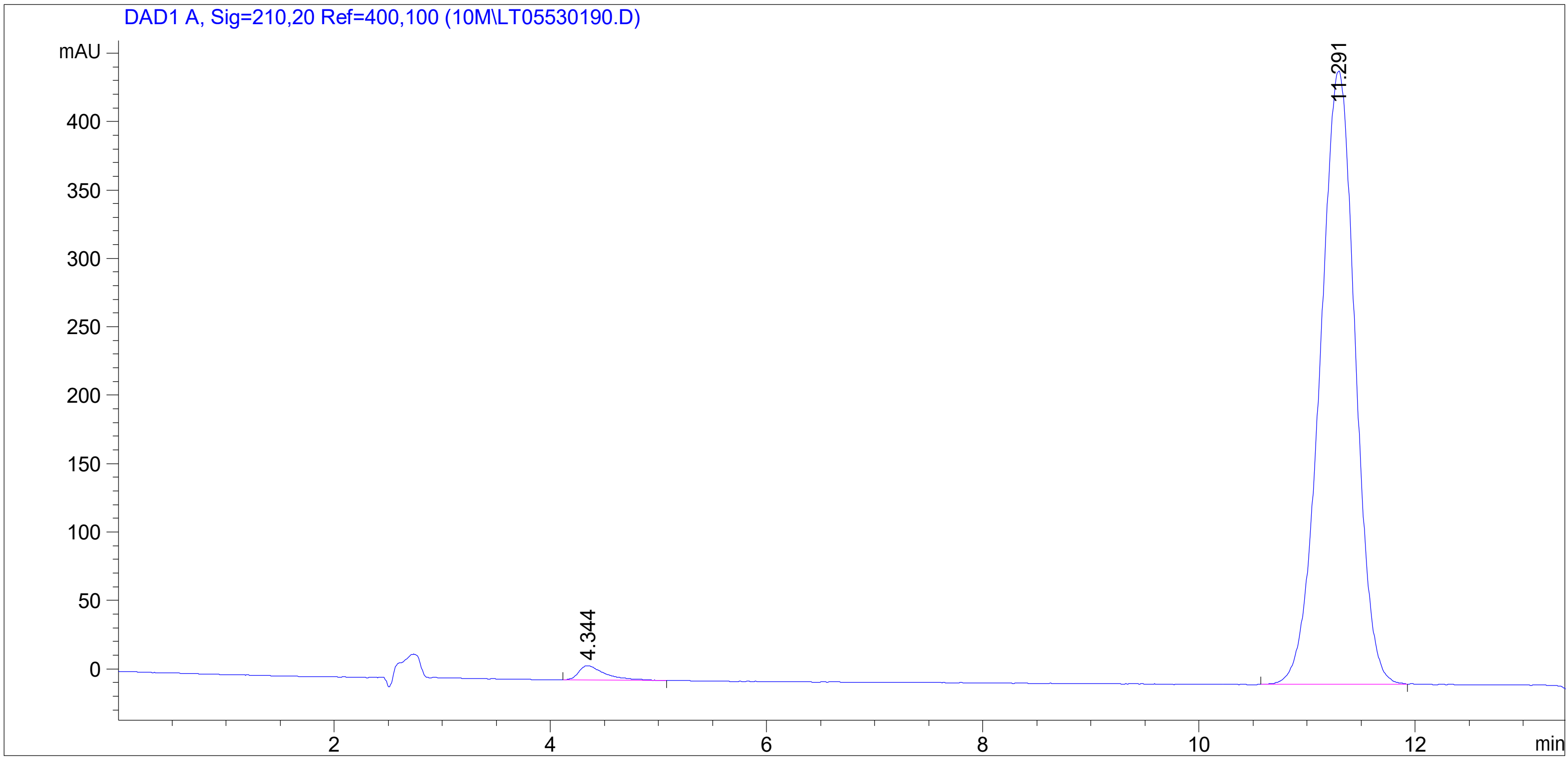

===

Area Percent Report

===

Sorted By : Signal
Multiplier : 1.0000
Dilution : 1.0000
Do not use Multiplier & Dilution Factor with ISTDs

Signal 1: DAD1 A, Sig=210,20 Ref=400,100

Peak #	RetTime [min]	Type	Width [min]	Area [mAU*s]	Height [mAU]	Area %
1	4.344	BB	0.2481	181.34375	10.36493	1.7648
2	11.291	BV	0.3447	1.00943e4	448.31448	98.2352

Totals : 1.02757e4 458.67941

===

*** End of Report ***

PerkinElmer Spectrum IR Version 10.6.1
Thursday, March 14, 2019 10:18 AM

Analyst	Administrator
Date	Thursday, March 14, 2019 10:18 AM

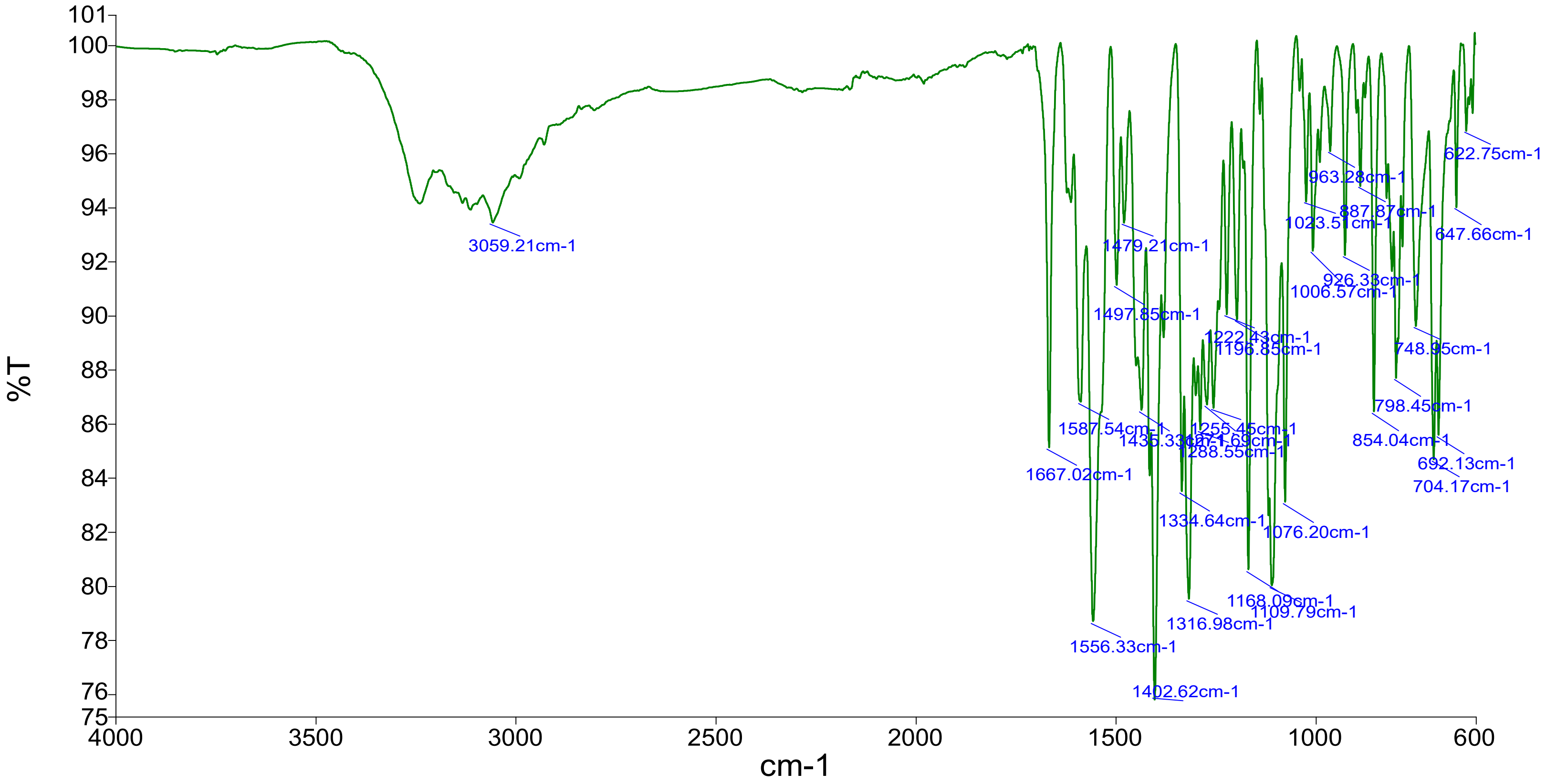

QC35030_1_1 Nilotinib; # 10010422; QC35030; Crystal

C:\MassSpecData\2019\10010422_QC35030 2/26/2019 8:31:11 AM Nilotinib
NUTRALAPCIPOSHIFLO

10010422_QC35030 #36-38 RT: 0.29-0.31 AV: 3 NL: 2.10E5
T: ITMS + c APCI corona Full ms [105.00-1000.00]

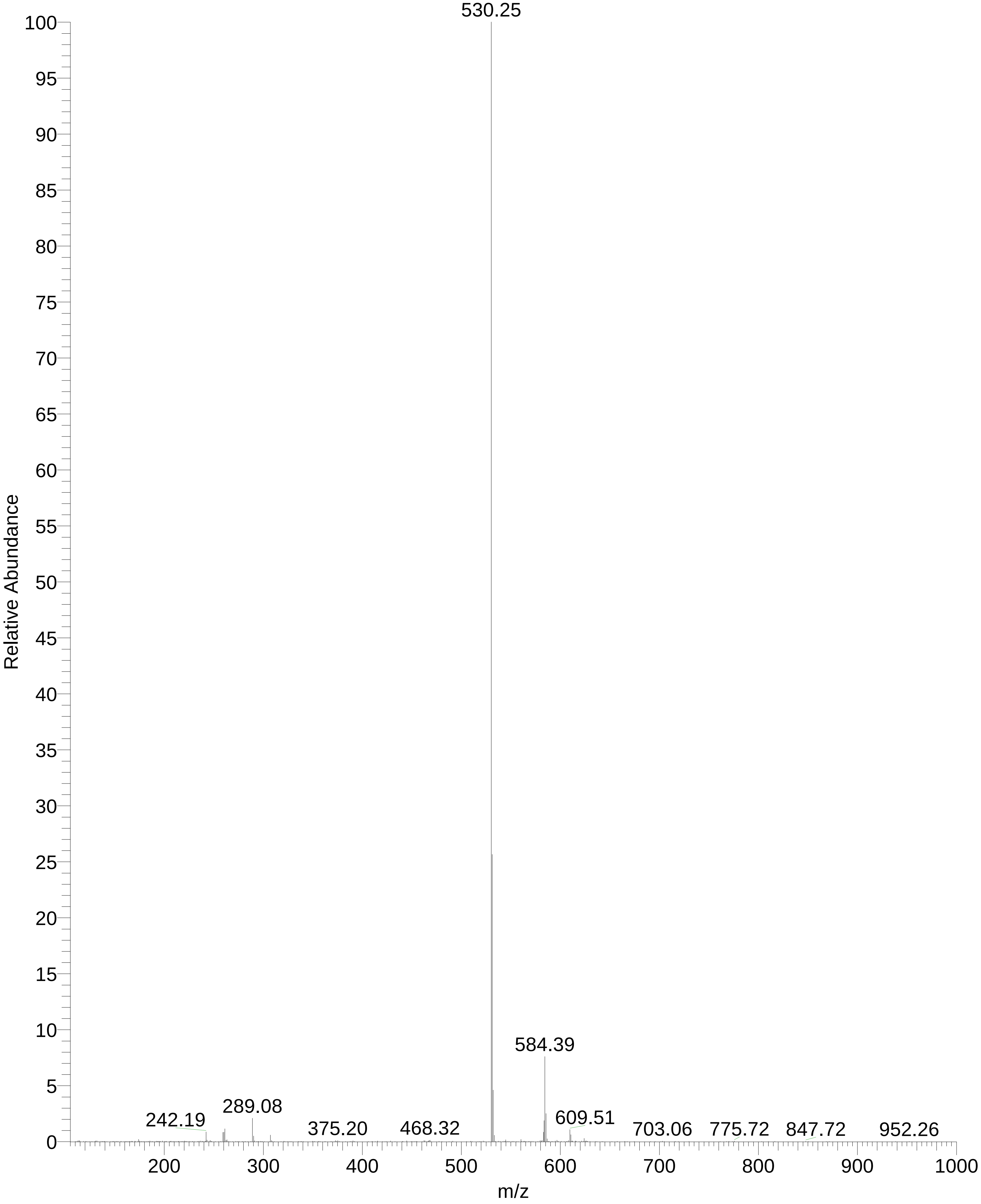

Nilotinib

Item #10010422

Batch #0553019

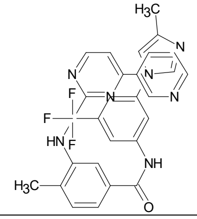

File Name	\\sulfur\private\nmrdata\JEOL_2020\10010422-0553019\10010422-0553019_PROTON_21-Dec-2020-1-1.jdf				
Date	21 Dec 2020 12:05:19	***Nucleus***	1H	***Frequency (MHz)***	399.7822
Solvent	DMSO-d6	***Number of Transients***	16	***Origin***	JEOL ECZ400S Ca
Temperature (degree C)	21.600				

^{1}H NMR (DMSO-d_6, 400 MHz) δ 10.58 (s, 1H), 9.24 (d, 1H, *J*=1.4 Hz), 9.14 (s, 1H), 8.64 (dd, 1H, *J*=1.4, 4.6 Hz), 8.51 (d, 1H, *J*=5.0 Hz), 8.41 (td, 1H, *J*=2.0, 7.9 Hz), 8.28 (d, 2H, *J*=10.3 Hz), 8.16 (d, 1H, *J*=1.4 Hz), 8.12 (s, 1H), 7.73 (dd, 1H, *J*=1.6, 8.0 Hz), 7.68 (s, 1H), 7.4-7.5 (m, 4H), 2.32 (s, 3H), 2.14 (s, 3H)

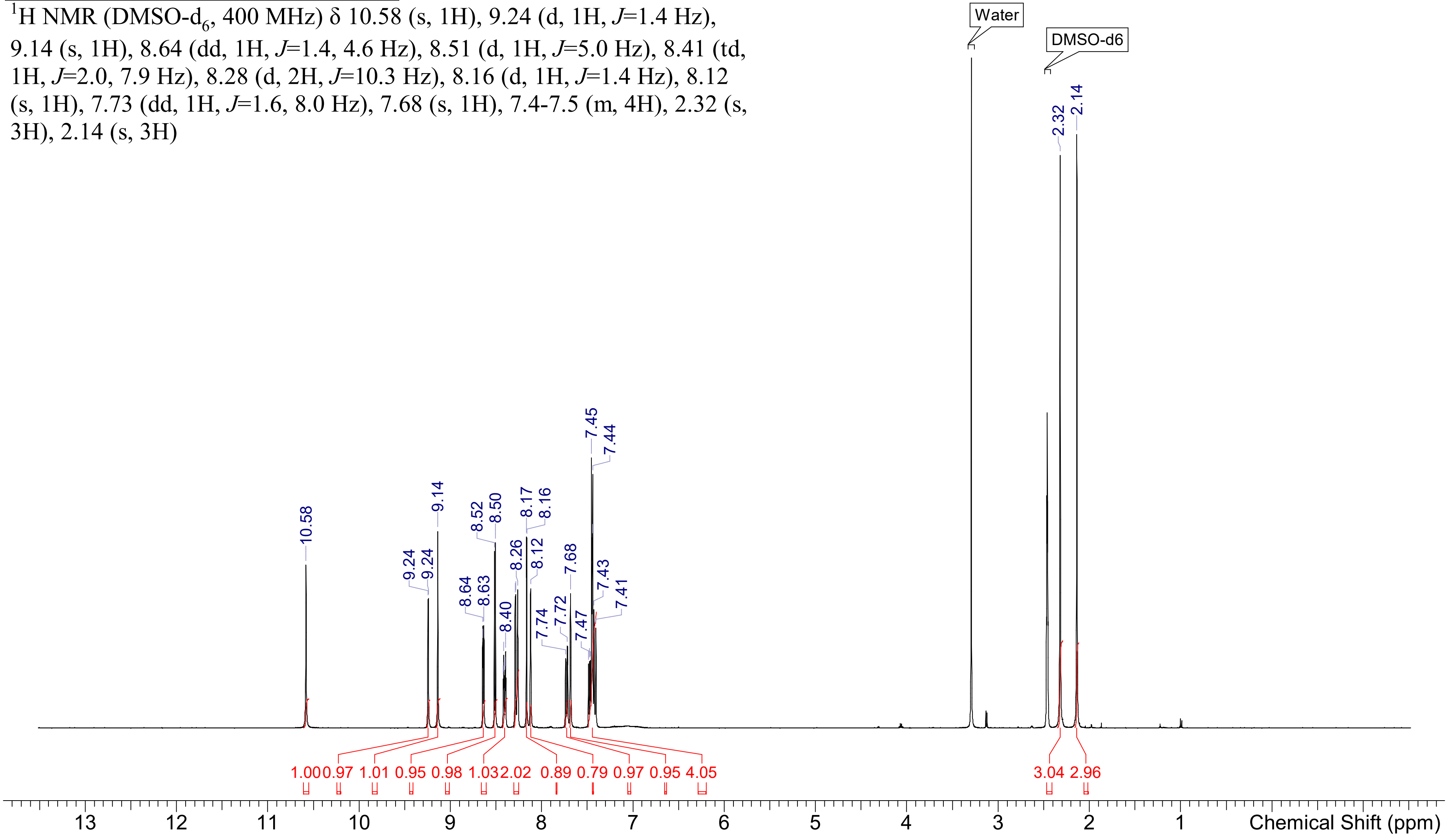

3/14/2019 10:22:42 AM

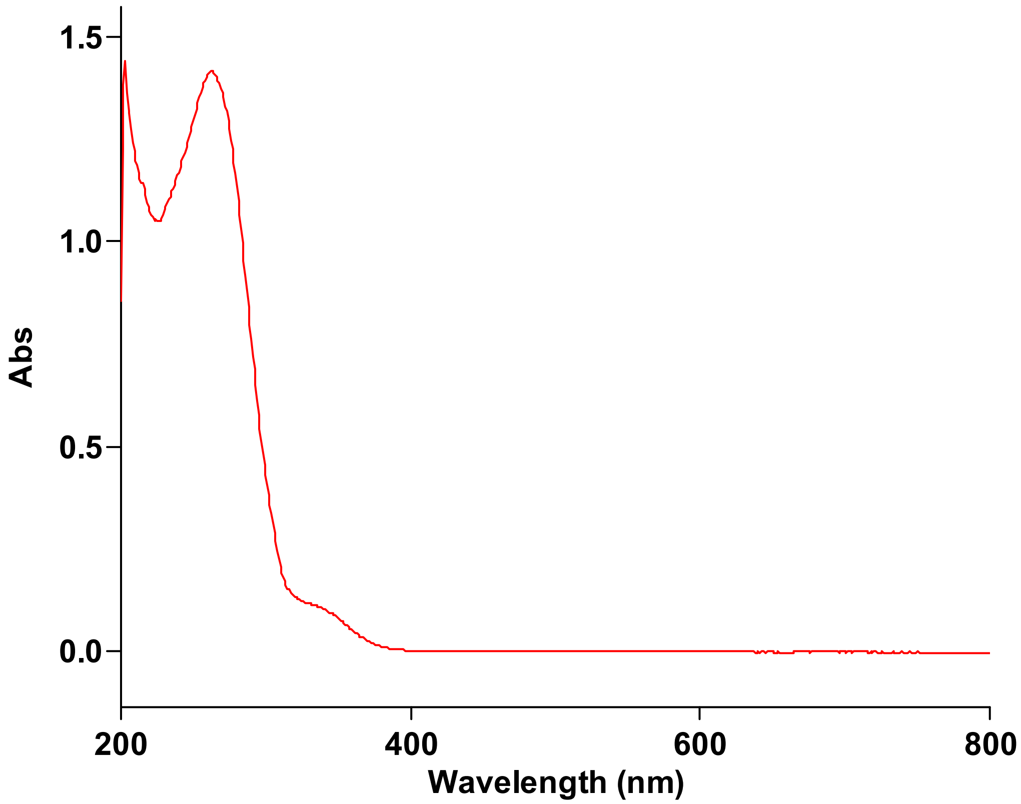

Cayman Chemical Company

Nilotinib
10010422 QC35030
A Solution in Ethanol

Sample Name: **sample17**

Collection Time 3/14/2019 10:22:14 AM

Peak Table	
Peak Style	Peaks
Peak Threshold	0.0100
Range	800.0nm to 200.0nm
Wavelength (nm)	Abs
263.0	1.416
203.0	1.443

CERTIFICATE of ANALYSIS

Cayman CHEMICAL

Alfuzosin (hydrochloride)

N-[3-[(4-amino-6,7-dimethoxy-2-quinazolinyl)methylamino]propyl]tetrahydro-2-furancarboxamide, monohydrochloride

*Item No. 13648 * Batch No. 0420452*

Purity Specification: >98%

Molecular formula: $C_{19}H_{27}N_5O_4 \cdot HCl$

CAS number: **81403-68-1** **Formula weight:** **425.90** **Expiration date:** **29AUG2029**

Overview

Tests	Results
HPLC	Purity: 99.8 %
IR	Conforms
Mass Spec	[M+H]+: 390.5
Melting point	220 - 224 ºC
TLC	Purity: 100 %
UV	λ max: 344.00 nm<br>λ max: 331.00 nm<br>λ max: 246.00 nm

Reviewed and approved by: Erik Guetschow

WARNING
THIS PRODUCT IS FOR RESEARCH USE - NOT FOR HUMAN OR VETERINARY DIAGNOSTIC OR THERAPEUTIC USE. IT IS THE RESPONSIBILITY OF THE PURCHASER TO DETERMINE SUITABILITY FOR OTHER APPLICATIONS.

SAFETY DATA
This material should be considered hazardous until further information becomes available. Do not ingest, inhale, get in eyes, on skin, or on clothing. Wash thoroughly after handling. Before use, the user must review the complete Safety Data Sheet, which has been sent via email to your institution.

WARRANTY AND LIMITATION OF REMEDY
Buyer agrees to purchase the material subject to Cayman's Terms and Conditions. Complete Terms and Conditions including Warranty and Limitation of Liability information can be found on our website.

Copyright Cayman Chemical Company, 10/12/2018

CAYMAN CHEMICAL
1180 EAST ELLSWORTH RD
ANN ARBOR, MI 48108 · USA
PHONE: [800] 364-9897
[734] 971-3335
FAX: [734] 971-3640
CUSTSERV@CAYMANCHEM.COM
WWW.CAYMANCHEM.COM

SAMPLE INFORMATION

Sample Name:	13648-0420452	Project:	Aug_2025
Location:	1:F,2	Sample Set Name:	29_Aug_2025_1
Injection Volume:	0.20 ul	Instrument Method:	Ampgrad
Acquired By:	preplab	Processing Method:	Ampgrad
Instrument ID:	LCMS03_UV	Date Acquired:	8/29/2025 10:42:18 AM EDT
		Date Processed:	8/29/2025 10:52:48 AM EDT

METHOD INFORMATION

Mobile Phase A:	90:10:0.1 H2O:ACN:TFA	Time (min)	%B
Mobile Phase B:	10:90:0.1 H2O:ACN:TFA	0	0
		5	100
Flow Rate:	0.5 mL/min	6	100
Column:	Poroshell SB-C18, 2.1 x 100 mm, 2.7 um	6.1	0
Column S/N:	USCFH10852	9	0

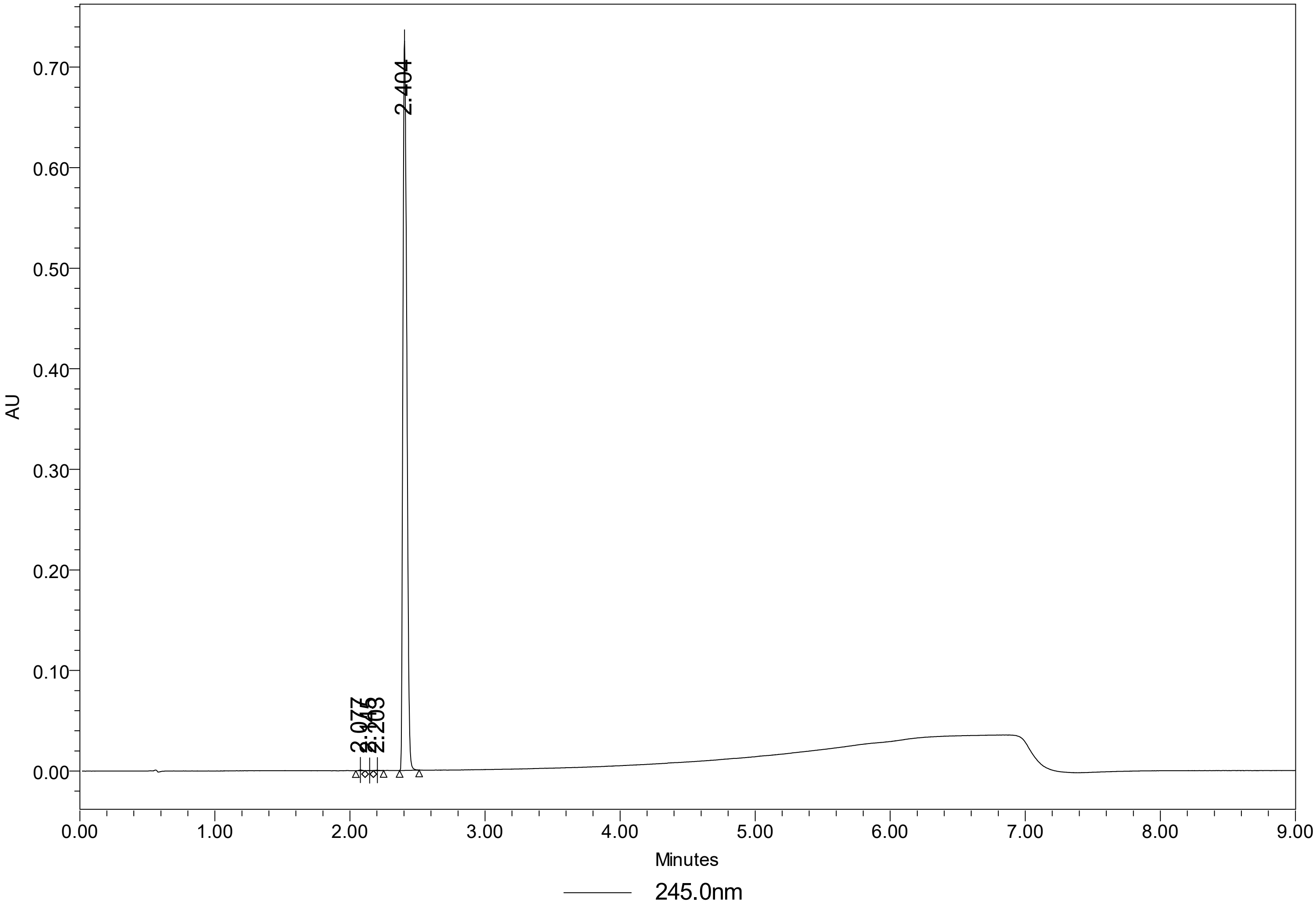

Peak Results

	RT	Int Type	Width (sec)	Area	Height	% Area
1	2.077	bv	4.100	1210	700	0.08
2	2.145	vv	3.700	383	195	0.03
3	2.203	vb	4.550	911	396	0.06
4	2.404	BB	8.699	1452015	725668	99.83

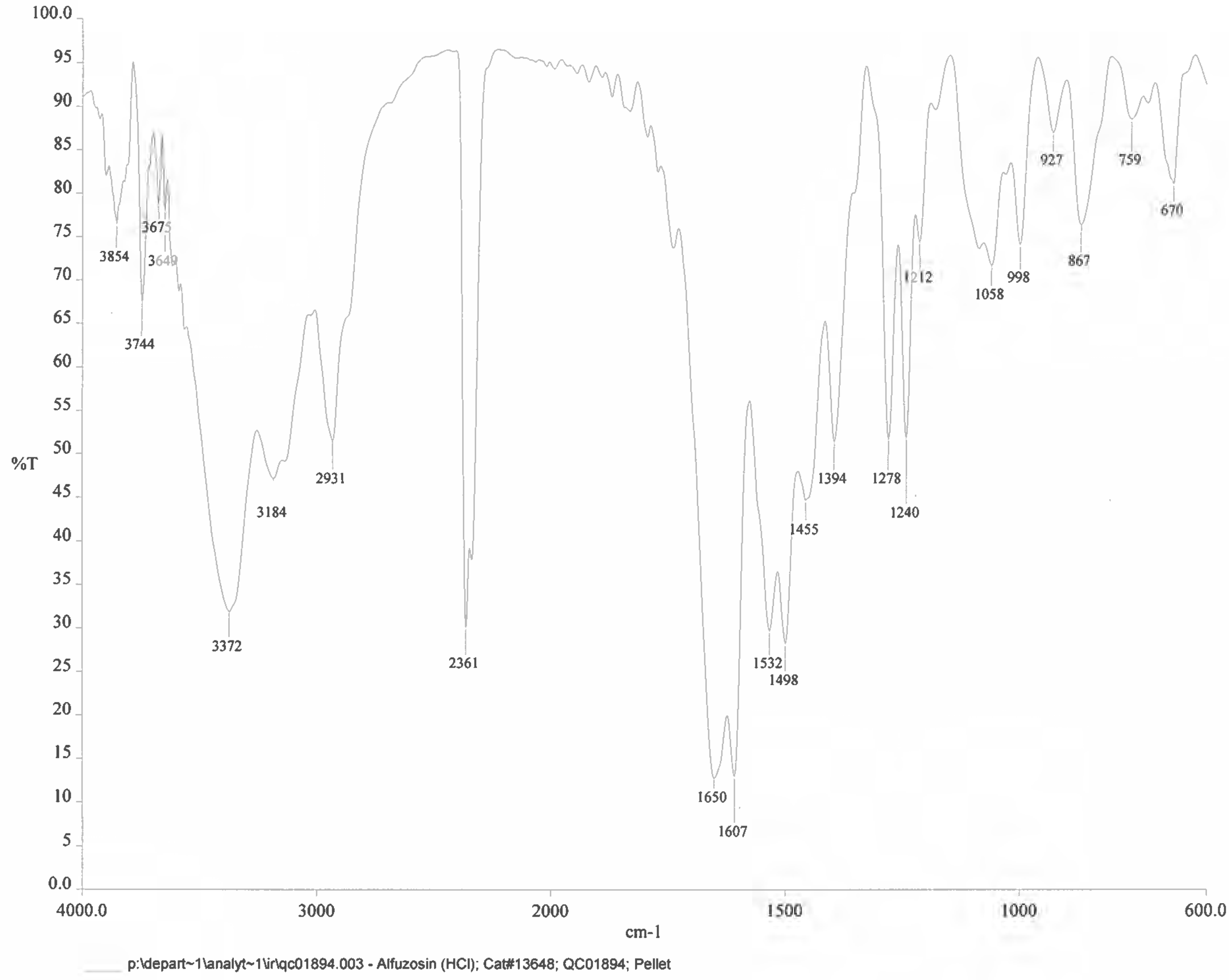
100.0
95
90
85
80
75
70
65
60
55
50
45
40
35
30
25
20
15
10
5
0.0
%T
3854
3744
3675
3649
3372
3184
2931
2361
1650
1607
1532
1498
1455
1394
1278
1240
1212
1058
998
927
867
759
670
4000.0
3000
2000
1500
1000
600.0
cm-1
p:\depart~1\analyt~1\ir\qc01894.003 - Alfuzosin (HCl); Cat#13648; QC01894; Pellet

C:\Xcalibur\...\13648_100521112440 5/21/2010 11:24:40 AM Alfuzosin (HCl)
AMNAESILOWFLOW

13648_100521112440 #28-53 RT: 1.01-1.92 AV: 26 NL: 1.63E6
T: + c ESI Full ms [50.00-800.00]

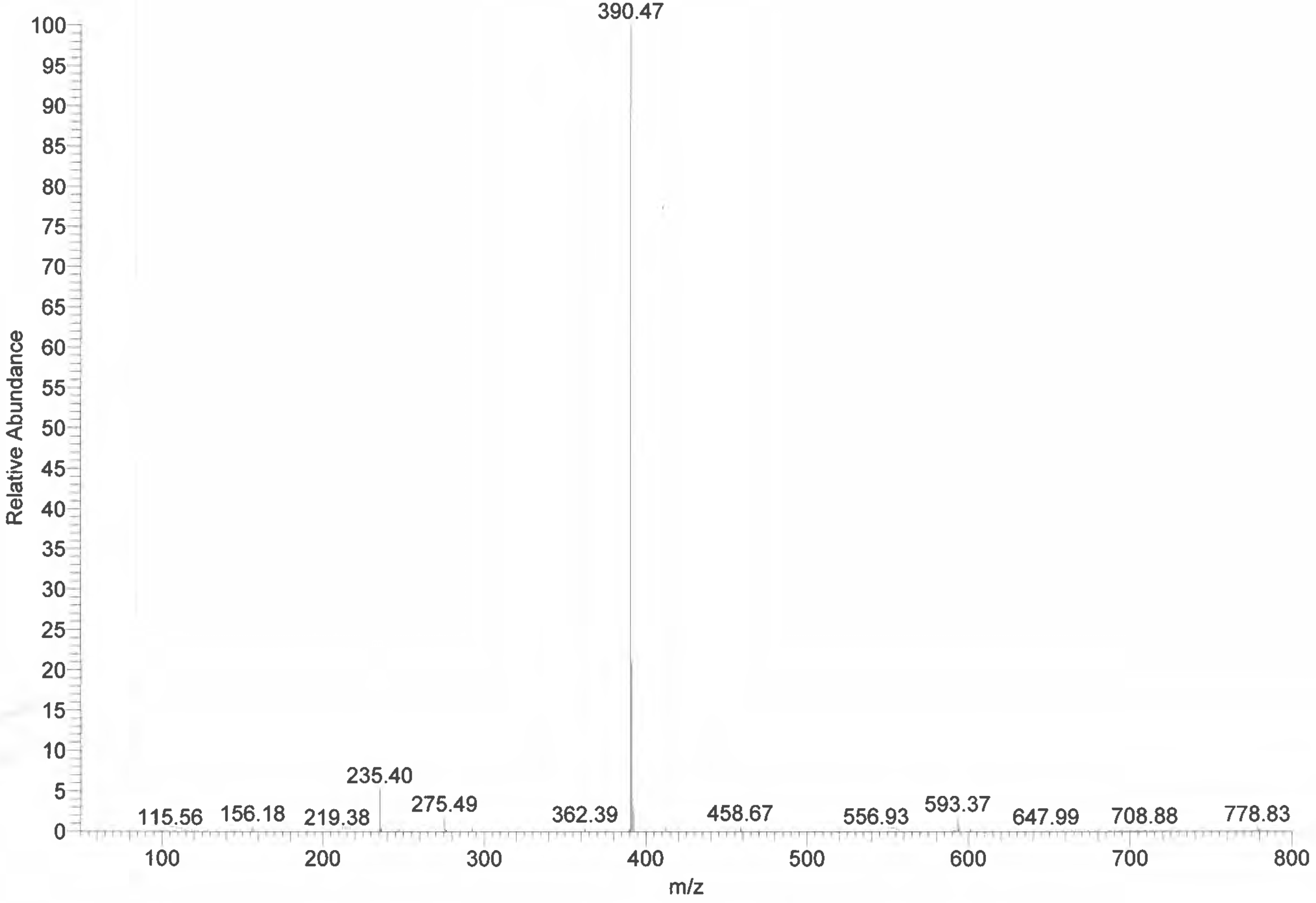

Cayman Chemical

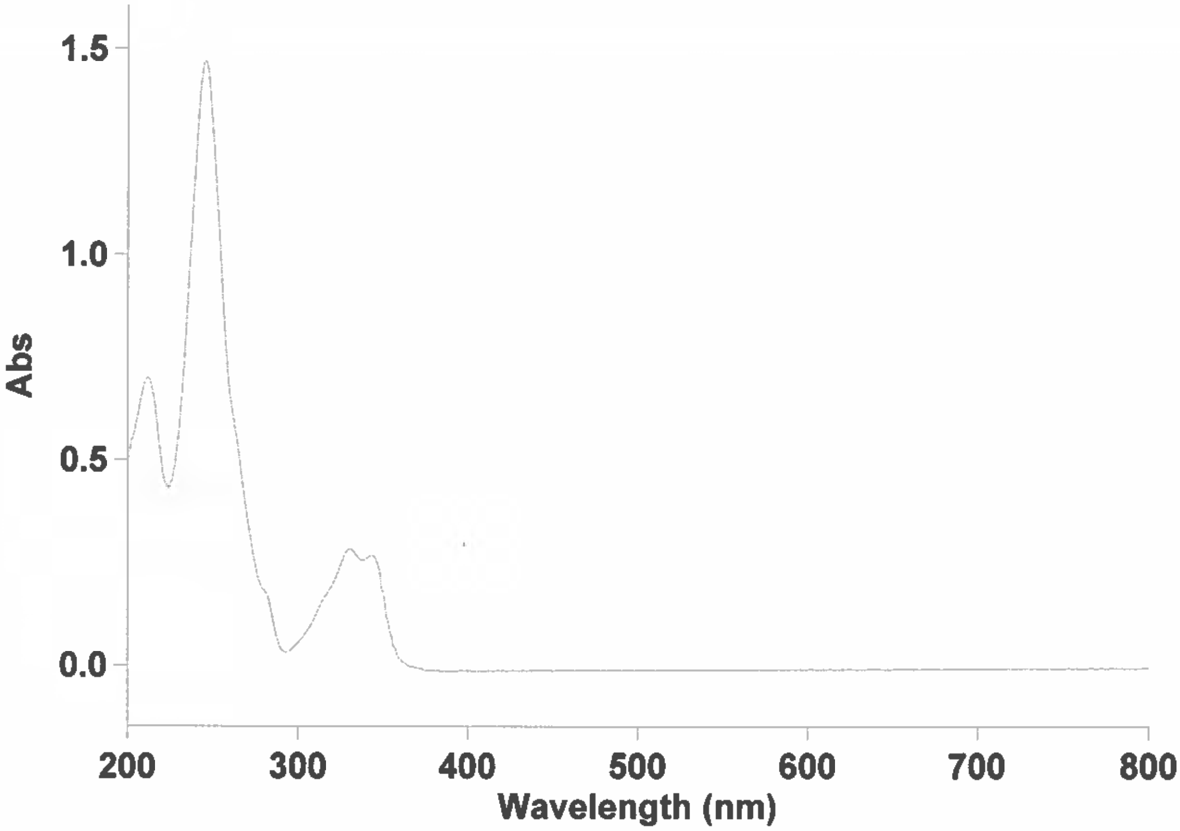

Scan Analysis Report

Report Time : Thu 20 May 03:41:21 PM 2010
Method:
Batch: C:\Varian\Cary Winuv\QC2010\qc01893.DSW
Software version: 3.00(303)
Operator:

Sample Name: sample3

Collection Time	5/20/2010 3:42:47 PM
Peak Table	
Peak Style	Peaks
Peak Threshold	0.0100
Range	800.00nm to 200.00nm

Wavelength (nm)	Abs
344.00	0.268
331.00	0.284
246.00	1.471
212.00	0.701

CERTIFICATE of ANALYSIS

Doxazosin (mesylate)

[4-(4-amino-6,7-dimethoxy-2-quinazolinyl)-1-piperazinyl](2,3-dihydro-1,4-benzodioxin-2-yl)-methanone, monomethanesulfonate

Item No. 18633 • Batch No. 0661669

Purity Specification: ≥98%
Molecular Formula.: C23H25N5O5 • CH3SO3H
CAS Number: 77883-43-3 Formula Weight: 547.6 Expiry date: 25OCT2026

Overview

Tests	Results
HPLC	Purity: 100.0 %
IR	Conforms
Mass spec	M-CH3SO3H: 452.5
TLC	Purity: 100 %
UV	λ max: 330, 247 nm
NMR	Conforms

Reviewed and approved by: Zach Mobley

WARNING
THIS PRODUCT IS FOR RESEARCH USE - NOT FOR HUMAN OR VETERINARY DIAGNOSTIC OR THERAPEUTIC USE. IT IS THE RESPONSIBILITY OF THE PURCHASER TO DETERMINE SUITABILITY FOR OTHER APPLICATIONS.

SAFETY DATA
This material should be considered hazardous until further information becomes available. Do not ingest, inhale, get in eyes, on skin, or on clothing. Wash thoroughly after handling. Before use, the user must review the complete Safety Data Sheet, which has been sent via email to your institution.

WARRANTY AND LIMITATION OF REMEDY
Buyer agrees to purchase the material subject to Cayman's Terms and Conditions. Complete Terms and Conditions including Warranty and Limitation of Liability information can be found on our website.

Copyright Cayman Chemical Company, 10/12/2018

CAYMAN CHEMICAL
1180 EAST ELLSWORTH RD
ANN ARBOR, MI 48108 · USA
PHONE: [800] 364-9897
[734] 971-3335
FAX: [734] 971-3640
CUSTSERV@CAYMANCHEM.COM
WWW.CAYMANCHEM.COM

===

Acq. Operator : Iris Ho   Seq. Line : 1
Acq. Instrument : HPLC6   Location : Vial 1
Injection Date : 11/21/2022 10:25:40 AM   Inj : 1
Inj Volume : 25.0 µl
Acq. Method : C:\CHEM32\1\DATA\CURRENT 2022-11-21 10-24-19\18633.M
Last changed : 11/21/2022 10:36:47 AM by Iris Ho
(modified after loading)
Analysis Method : C:\CHEM32\1\METHODS\90_10 RINSE.M
Last changed : 11/10/2022 11:42:18 AM by Iris Ho
Sample Info : Doxazosin (mesylate); Cat #18633; QC67554; QC
Gemini C18; 5µ; 4.6x250mL
247 nm; p=143 bar; 1mL/min
70:30; MeOH:NH4OAc (10mM pH=7.2)

Additional Info : Peak(s) manually integrated

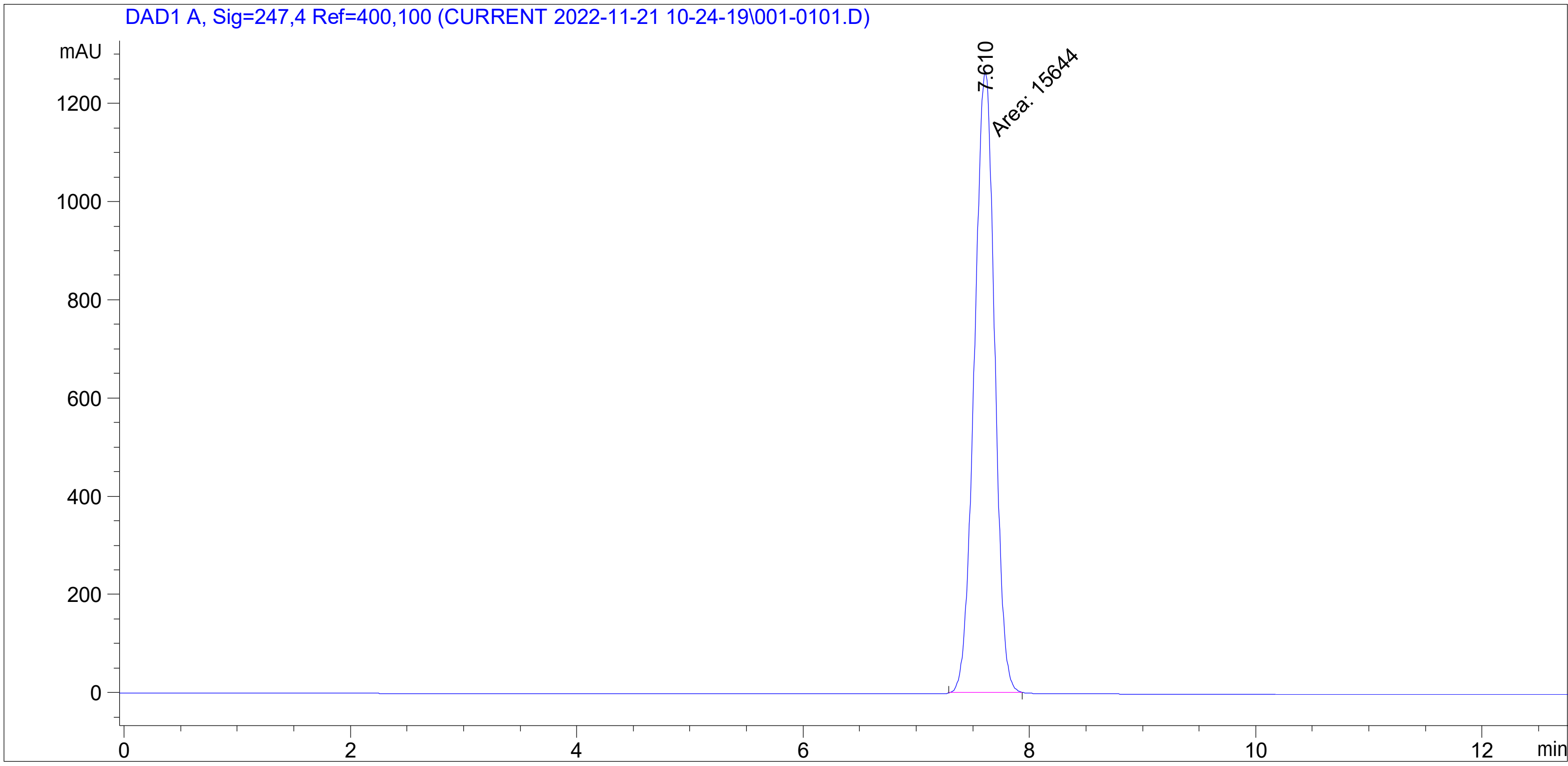

===
Area Percent Report
===

Sorted By : Signal
Multiplier: : 1.0000
Dilution: : 1.0000
Use Multiplier & Dilution Factor with ISTDs

Signal 1: DAD1 A, Sig=247,4 Ref=400,100

Peak #	RetTime [min]	Type	Width [min]	Area [mAU*s]	Height [mAU]	Area %
1	7.610	MM	0.2061	1.56440e4	1265.12561	100.0000

Totals : 1.56440e4 1265.12561

*** End of Report ***

PerkinElmer Spectrum IR Version 10.6.1
Monday, November 21, 2022 7:22 AM

Analyst	Administrator
Date	Monday, November 21, 2022 7:22 AM

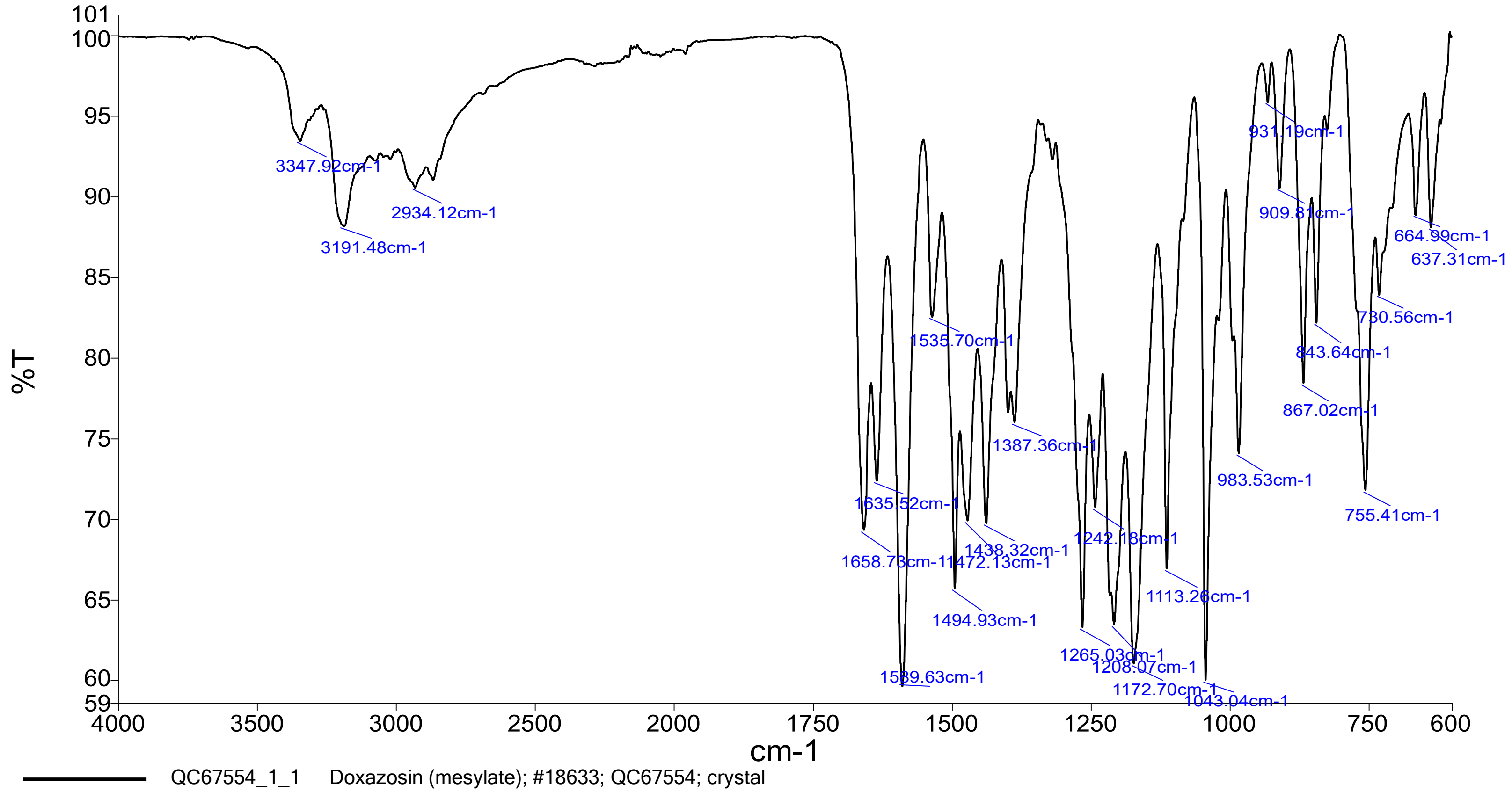

C:\Thermo\...\LTQ\data\18633_QC67554 11/21/22 13:38:55 Doxazosin (mesylate)
AMNESILOWFLOW

18633_QC67554 #41 RT: 0.30 AV: 1 NL: 7.67E4
T: ITMS + c ESI Full ms [105.00-800.00]

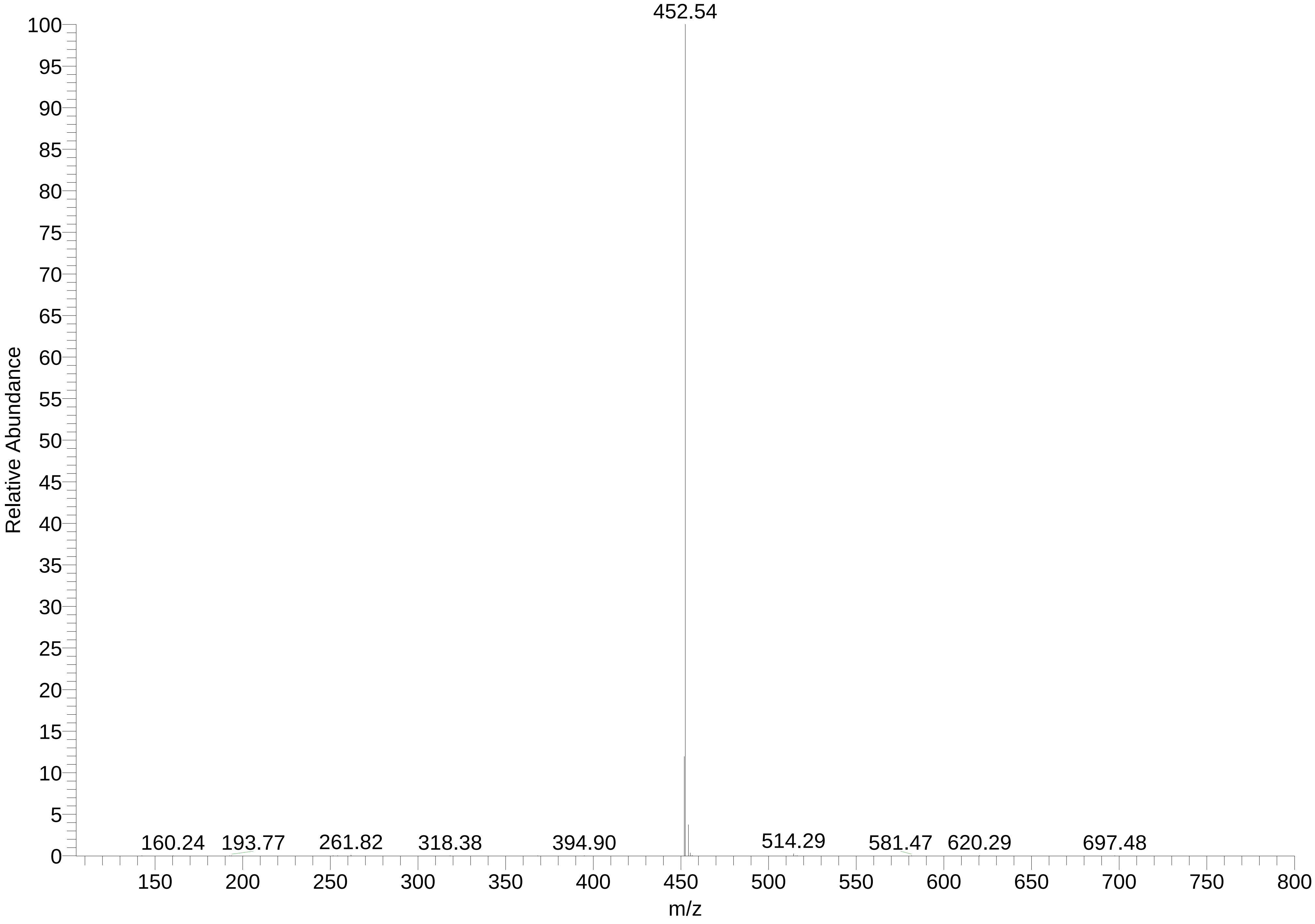

Doxazosin (mesylate)

Item #18633

Batch #0661669

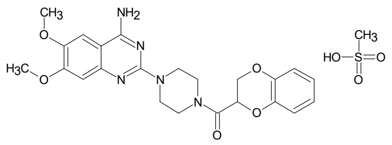

File Name	\\sulfur\private\nmrdata\JEOL_2022\18633-0661669\18633-0661669_PROTON_01-Dec-2022-1-1.jdf				
Date	01 Dec 2022 14:29:01	Nucleus	1H	Frequency (MHz)	399.5822
Solvent	DMSO-d6	Number of Transients	16	Origin	JEOL ECZ400S Sc v601
Temperature (degree C)	20.500				

^{1}H NMR (DMSO-d_6, 400 MHz) δ 11.65 (s, 1H), 8.80 (br s, 1H), 8.71 (br s, 1H), 7.65 (s, 1H), 7.14 (s, 1H), 6.8-6.9 (m, 4H), 5.29 (dd, 1H, *J*=2.5, 6.6 Hz), 4.40 (dd, 1H, *J*=2.5, 11.9 Hz), 4.18 (dd, 1H, *J*=6.5, 11.8 Hz), 3.8-3.9 (m, 6H), 3.81 (s, 6H), 3.64 (br s, 2H), 2.25 (s, 3H)

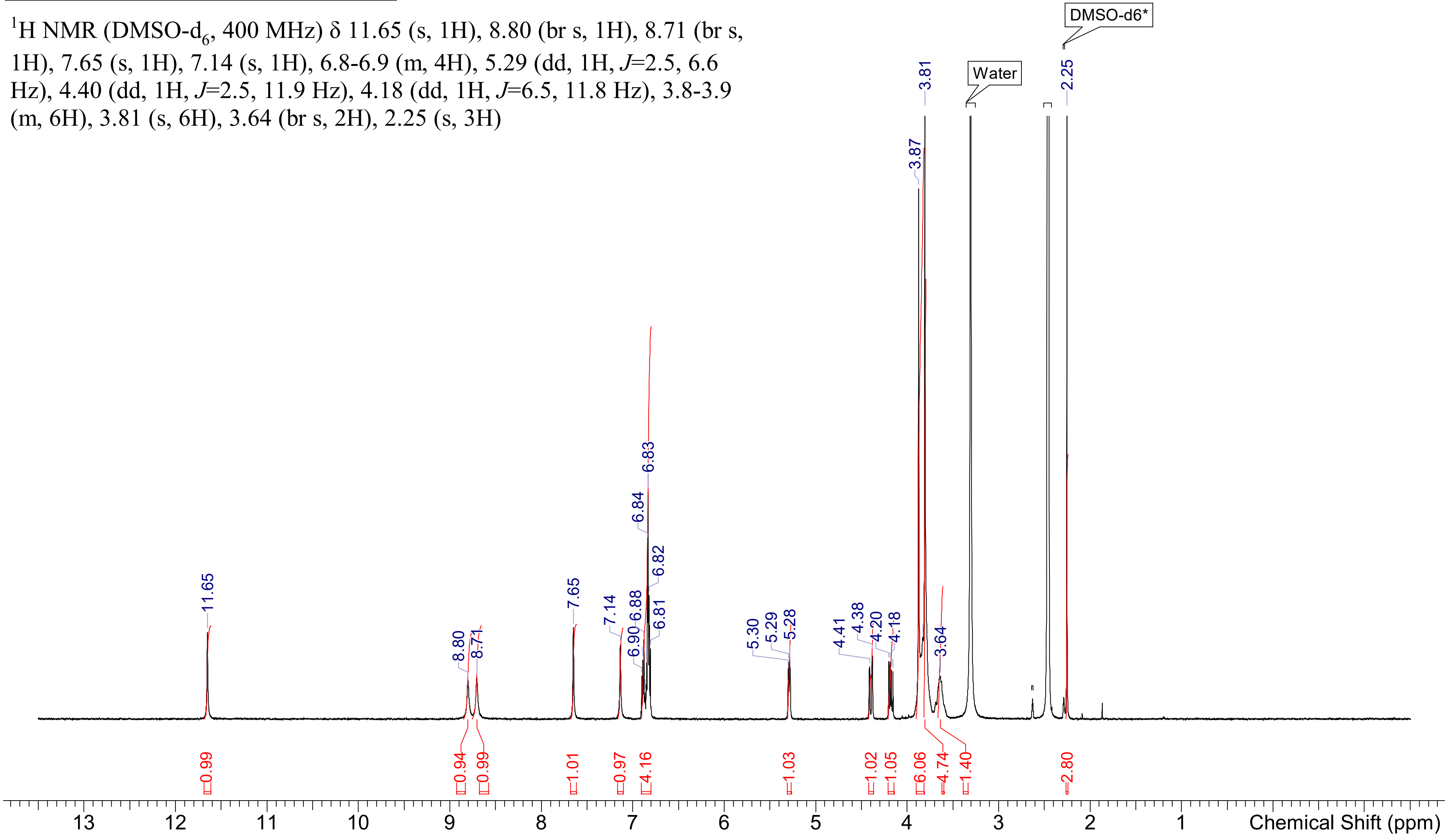

11/21/2022 7:26:45 AM

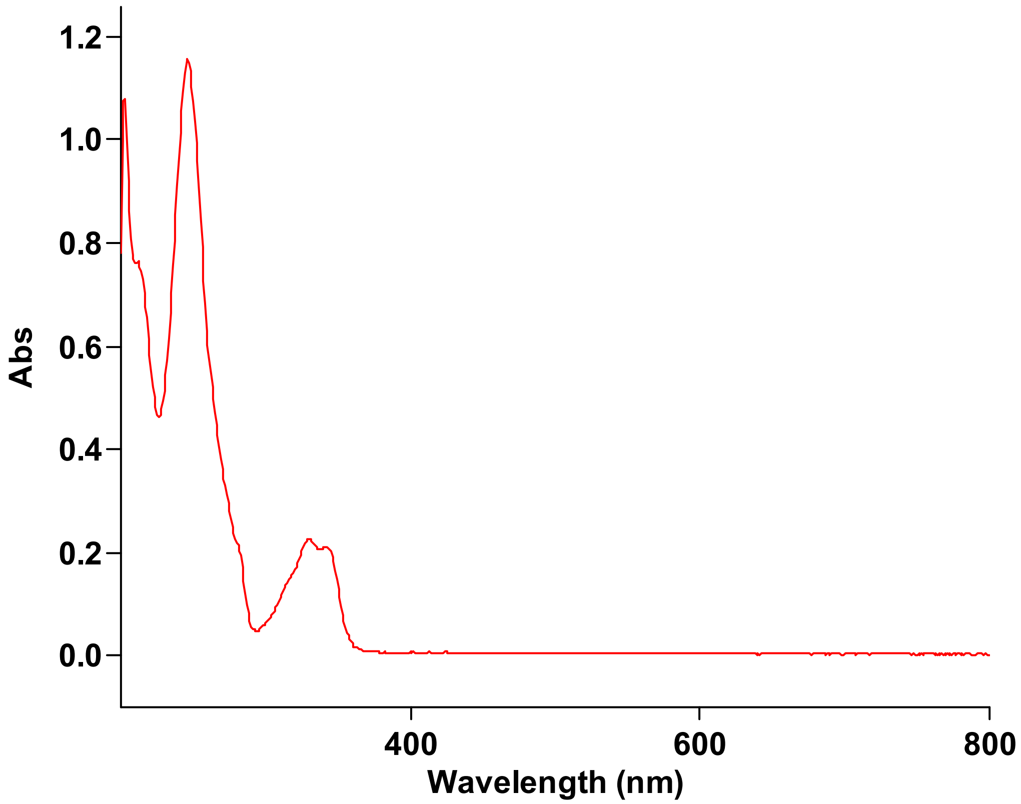

Cayman Chemical Company

Doxazosin (mesylate)
#18633 QC67554
A Solution in Methanol

**Sample Name:**	**sample79**
Collection Time	11/21/2022 7:26:13 AM

Peak Table	
Peak Style	Peaks
Peak Threshold	0.0100
Range	800.0nm to 200.0nm
Wavelength (nm)	Abs
330.0	0.226
246.0	1.156
203.0	1.078

CERTIFICATE of ANALYSIS

Cayman CHEMICAL

Terazosin (hydrochloride)

[4-(4-amino-6,7-dimethoxy-2-quinazolinyl)-1-piperazinyl](tetrahydro-2-furanyl)-methanone, monohydrochloride

Item No. 20216 • Batch No. 0502470

Purity Specification: ≥98%
Molecular Formula.: C19H25N5O4 • HCl
CAS Number: 63074-08-8 Formula Weight: 423.9 Expiry date: 01JUN2027

Overview

Tests	Results
HPLC	Purity: 100.0 %
IR	Conforms
Mass spec	M-HCl: 388.3
Melting Point	300 - 304 ºC (dec.)
TLC	Purity: 100 %
UV	λ max: 214, 247, 0 nm

Reviewed and approved by: Jennifer LaBrecque

Jen LaBrecque

WARNING
THIS PRODUCT IS FOR RESEARCH USE - NOT FOR HUMAN OR VETERINARY DIAGNOSTIC OR THERAPEUTIC USE. IT IS THE RESPONSIBILITY OF THE PURCHASER TO DETERMINE SUITABILITY FOR OTHER APPLICATIONS.

SAFETY DATA
This material should be considered hazardous until further information becomes available. Do not ingest, inhale, get in eyes, on skin, or on clothing. Wash thoroughly after handling. Before use, the user must review the complete Safety Data Sheet, which has been sent via email to your institution.

WARRANTY AND LIMITATION OF REMEDY
Buyer agrees to purchase the material subject to Cayman's Terms and Conditions. Complete Terms and Conditions including Warranty and Limitation of Liability information can be found on our website.

Copyright Cayman Chemical Company, 10/12/2018

CAYMAN CHEMICAL
1180 EAST ELLSWORTH RD
ANN ARBOR, MI 48108 · USA
PHONE: [800] 364-9897
[734] 971-3335
FAX: [734] 971-3640
CUSTSERV@CAYMANCHEM.COM
WWW.CAYMANCHEM.COM

Data File C:\CHEM32\1\DATA\20K\QC222322.D
Sample Name: Terazosin (hydrochloride)

===

Acq. Operator : SYSTEM
Acq. Instrument : Instrument 3 Location : Vial 0
Injection Date : 5/31/2017 11:07:20 AM
Acq. Method : C:\CHEM32\1\METHODS\GENERIC.M
Last changed : 5/31/2017 10:46:14 AM by SYSTEM
(modified after loading)
Analysis Method : C:\CHEM32\1\METHODS\GENERIC.M
Last changed : 5/30/2017 3:22:46 PM by SYSTEM
Sample Info : Terazosin (hydrochloride); Cat#20216; QC22232; New QC
Gemini C18, 5µ, 4.6 x 250mm
247 nm; p=102 bar; 1 mL/min
50:50; MeOH:NH4OAc (10mM, pH 7.2)

Additional Info : Peak(s) manually integrated

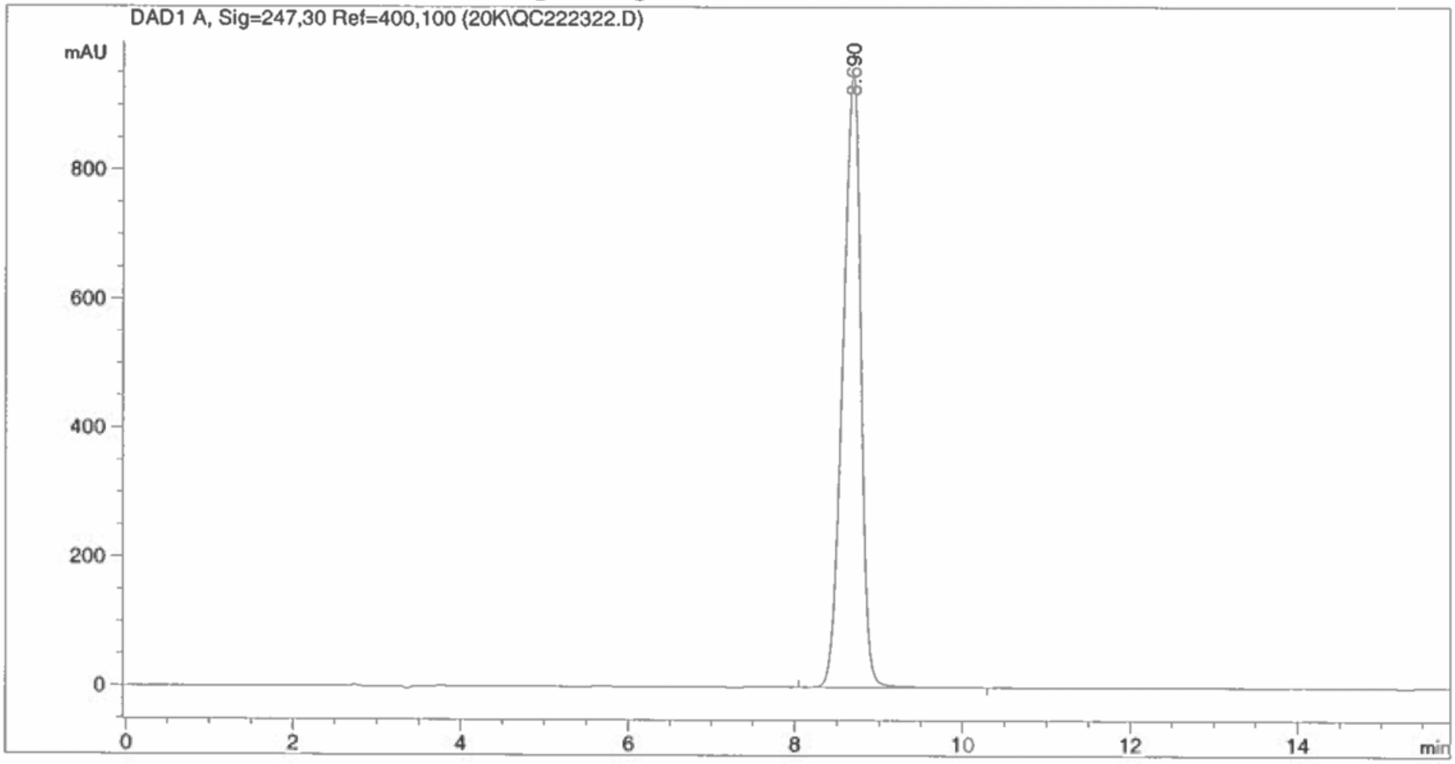

===

Area Percent Report

===

Sorted By : Signal
Multiplier : 1.0000
Dilution : 1.0000
Sample Amount: : 10.00000 [ng/ul] (not used in calc.)
Do not use Multiplier & Dilution Factor with ISTDs

Signal 1: DAD1 A, Sig=247,30 Ref=400,100

Peak #	RetTime [min]	Type	Width [min]	Area [mAU*s]	Height [mAU]	Area %
1	8.690	BB	0.2323	1.42006e4	950.49957	100.0000

Totals : 1.42006e4 950.49957

PerkinElmer Spectrum Version 10.03.02
Wednesday, May 24, 2017 10:05 AM

Analyst Administrator
Date Wednesday, May 24, 2017 10:05 AM

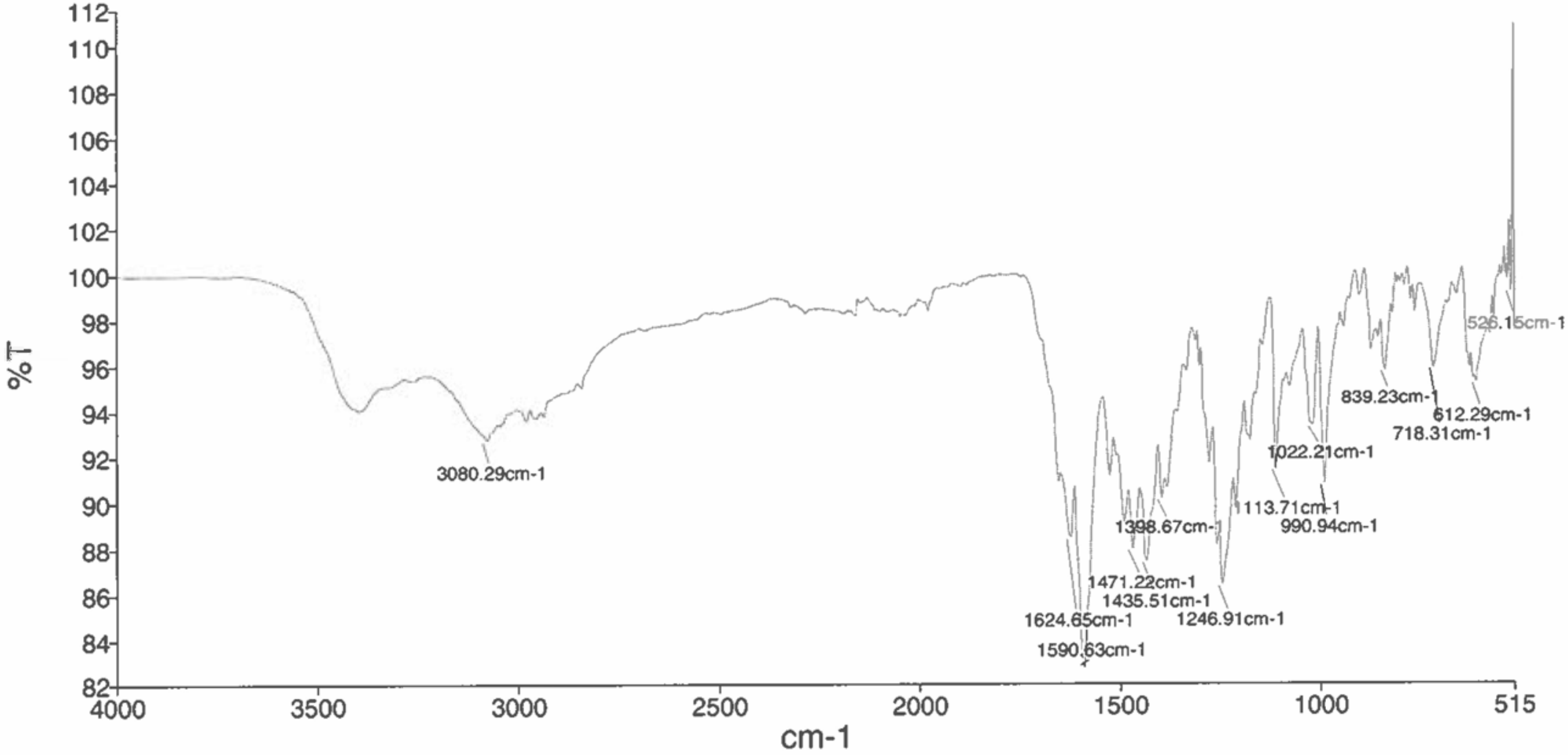

QC22232_1_1 Terazosin (hydrochloride); #20216; QC22232; crystal

C:\MassSpecData\2017\20216_QC22232 | 5/23/2017 11:36:26 AM | Terazosin (hydrochloride)

Nutralapciposhiflo

20216_QC22232 #22-27 RT: 0.15-0.18 AV: 6 NL: 2.87E4

T: ITMS + c APCI corona Full ms [105.00-700.00]

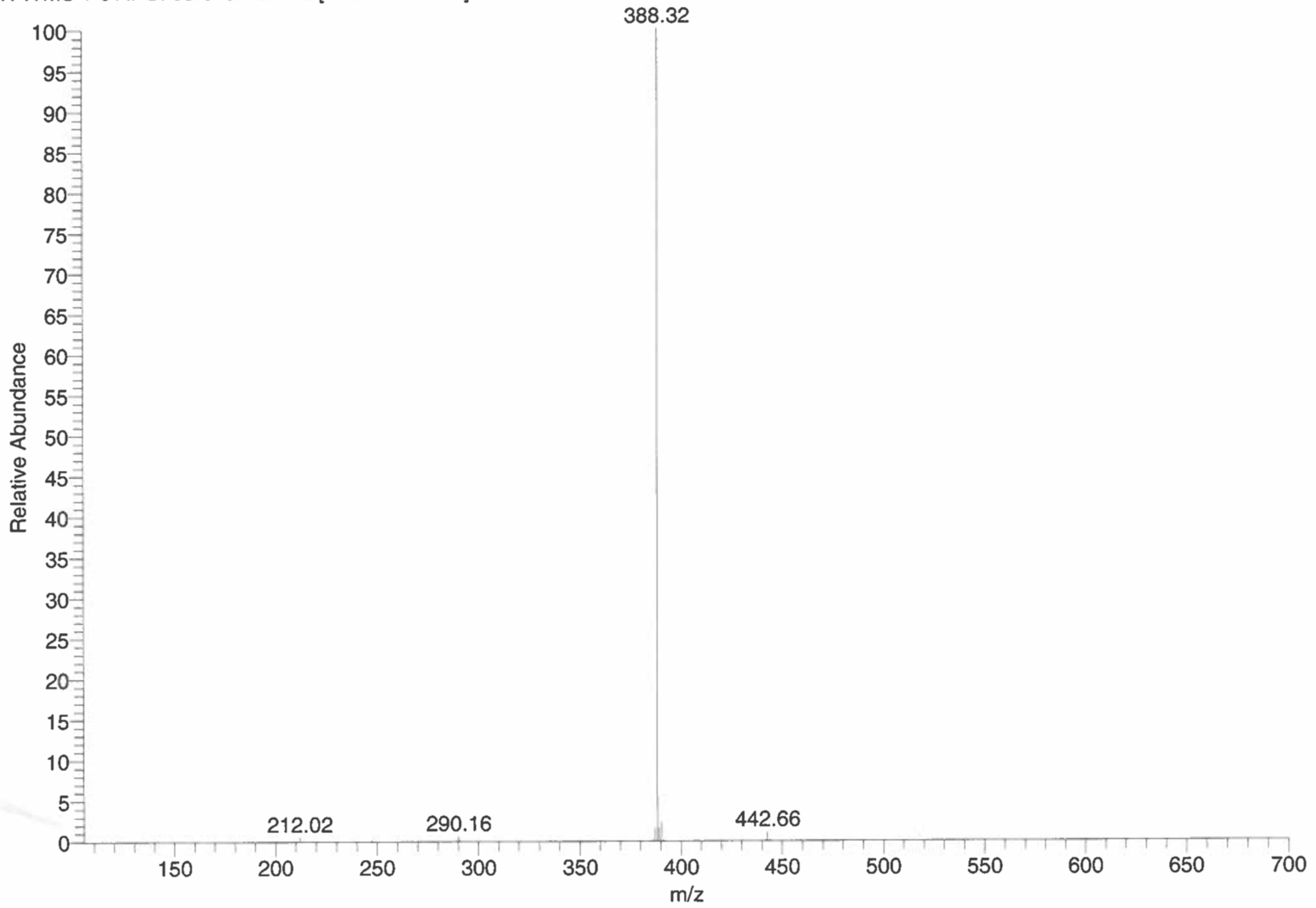

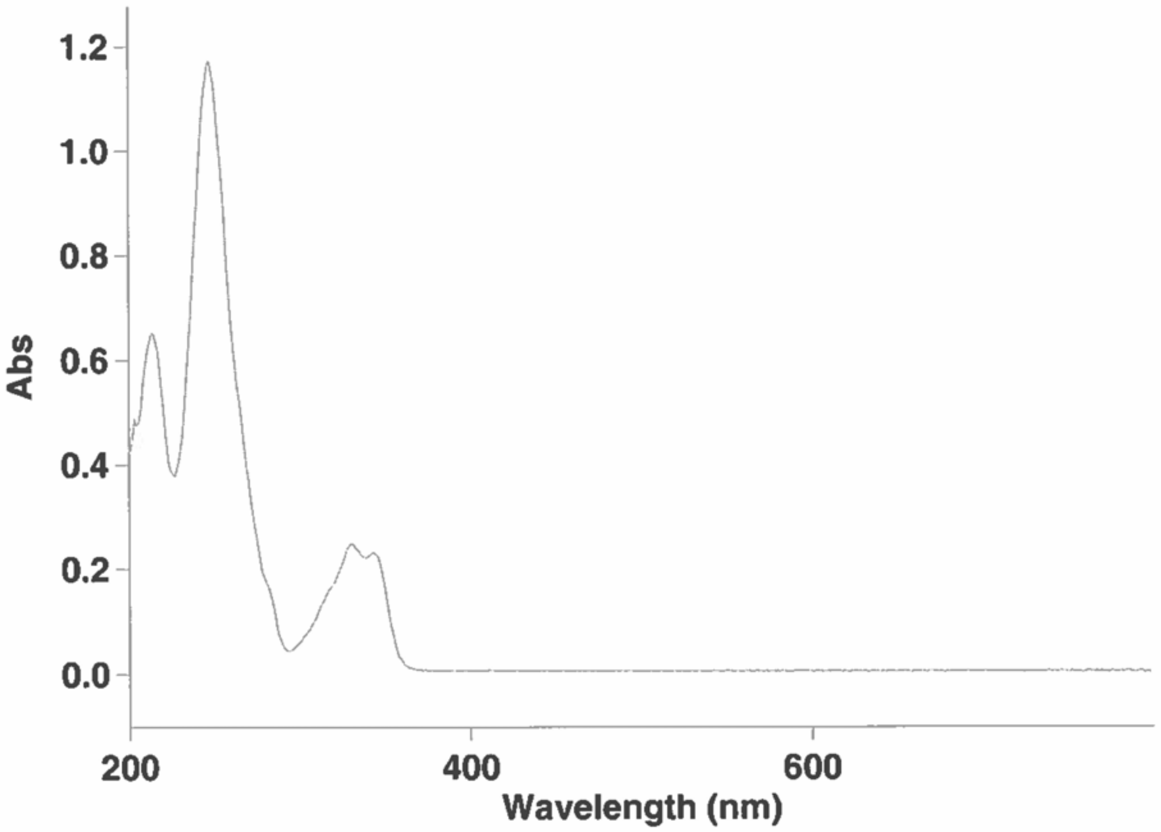

Terazosin (hydrochloride)
Cat #20216 QC22232
A solution in Ethanol

Sample Name: sample11

Collection Time	5/26/2017 8:58:07 AM
Peak Table	
Peak Style	Peaks
Peak Threshold	0.0100
Range	800.0nm to 200.0nm

Wavelength (nm)	Abs
331.0	0.248
247.0	1.172
214.0	0.652
203.0	0.489

MaxPeak: 100.00%
Ret_Time: 1.168 min

BG443987$1

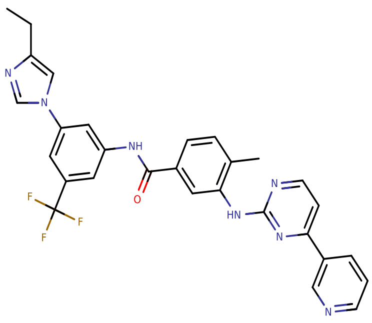

Mol Wt 543.54

Exact Mass 543.23

#	Time	Area%
1	1.168	100.00

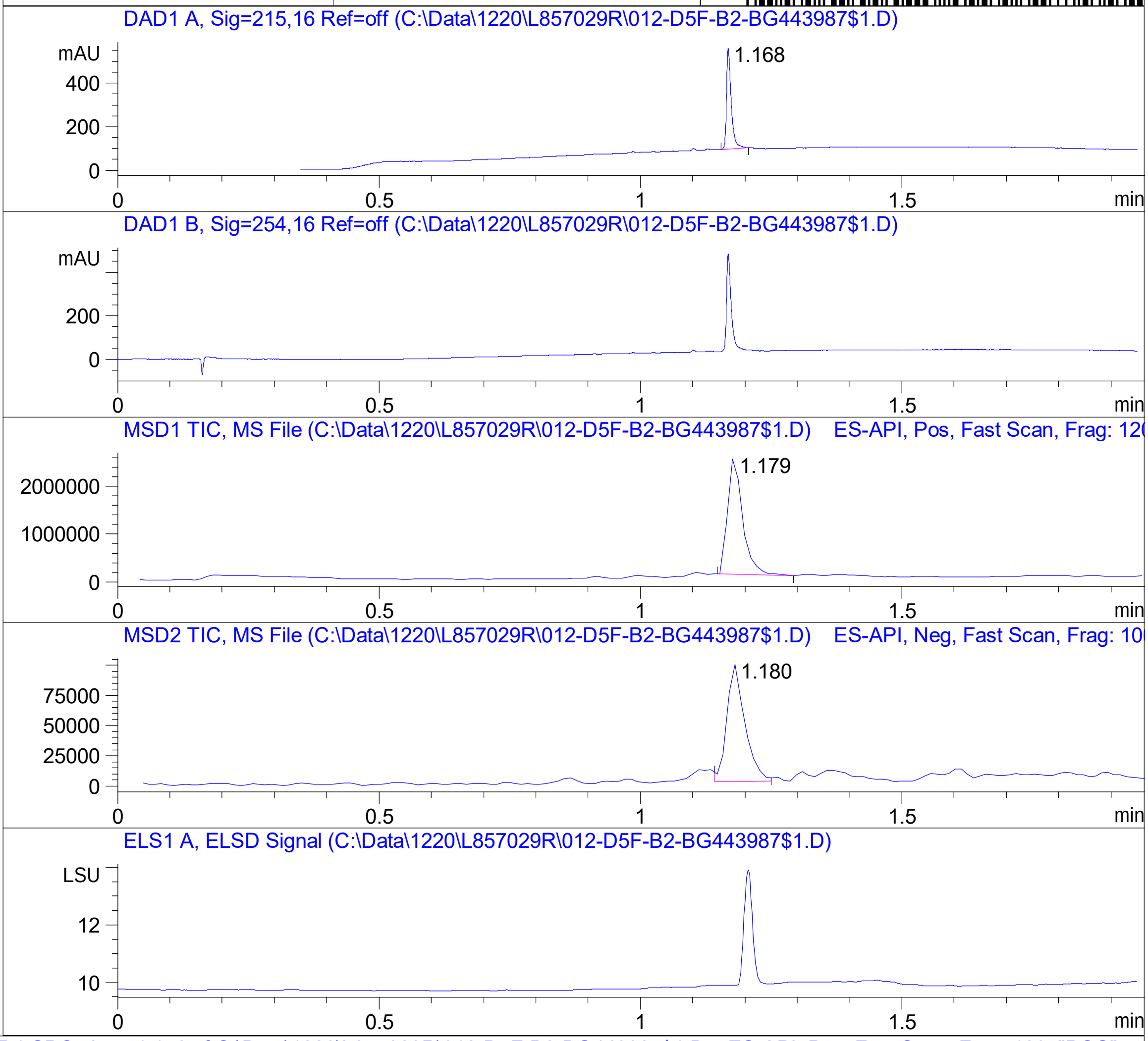

RT 1.179

RT 1.180

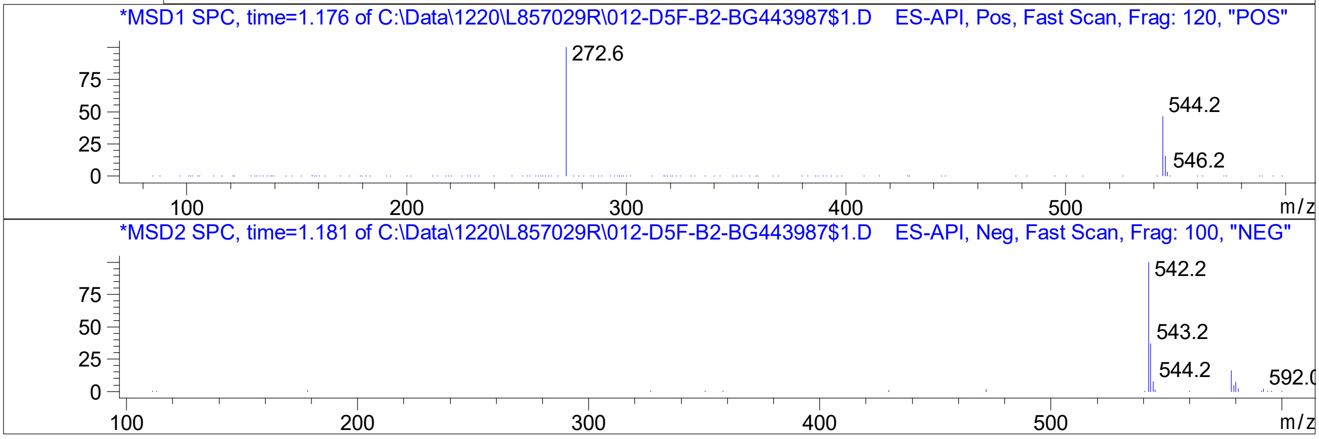

Inj.Date 12/20/2024 NEW
C:\Users\Public\Documents\ChemStation\1\Data\12_20\L857029R\SUPOR_30.M

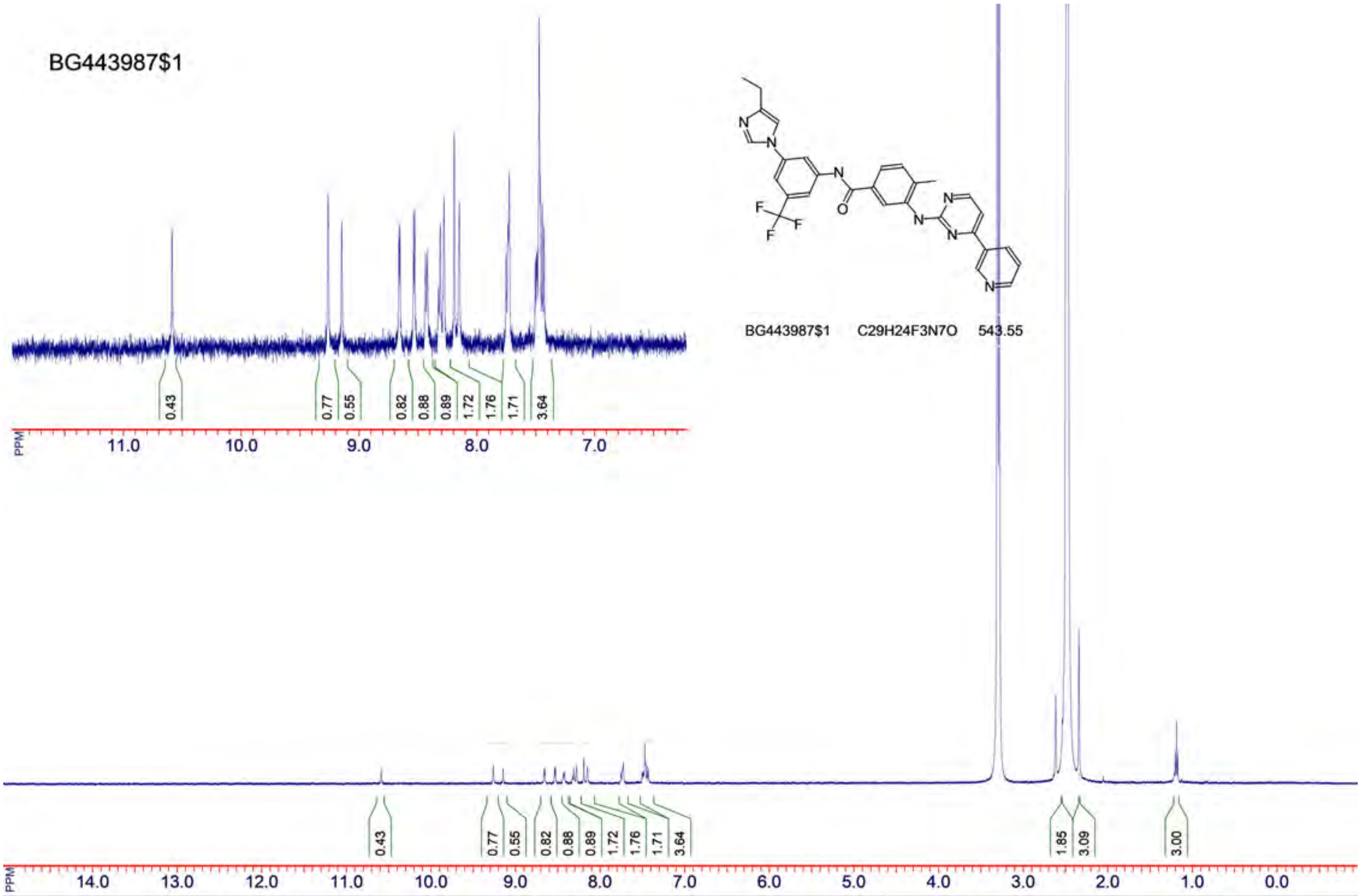

**File name: BG443987$1**	Operator: Victorova	SF: 499.8211 MHz	NSC: 0	PW: 3.27 usec, RG: 40	SI: 131072
Date: 20-Dec-2024	Solvent: dmso	SW: 10965 Hz	TE: 293 K	AQ: 2.92 sec, RD: 0.00 sec	

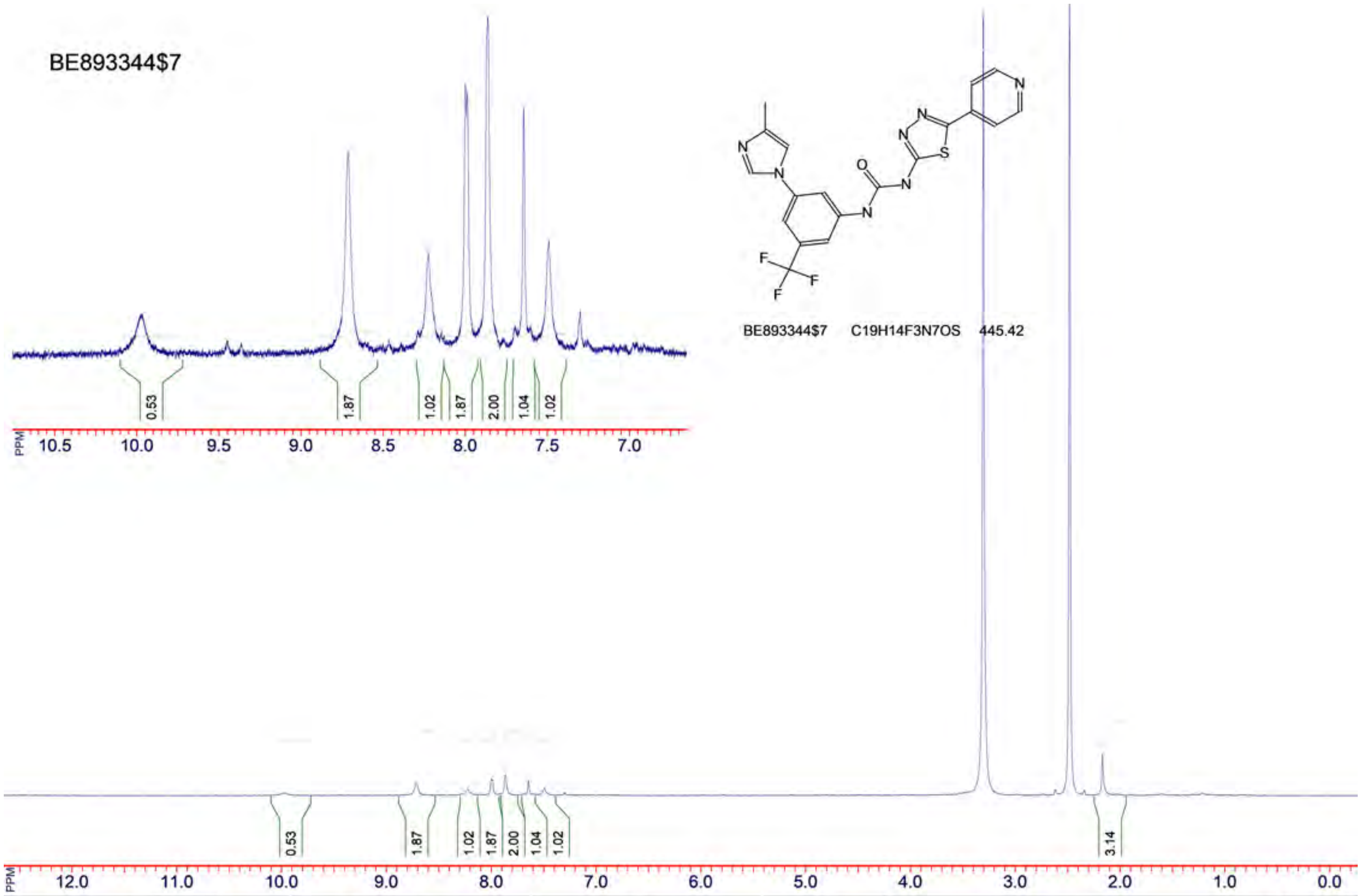

**File name: BE893344$7**	Operator: Victorova	SF: 499.8201 MHz	NSC: 0	PW: 9.80 usec, RG: 24	SI: 131072
Date: 15-Jul-2024	Solvent: dmso	SW: 8993 Hz	TE: 293 K	AQ: 2.67 sec, RD: 0.00 sec	

MaxPeak: 97.26%
Ret_Time: 1.129 min

BG210459$2

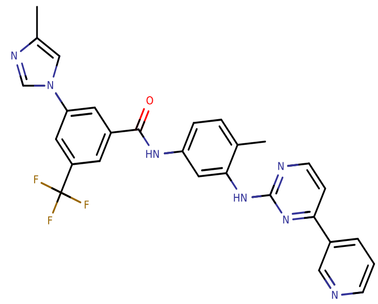

Mol Wt 529.52

Exact Mass 529.21

#	Time	Area%
1	1.114	2.74
2	1.129	97.26

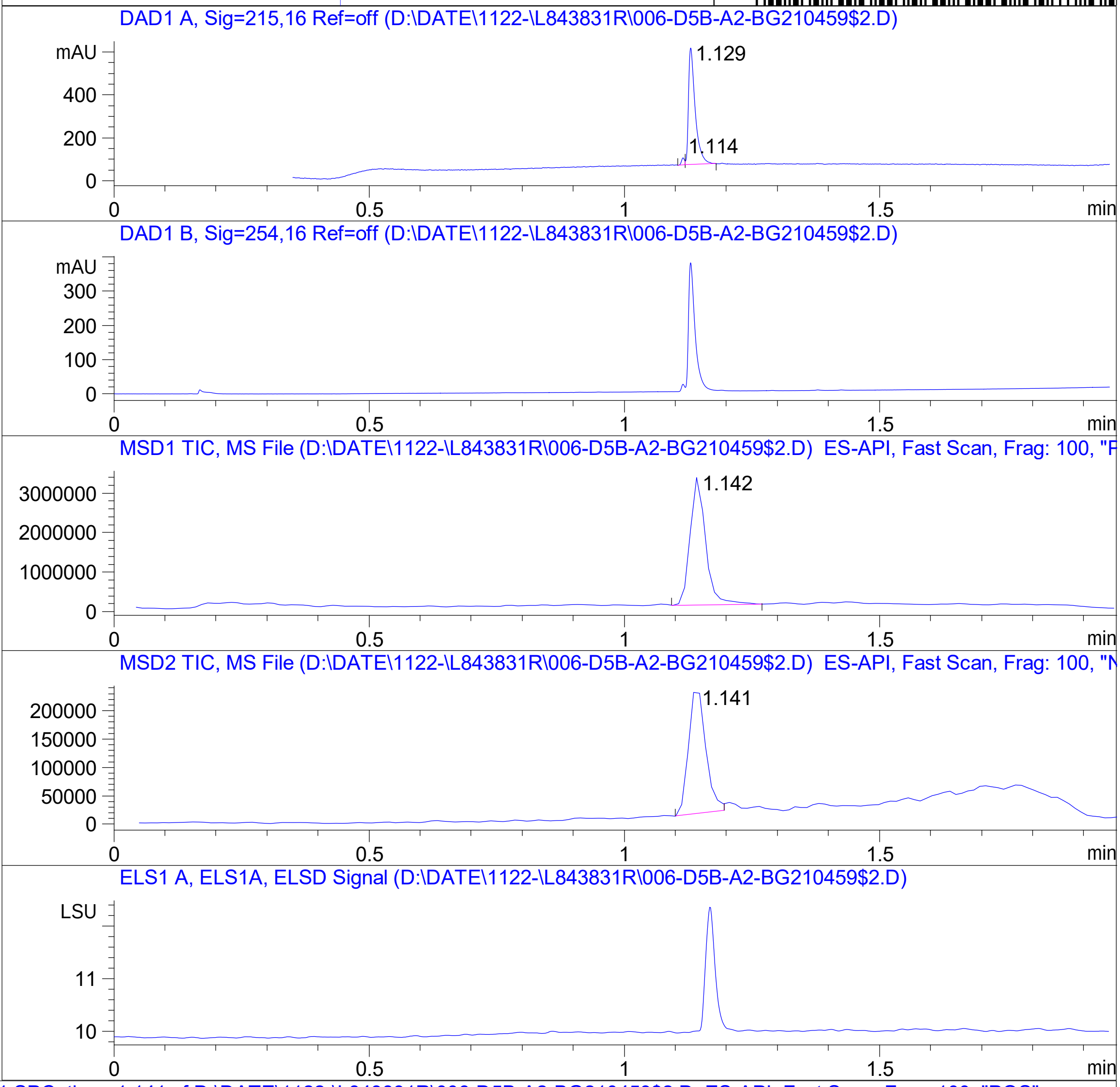

RT 1.142

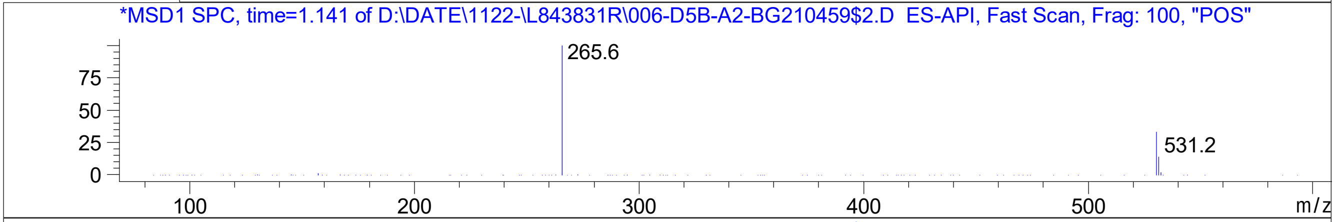

RT 1.141

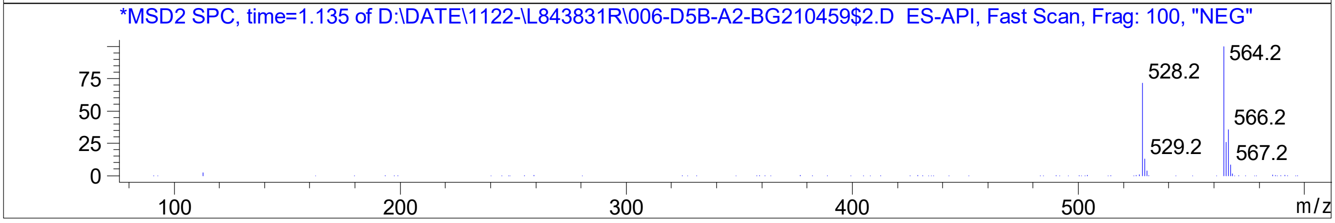

Inj.Date 11/22/2024 E

C:\Users\Public\Documents\ChemStation\1\Data\11_22\L843831R\SUPOR_30.M

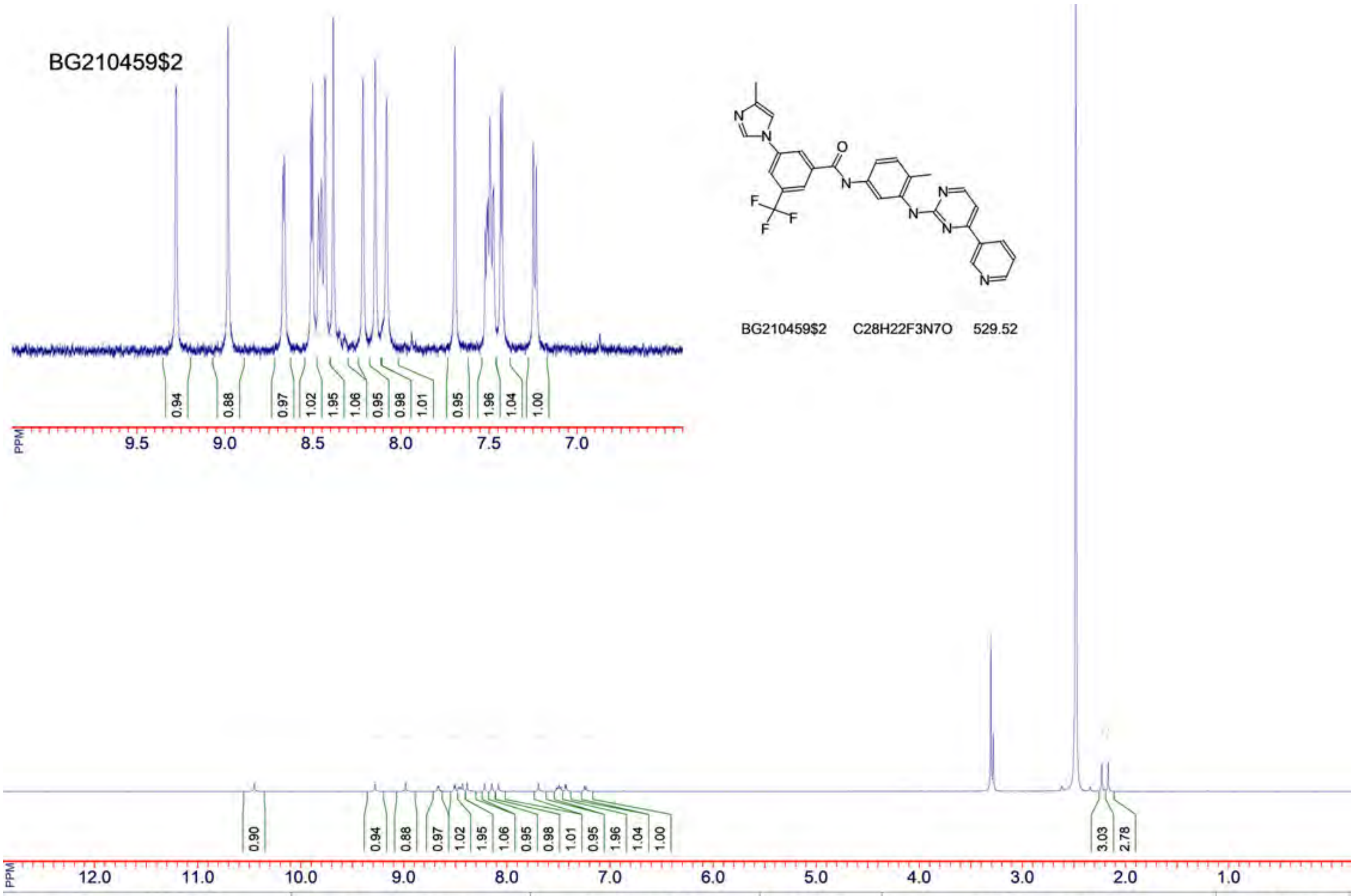

**File name: BG210459$2**	Operator: Victorova	SF: 499.8201 MHz	NSC: 0	PW: 9.00 usec, RG: 20	SI: 131072
Date: 22-Nov-2024	Solvent: dmso	SW: 8993 Hz	TE: 298 K	AQ: 2.67 sec, RD: 0.00 sec	

MaxPeak: 98.36%
Ret_Time: 0.930 min

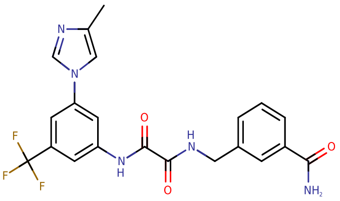

Mol Wt 445.4

Exact Mass 445.15

#	Time	Area%
1	0.840	1.64
2	0.930	98.36

BE893345$2

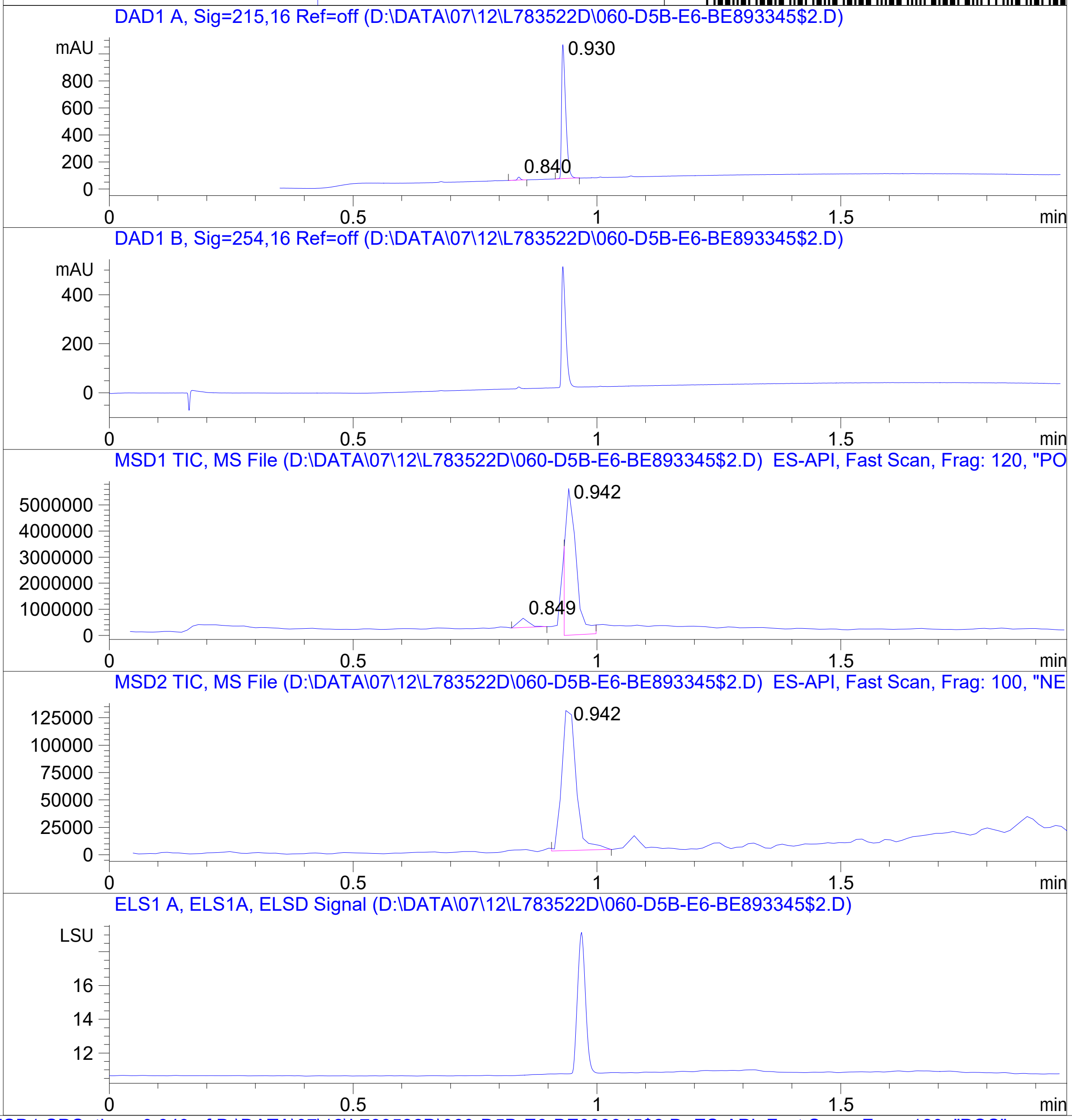

RT 0.849

RT 0.942

RT 0.942

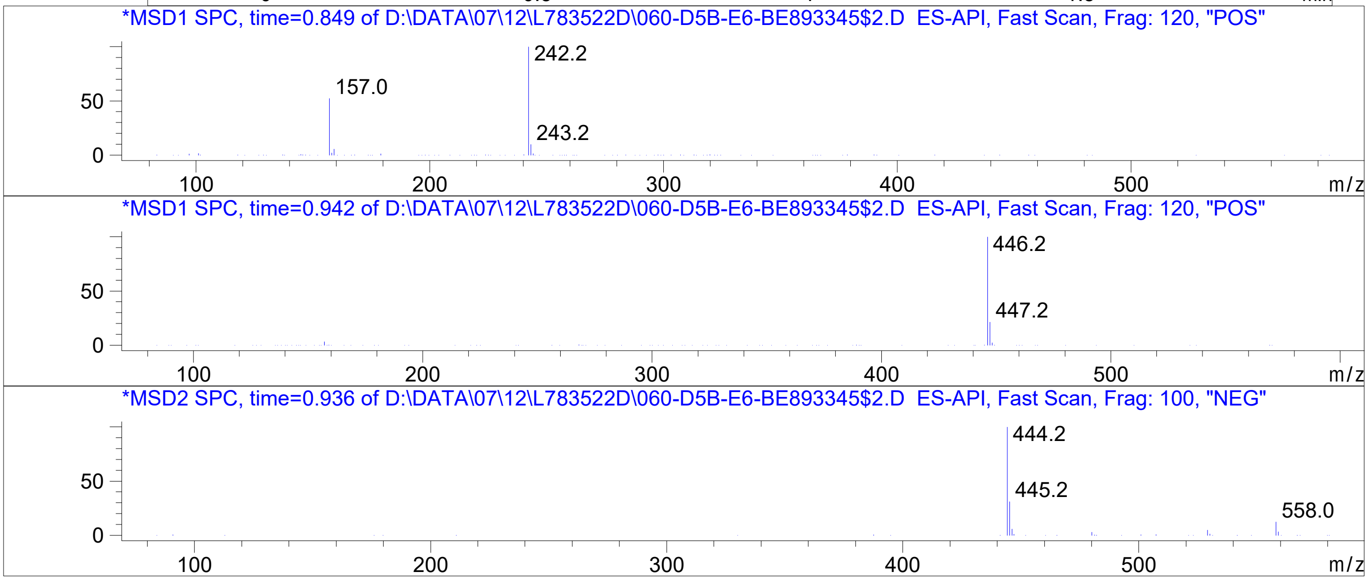

BE893334$1

MaxPeak: 93.36%
Ret_Time: 1.058 min

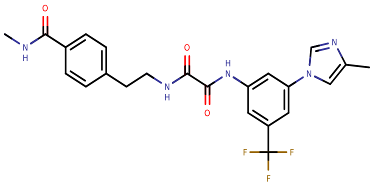

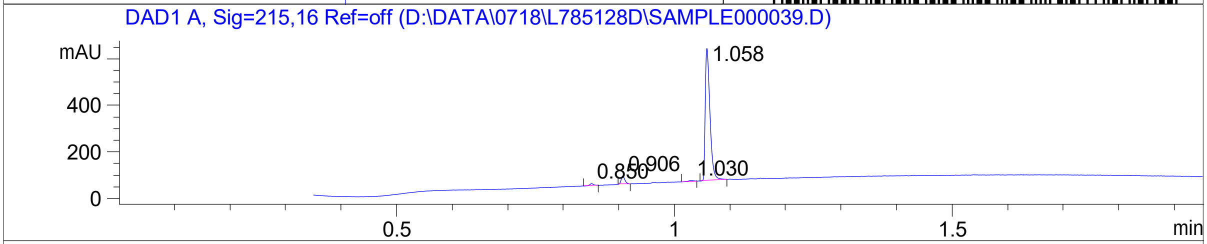

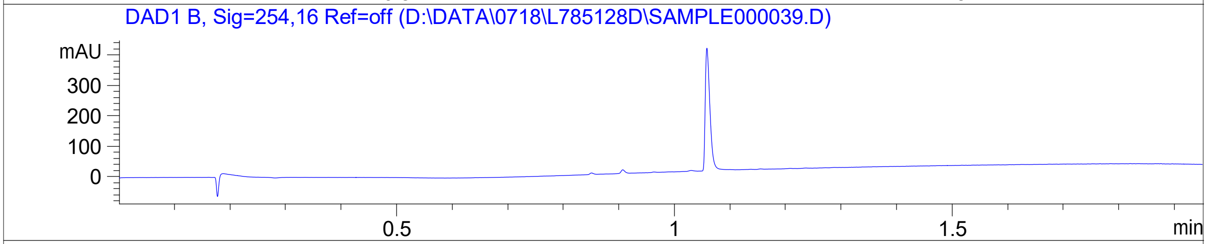

Mol Wt 473.45

Exact Mass 473.19

#	Time	Area%
1	0.850	1.15
2	0.906	4.51
3	1.030	0.99
4	1.058	93.36

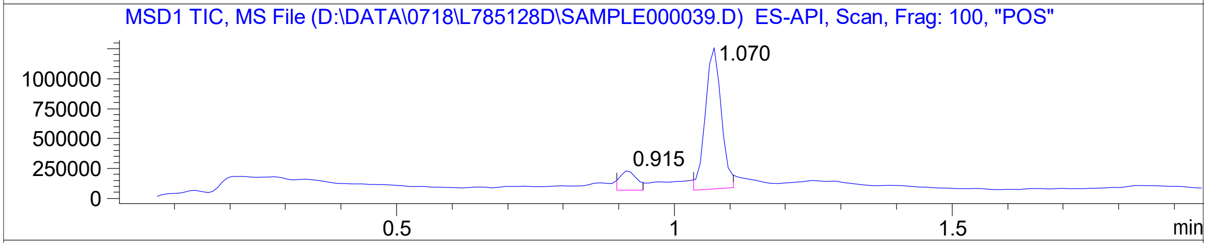

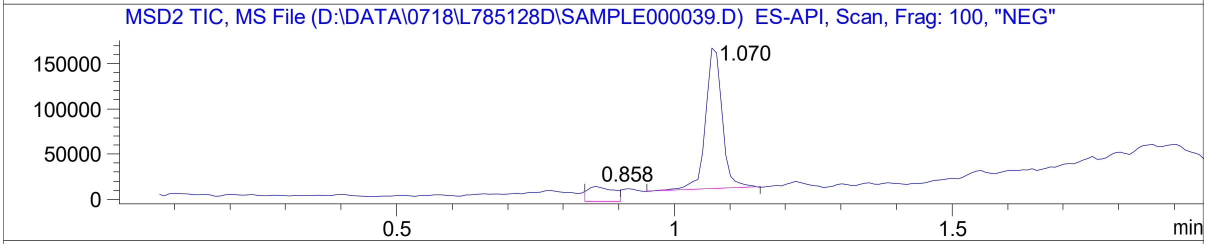

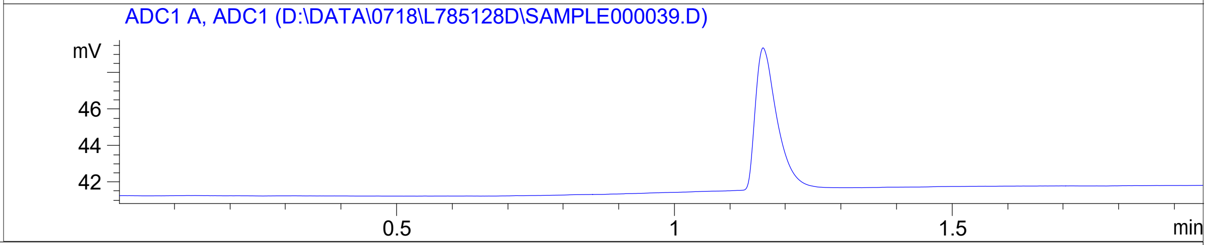

RT 0.915

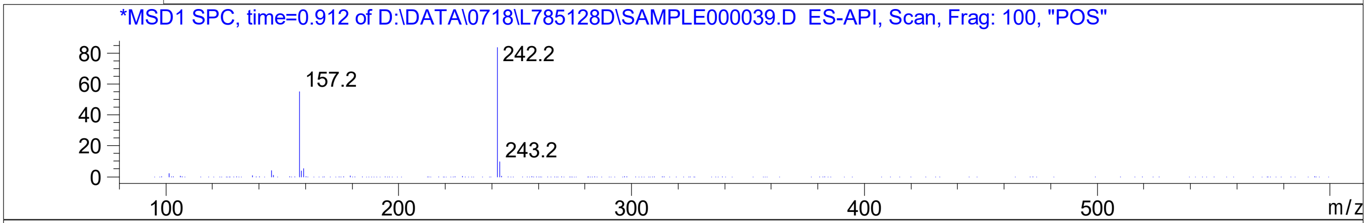

RT 1.070

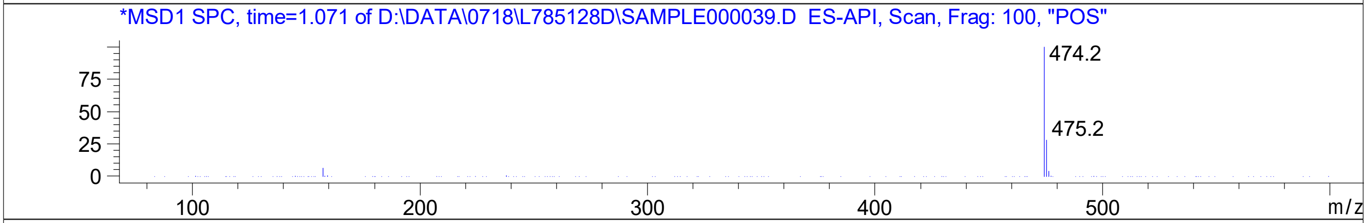

RT 0.858

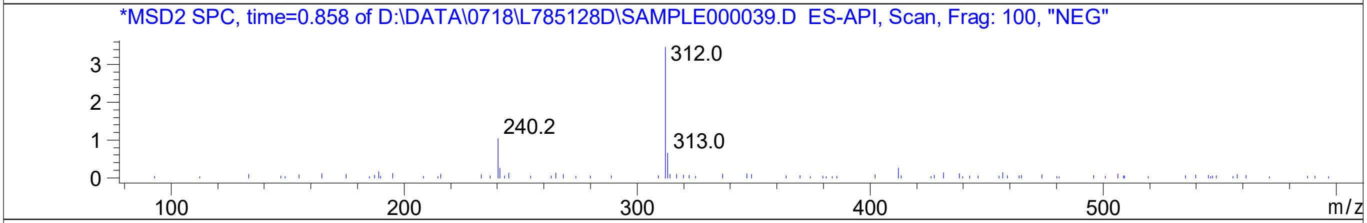

RT 1.070

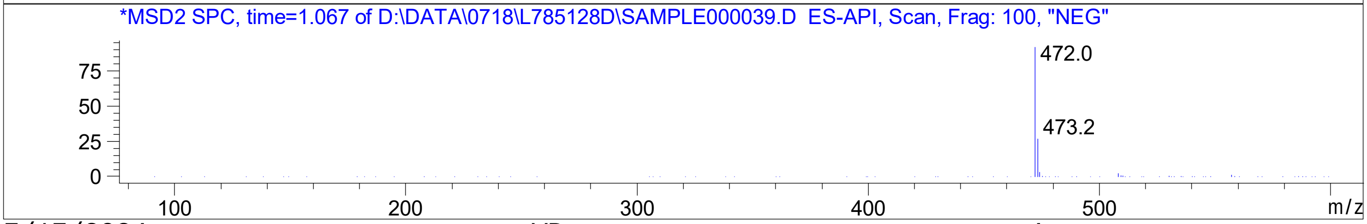

Inj.Date 7/17/2024 VB

C:\CHEM32\1\METHODS\SUPOR_30.M

MaxPeak: 100.00%
Ret_Time: 0.959 min

BF104206$2

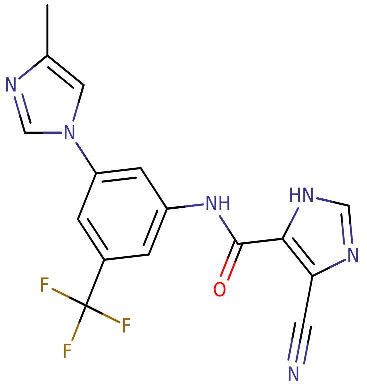

Mol Wt 360.29

Exact Mass 360.1

#	Time	Area%
1	0.959	100.00

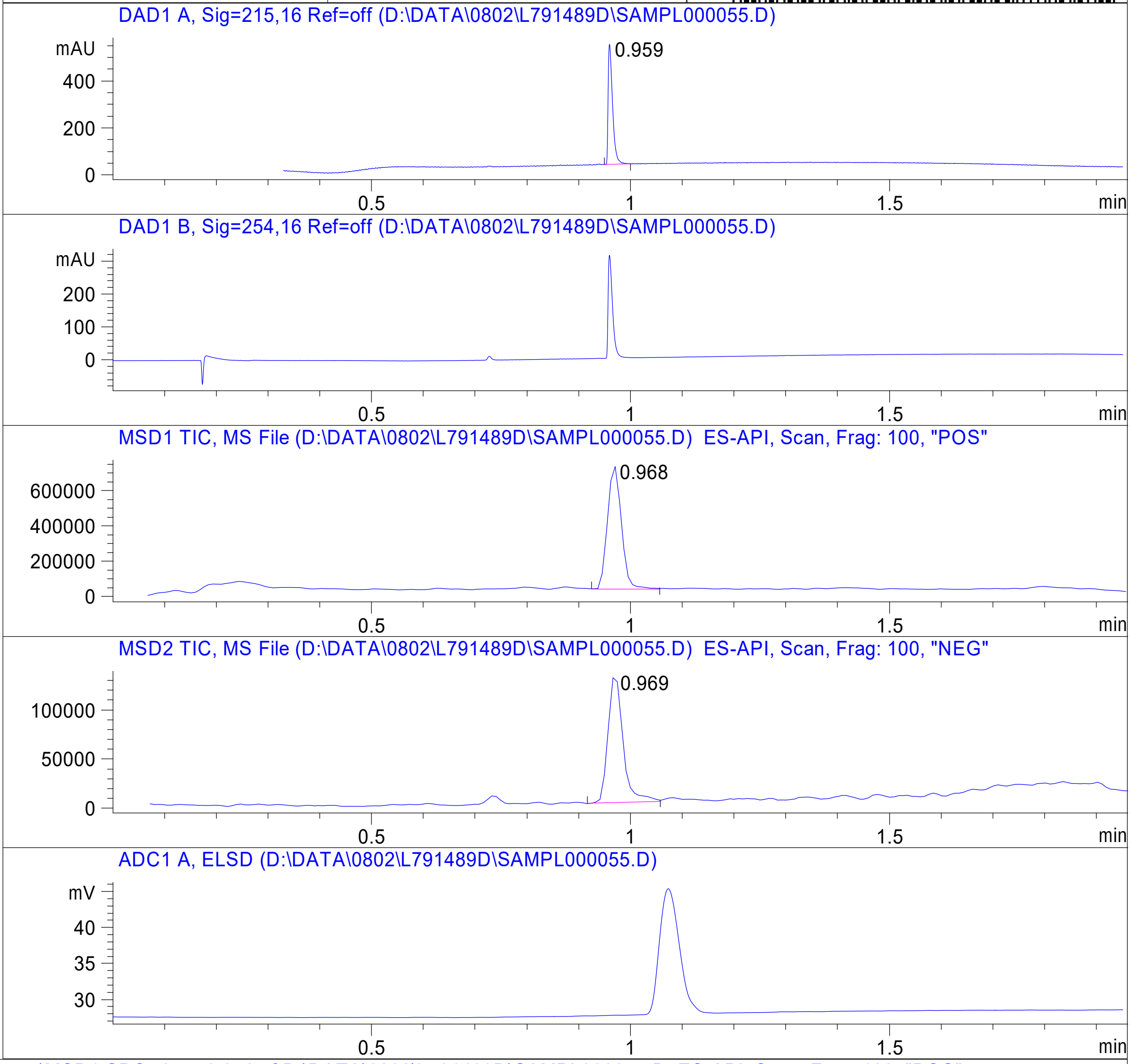

RT 0.968

RT 0.969

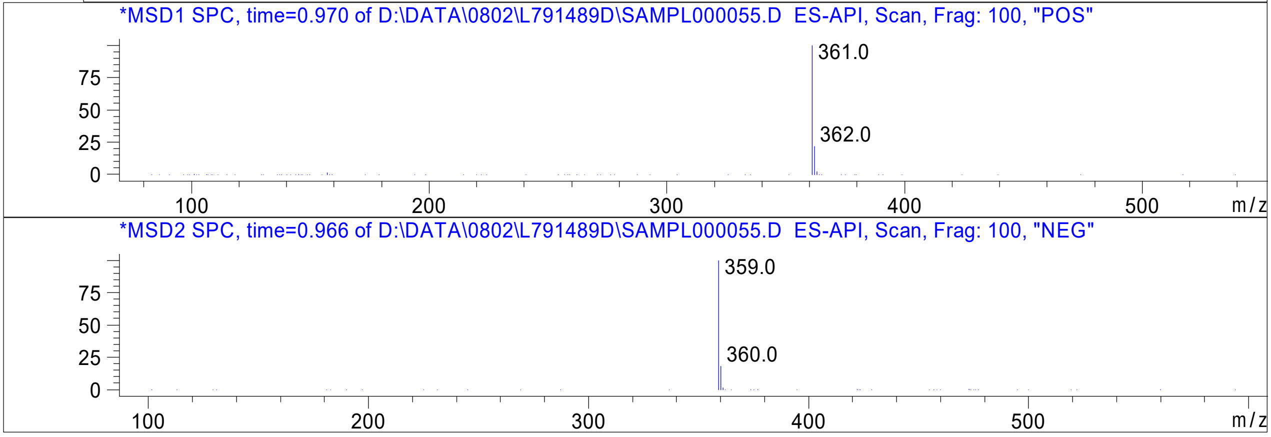

Inj.Date 8/1/2024 LB

C:\CHEM32\1\METHODS\SUPOR_30.M

MaxPeak: 100.00%
Ret_Time: 0.934 min

BE893340$2

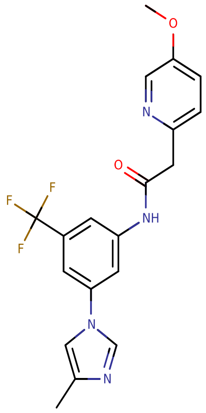

Mol Wt 390.36

Exact Mass 390.15

#	Time	Area%
1	0.934	100.00

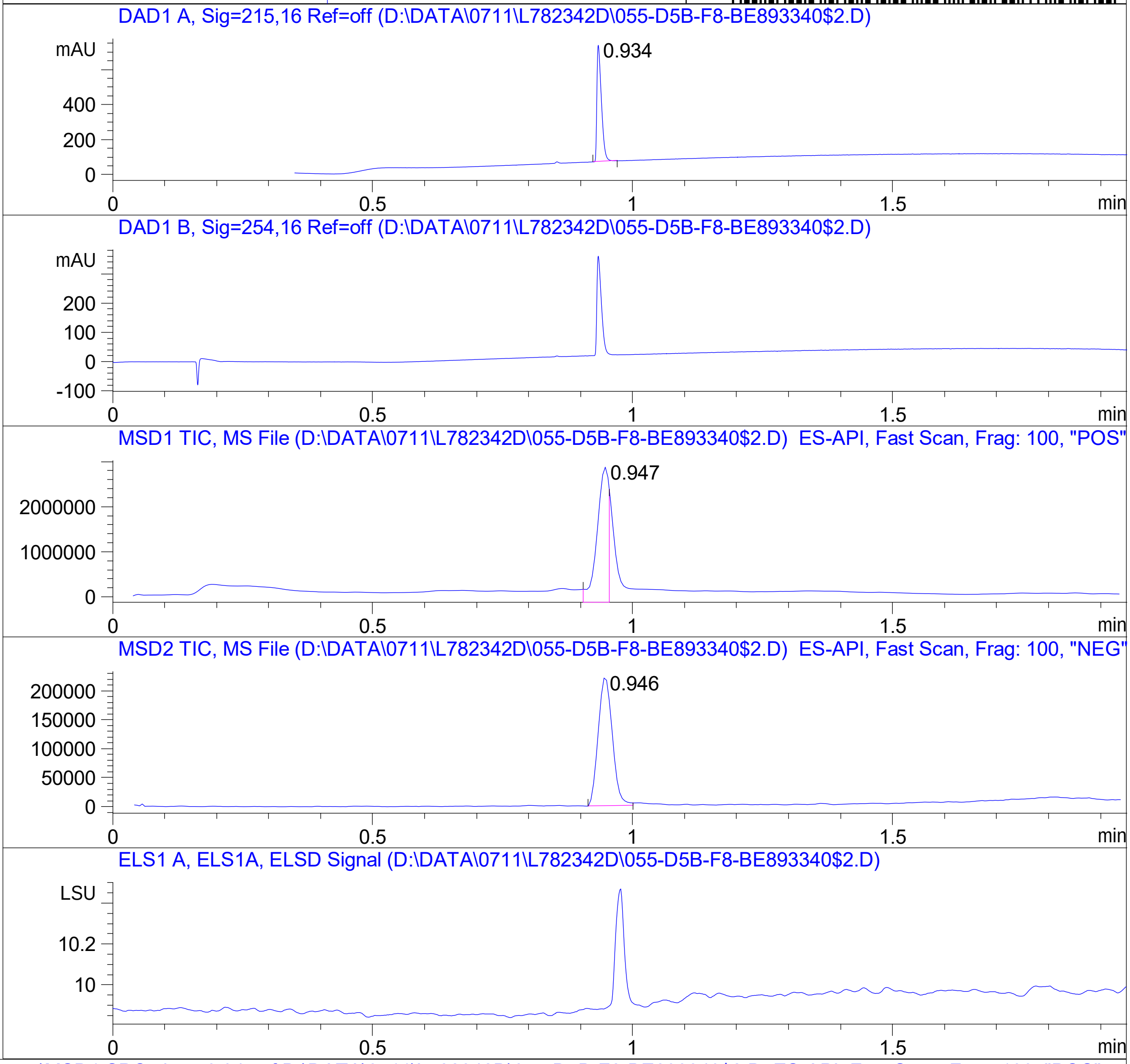

RT 0.947

RT 0.946

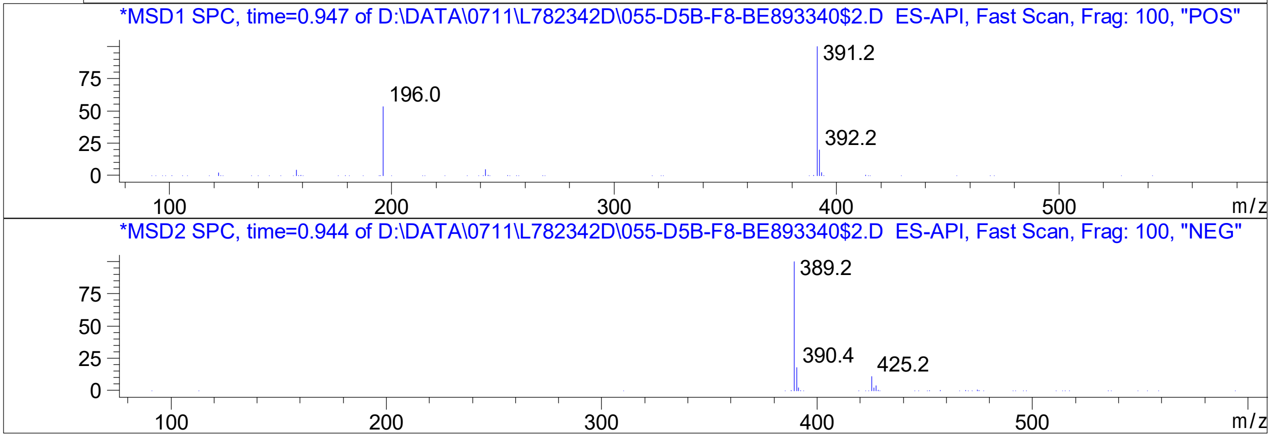

MaxPeak: 100.00%
Ret_Time: 1.116 min

BG236241$1

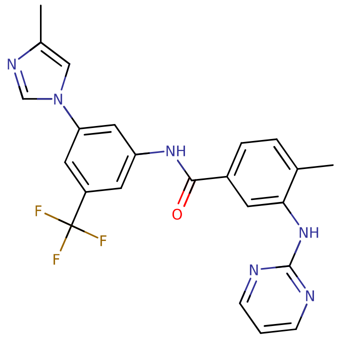

Mol Wt 452.43

Exact Mass 452.18

#	Time	Area%
1	1.116	100.00

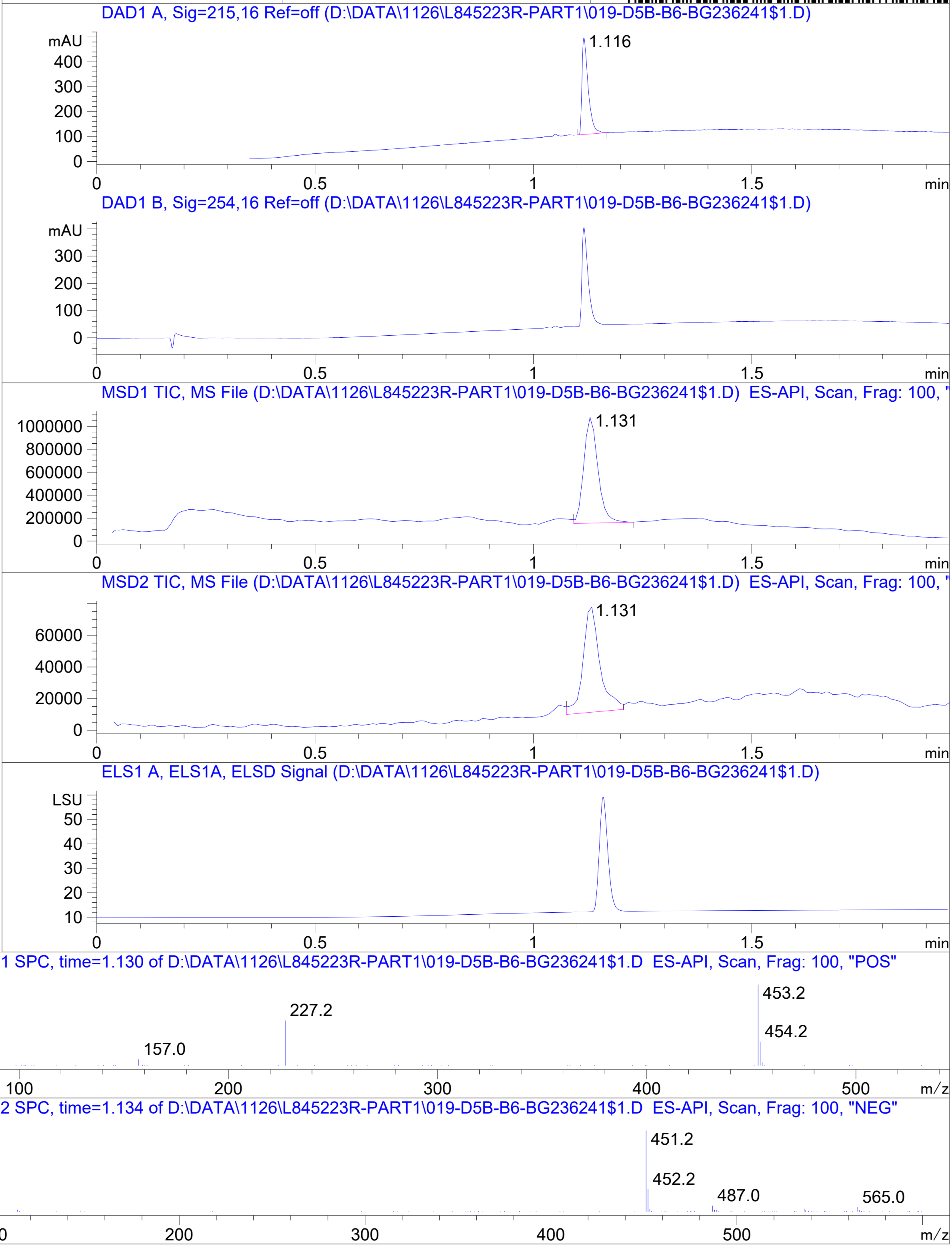

RT 1.131

RT 1.131

Inj.Date 11/26/2024 H 13

C:\Users\Public\Documents\ChemStation\1\Data\11_26\1845223R\SUPOR_30.M

BG236241$1

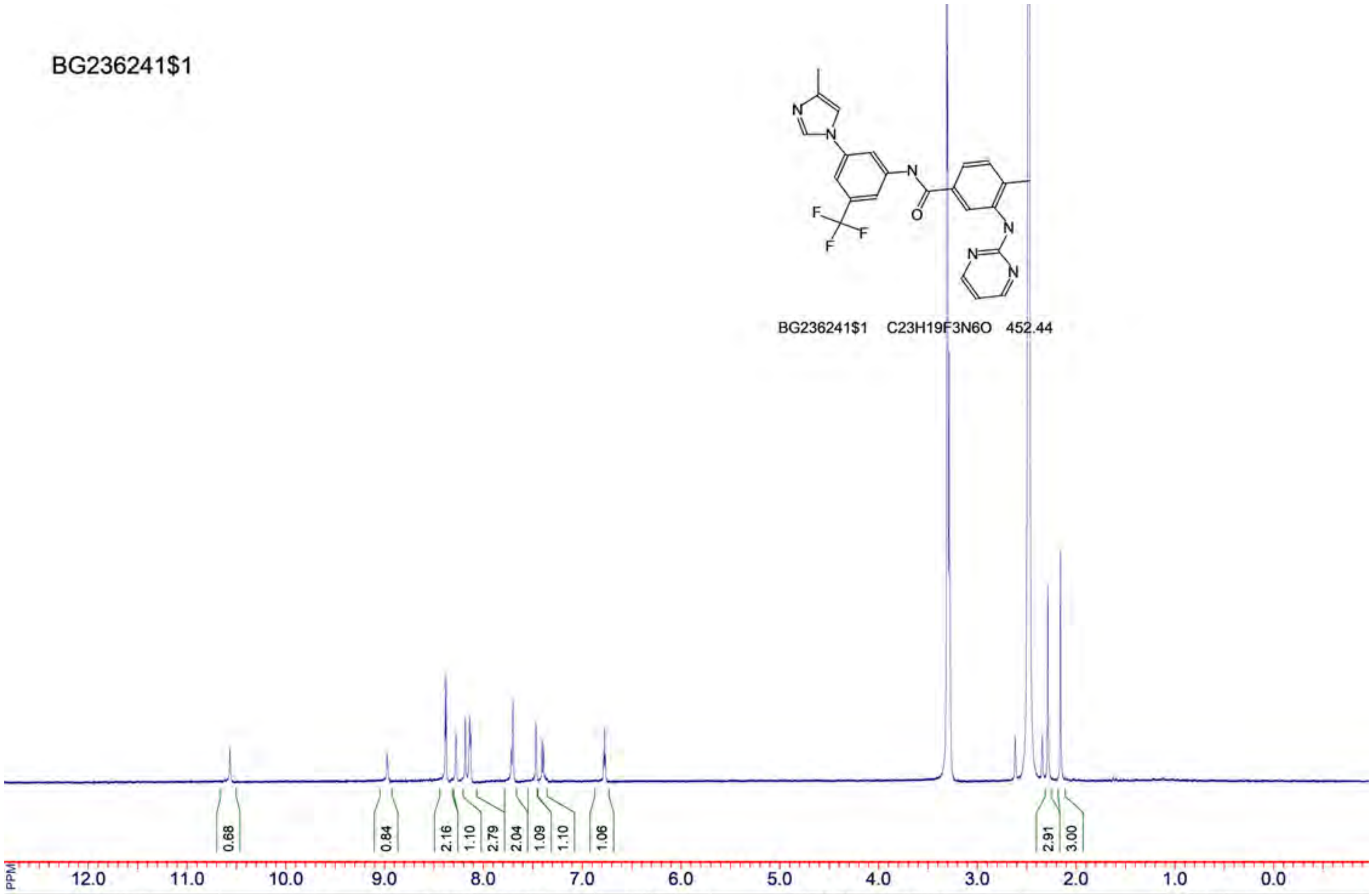

**BG236241$1**	Vybornov	SF: 499.8201 MHz	NSC: 0	PW: 9.00 usec, RG: 24	SI: 131072
Date: 27-Nov-2024	Solvent: dmso	SW: 8993 Hz	TE: 298 K	AQ: 3.56 sec, RD: 0.00 sec	BG236241$1

MaxPeak: 100.00%
Ret_Time: 1.086 min

BG285914$11

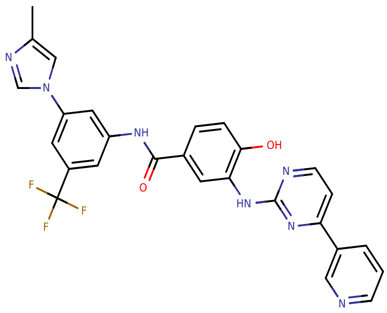

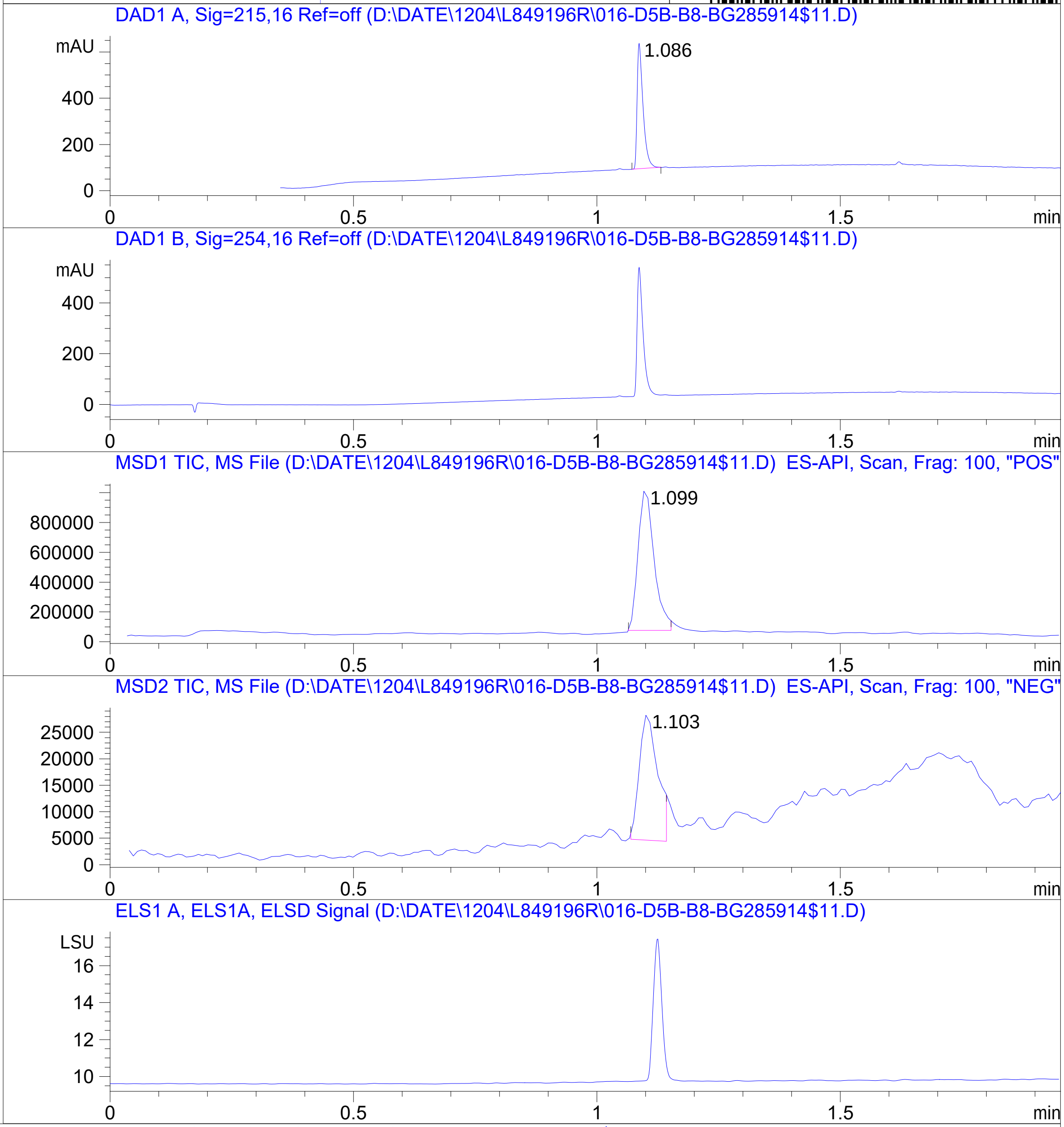

Mol Wt 531.49

Exact Mass 531.18

#	Time	Area%
1	1.086	100.00

RT 1.099

RT 1.103

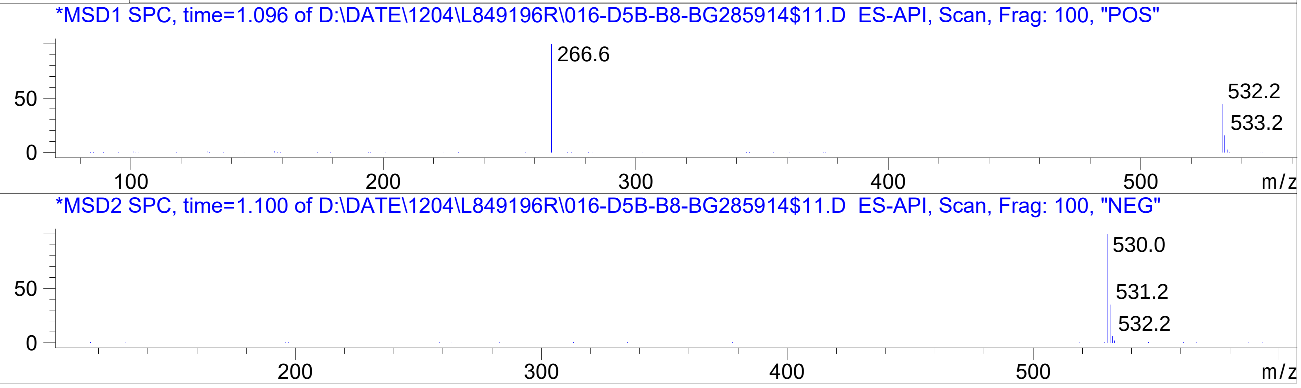

BG285914$11

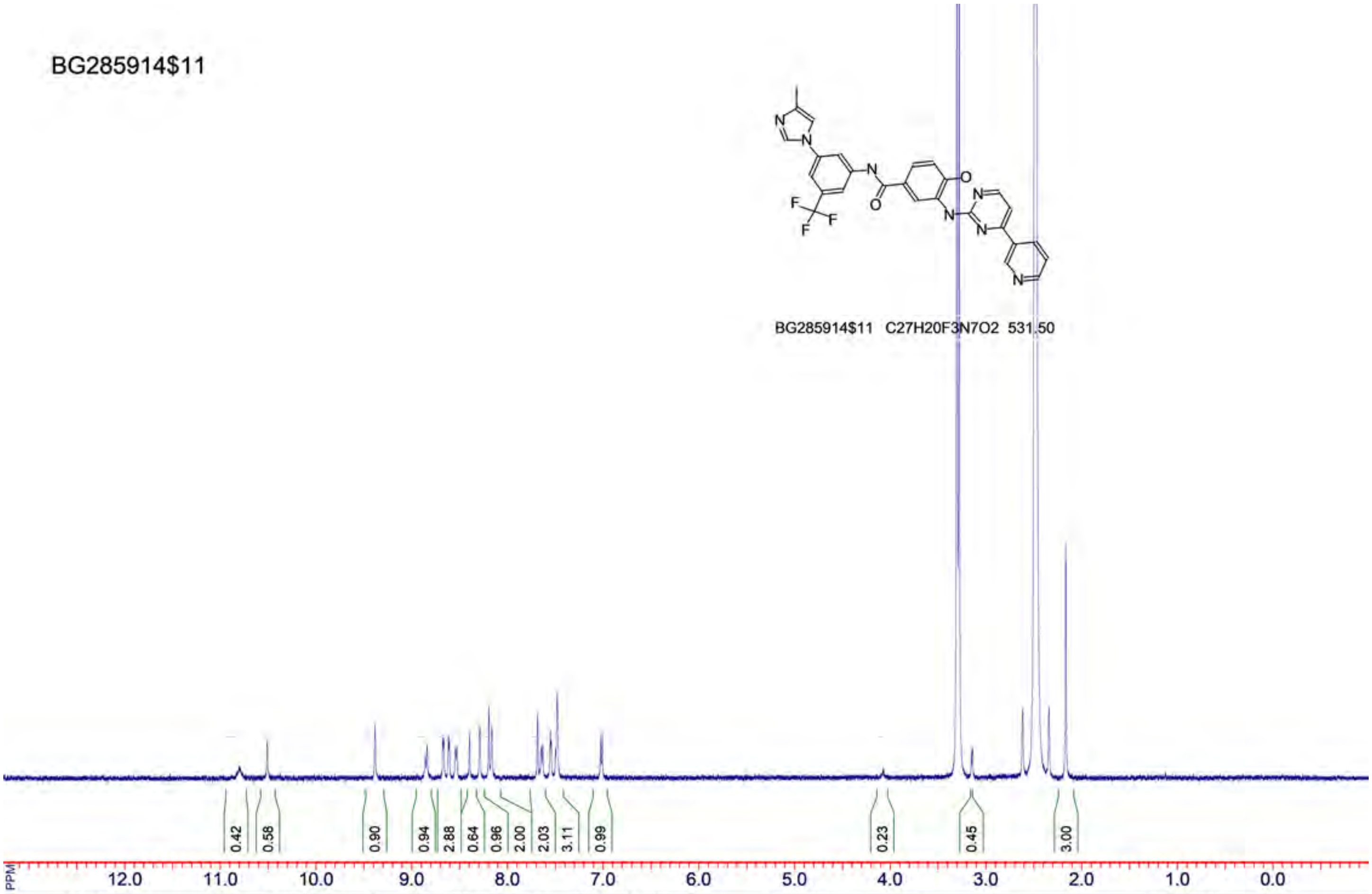

**BG285914$11**	Vybornov	SF: 499.8201 MHz	NSC: 0	PW: 9.00 usec, RG: 24	SI: 131072
Date: 05-Dec-2024	Solvent: dmso	SW: 8993 Hz	TE: 298 K	AQ: 2.67 sec, RD: 0.00 sec	BG285914$11
